# Supplementary material for: Earliest known Gondwanan bird tracks: Wonthaggi Formation (Early Cretaceous), Victoria, Australia
Source: PLoS One. 2023 Nov 15;18(11):e0293308. doi: 10.1371/journal.pone.0293308 (PMC10651008; doi:10.1371/journal.pone.0293308)
Supplement: S1 File — (DOCX) [file pone.0293308.s001.docx]

**Supporting Information 1: Description of Molding and Casting Process**

Molding fossil tracks or other trace fossils onsite enables for scientifically accurate copies to be produced while leaving the originals onsite. Molding is especially valuable for preserving primary information about trace fossils located in dynamic environments, such as those undergoing active weathering. Molds and casts can also augment other means of documentation, such as sketches, photographs, 3D scans, and photogrammetry.

In this study, most of the trace fossils were on marine-shore platforms covered by tides twice daily, hence the molds needed to be produced between these tides. The area to be molded was first swept clean of sediments and other loose debris, and then outlined with chalk. We usually allowed for a 10-cm wide border around footprints to provide context of the surrounding bedding plane. If time allowed, a small clay wall was placed around the molded area to contain the flow of silicone. However, owing to time limits between tides, walls were not used for most molds.

Before pouring the silicone, deep cracks or other areas where the silicone might run were identified and filled with a water-based clay. Otherwise the silicone would have filled these cracks and possibly caused damage to the original surface when removed. The molding medium used was Pinkysil**®,** a fast-set RTV (room temperature vulcanizing) silicone rubber. It is a two-component, addition-curing, and low-viscosity silicone designed for quickly taking impressions, which was ideal for molding tracks, as it is flows freely and sets in 15-20 minutes. After mixing both components of the silicone, it was poured over the selected area, but starting from higher points and allowed to flow. This application method works best, as it minimizes air bubbles in the silicone. However, a spatula or stiff brush, such as a toothbrush, were often needed to help cover all areas, which ensured a minimum of air trapped in depressions or undercuts on bedding-plane surfaces.

For small molds on a flat surface with a surrounding wall, it was possible to pour a thick enough layer to cover the entire surface with silicone. In such instances, the silicone set in about 20 minutes and was peeled off the molded surface. Nonetheless, most molds were made on sloping surfaces that varied in relief, hence different methods were needed for these. For example, on larger sloping surfaces, silicone was applied in multiple layers. After flowing, the first layer was often thin on surface high points. Once the first layer had set, a second layer was applied to these high points and also allowed to set. Then, a thixotropic agent (between 1-3%) was added to the silicone to make a stiff paste. The thickened silicone was then spread with a spatula over thin areas on high points or undercuts.

Before the silicone was removed, a fiberglass/polyester resin jacket was made to support the flexible silicone so it maintained an accurate shape of the footprints and surrounding rock surface once it was removed. A fiberglass jacket was applied directly to the silicone and released without the need for a release agent. Once set, the jacket was removed and the silicone peeled off the surface. Storing silicone molds in jackets was important for maintaining their accurate shapes.

Many products can be used to make copies from silicone molds. For example, we used fiberglass, which is relatively light and strong. The first coat of fiberglass applied to represent surface details was a polyester resin thickened with Premium Lite Fill™, a powdered perlite filler. Pigments were also added to the resin to provide a base color for painting. Once the thickened polyester resin had set, it was reinforced with chopped-stand fiberglass. Each cast was then trimmed and cleaned of imperfections and air bubbles, and painted to best approximate the original footprint-bearing surface.

Of the 27 tracks reported in this study, 22 were replicated via molding and casting, and thus are available for future study through Museum Victoria. These tracks are: FF-1-1, FF-1-3, FF-1-4, FF-3-1, FF-3-2, FF-3-3, FF-3-4, FF-3-5, FF-3-6, FF-4-3, FF-4-5, FF-5A-1, FF-5A-2, FF-5A-3, FF-5A-4, FF-5A-5, FF-5B-1, FF-5B-2, FF-5B-3, FF-5B-4, FF-5B-5, and HB-1.

References: Barnes Products, Technical Data Sheet; Pinkysil**®** Skin Safe-Fast Set Moulding System; RTV Silicone Rubber.
