# Supplementary material for: Earliest known Gondwanan bird tracks: Wonthaggi Formation (Early Cretaceous), Victoria, Australia
Source: PLoS One. 2023 Nov 15;18(11):e0293308. doi: 10.1371/journal.pone.0293308 (PMC10651008; doi:10.1371/journal.pone.0293308)
Supplement: S2 File — (PDF) [file pone.0293308.s002.pdf]

**Martin et al., Supporting Information 2:**
**Wonthaggi Formation Bird Tracks: Photos, Illustrations, Inferred Foot Morphologies, Ichnogenera, and Descriptions**

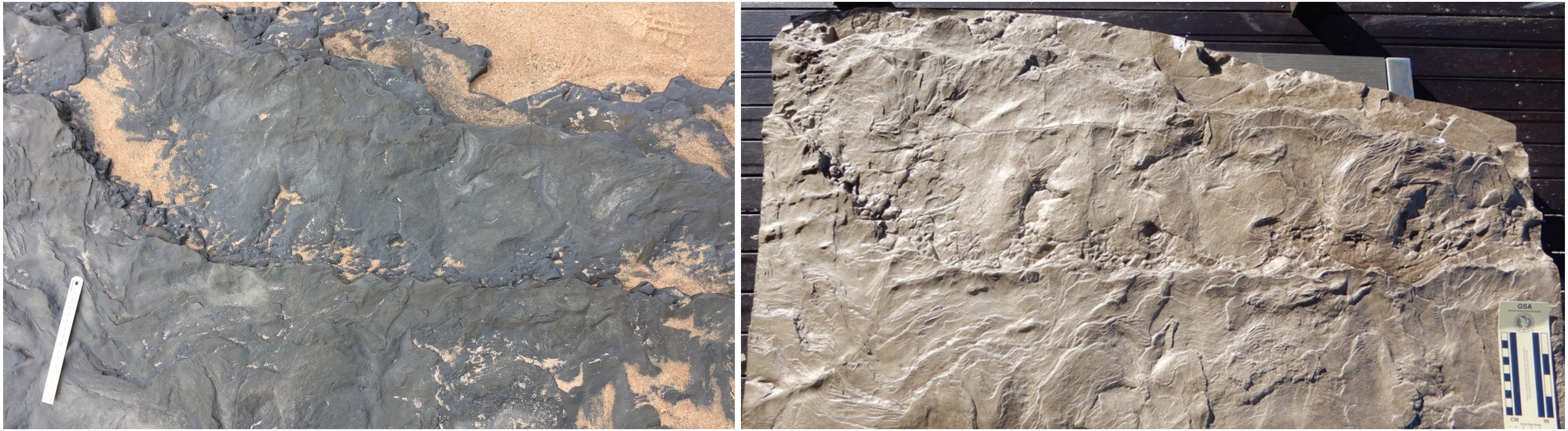


(Left): Outcrop view of bed FF-5 at Footprint Flats with tracks on surfaces FF-5A and FF-5B; photo taken in November 2020, scale = 15 cm long.
(Right) Polyester-resin cast of bed FF-5 with tracks on surfaces FF-5A and FF-5B; photo taken in May 2022, scale in centimeters. See Figure 4 for specimen numbers and track locations, Table 1 for measurements, and Table 2 for ichnogenera.

# Track HB-1: Honey Bay

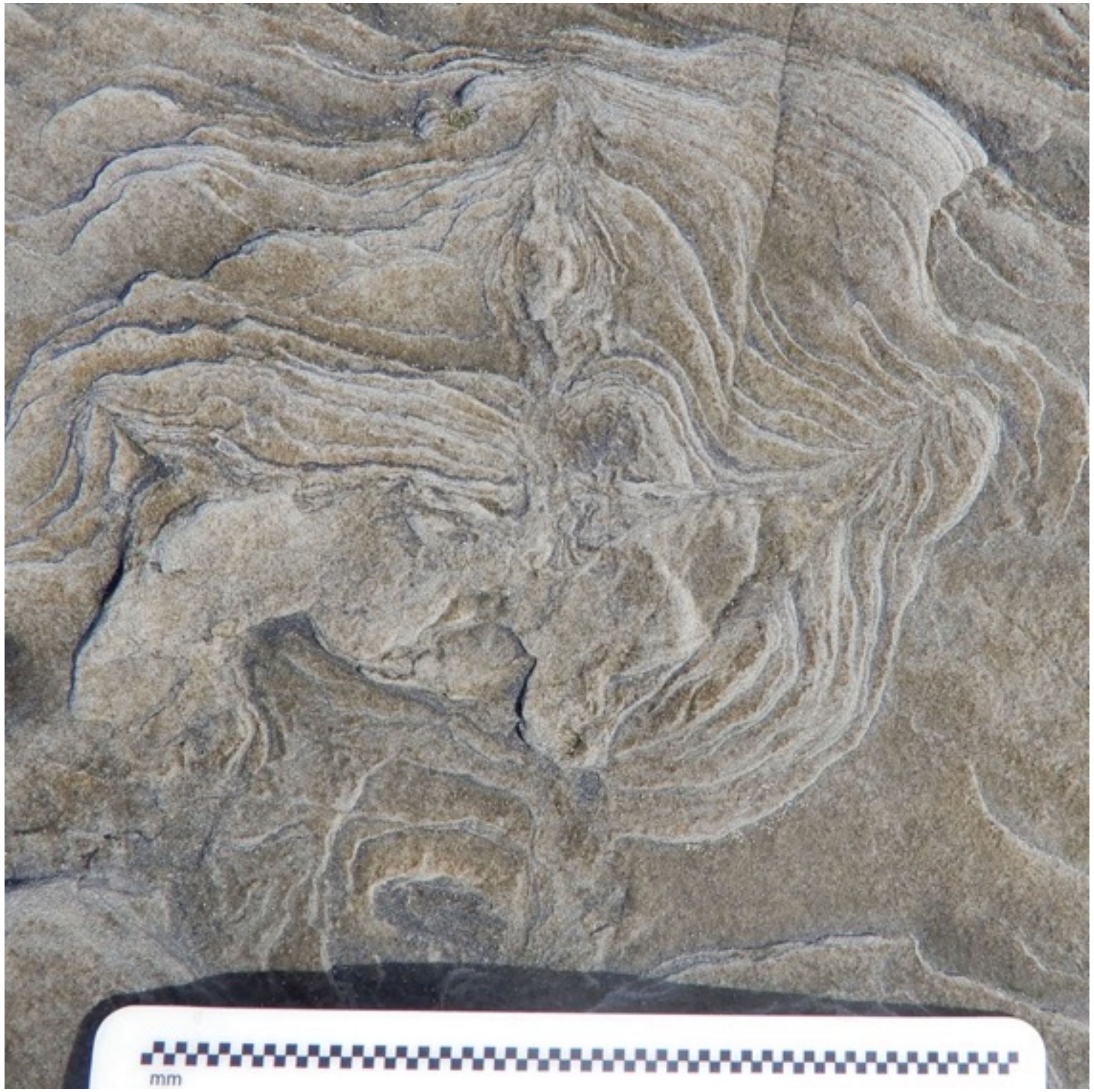


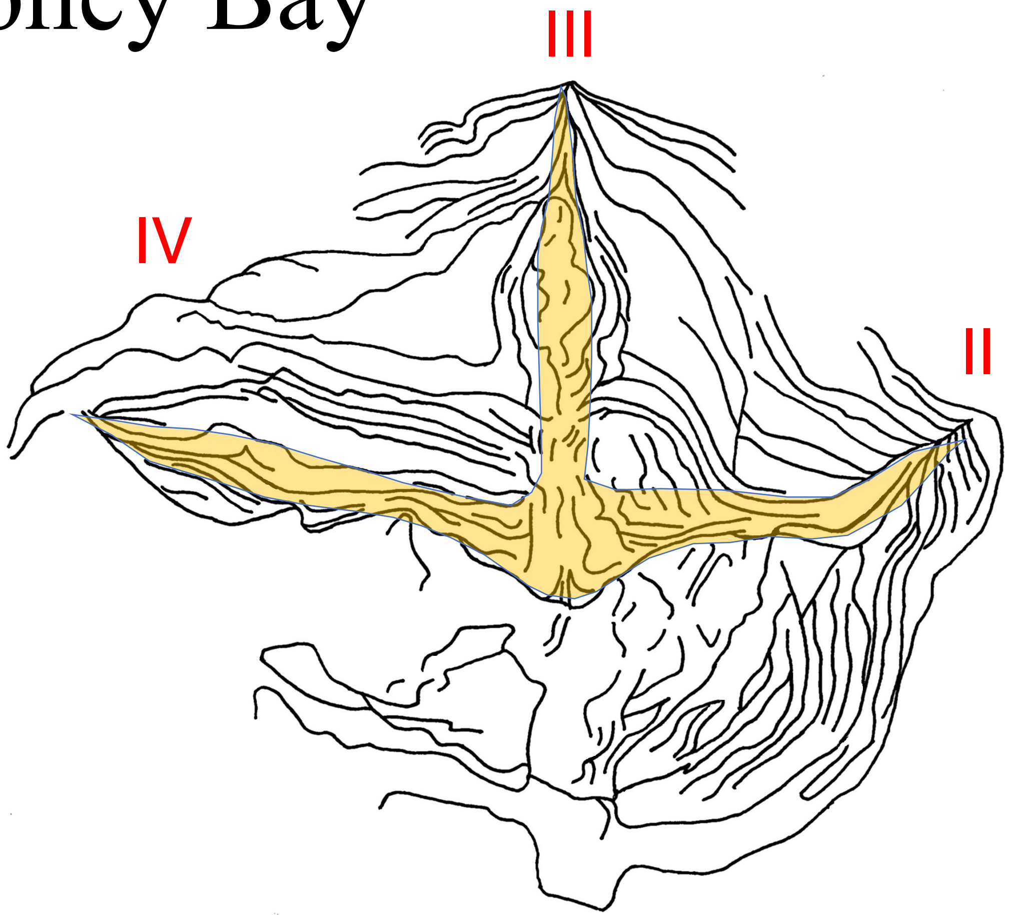


- Positive relief epichnion.
- Anisodactyl incumbent, but possible presence of digit I based on disturbed bedding posterior of digit III.
- Digits definable by disruption of underlying bedding.
- Sharp (narrow) claws.
- No proximal or distal webbing apparent.
- *Aquatilavipes?*

# Track FF-1-1: Footprint Flats

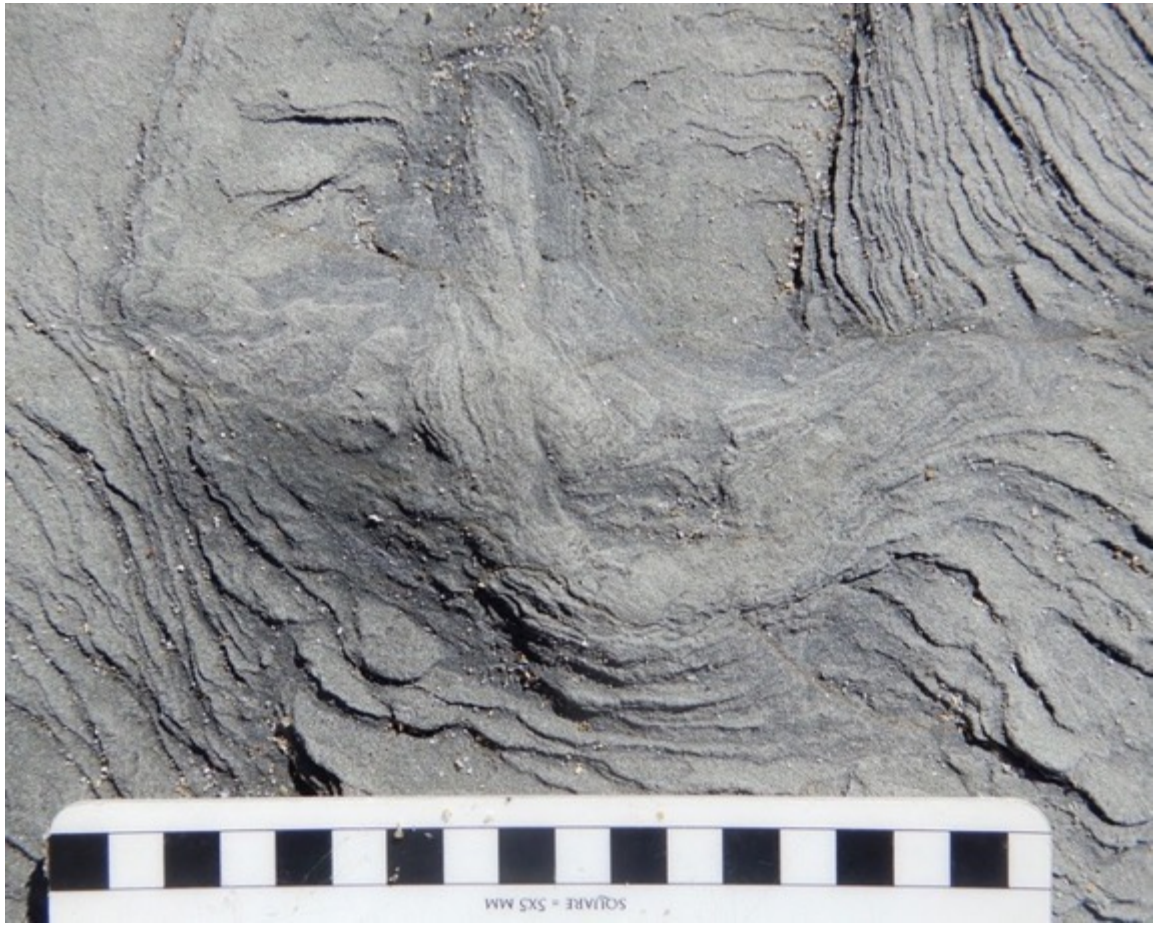

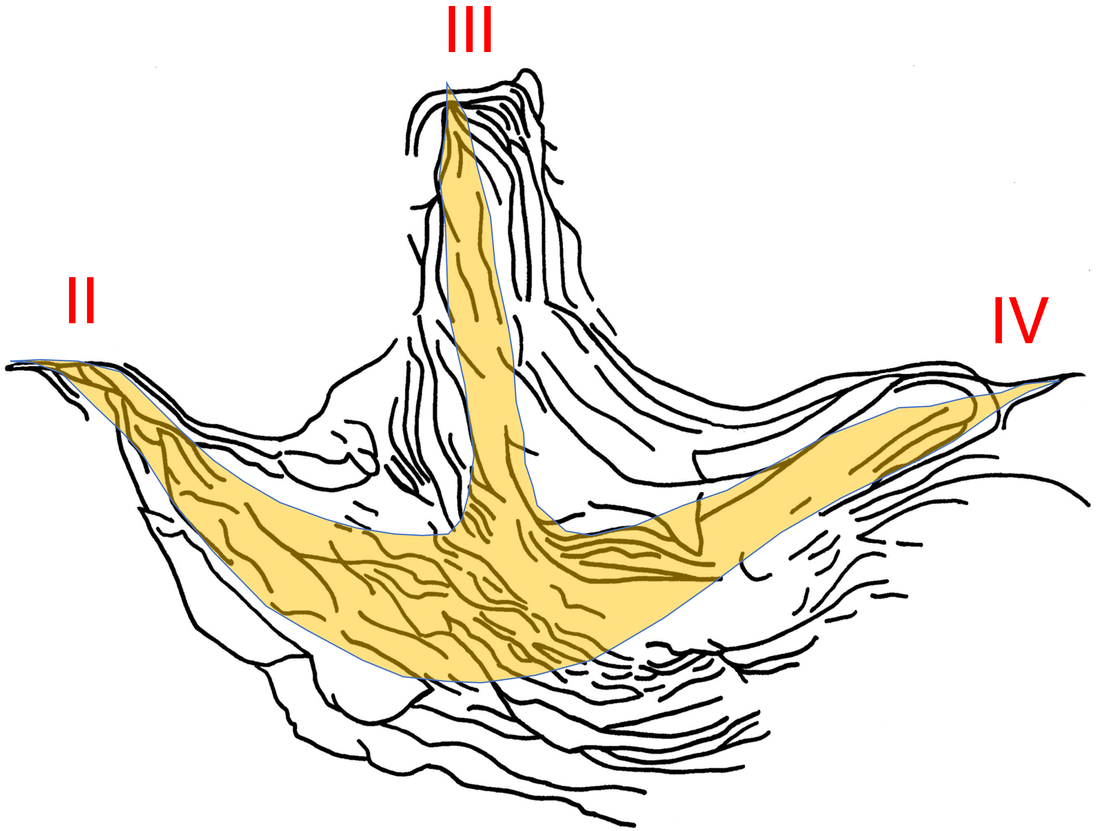


- Positive relief epichnion.
- Anisodactyl incumbent.
- Digits definable by disruption of bedding
- Sharp (narrow) claws, recurved on digits II and IV.
- Possible proximal webbing based on patterns between digits II-III and III-IV.
- *Aquatilavipes?*

# Track FF-1-2: Footprint Flats

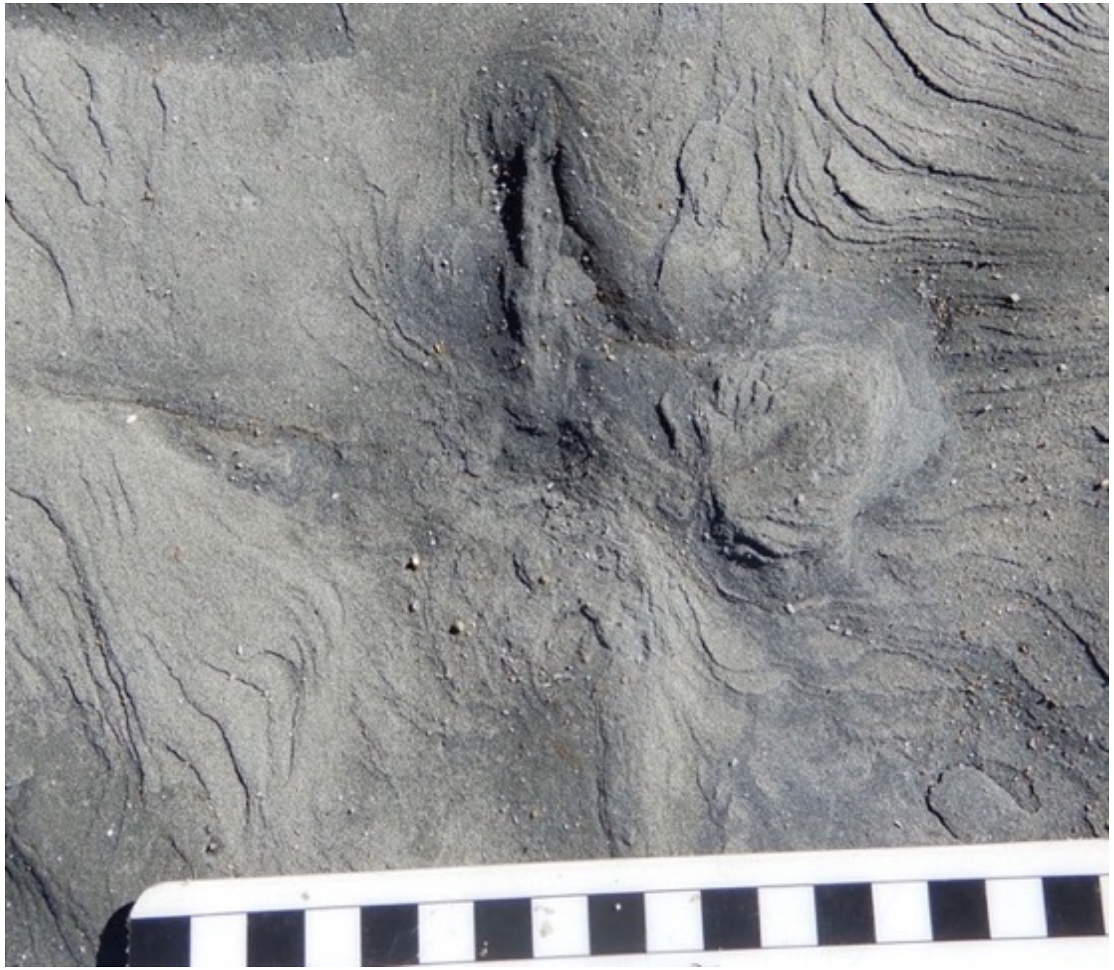

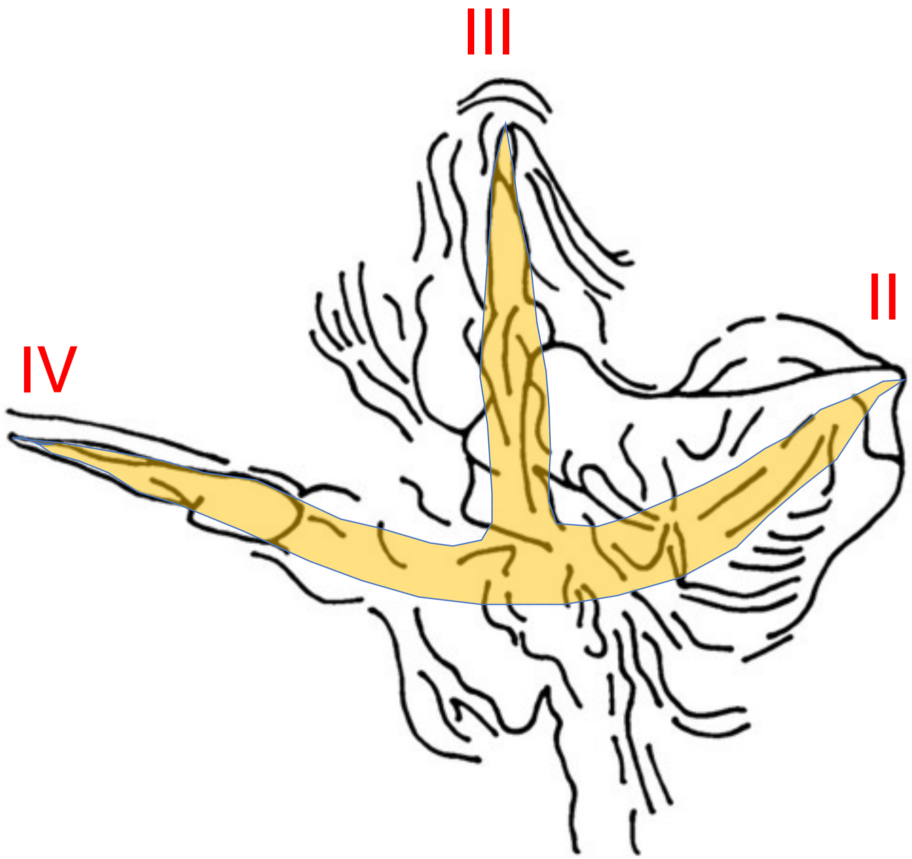


- Positive relief epichnion.
- Anisodactyl incumbent, but possible presence of digit I based on disturbed bedding posterior of digit III.
- Digits definable by disruption of bedding.
- Sharp (narrow) claws.
- No webbing apparent.
- *Aquatilavipes?*

# Track FF-1-3: Footprint Flats

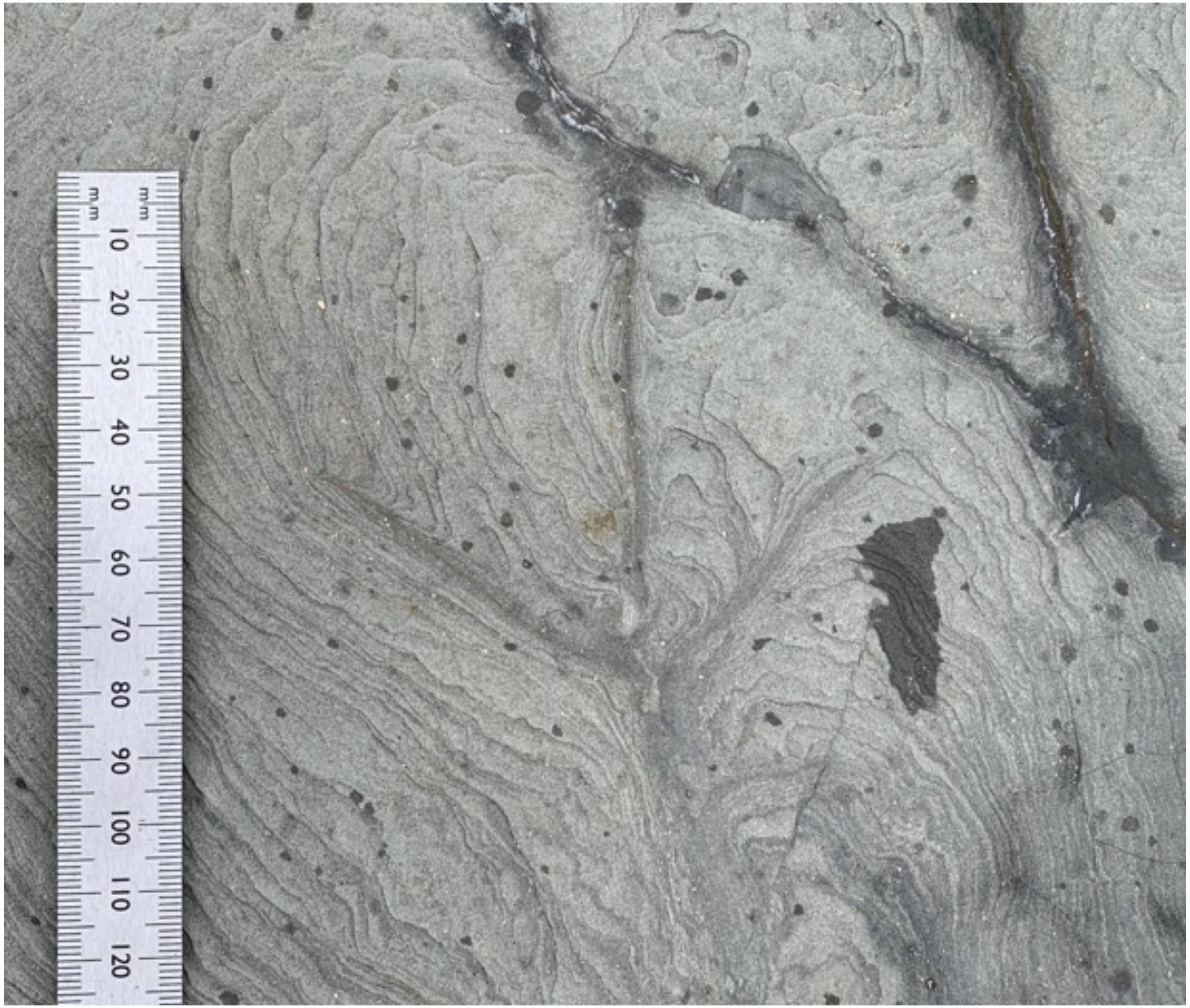

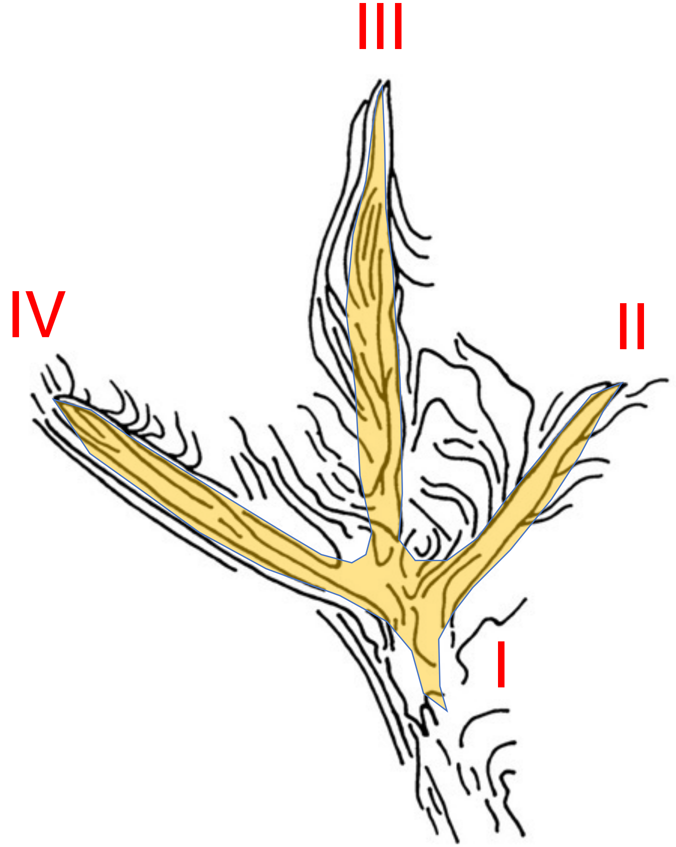


- Negative relief epichnion.
- Anisodactyl.
- Digits definable by disruption of bedding.
- Sharp (narrow) claws, recurved on digit II.
- No webbing apparent.
- *Ardeipeda?*

# Track FF-1-4: Footprint Flats

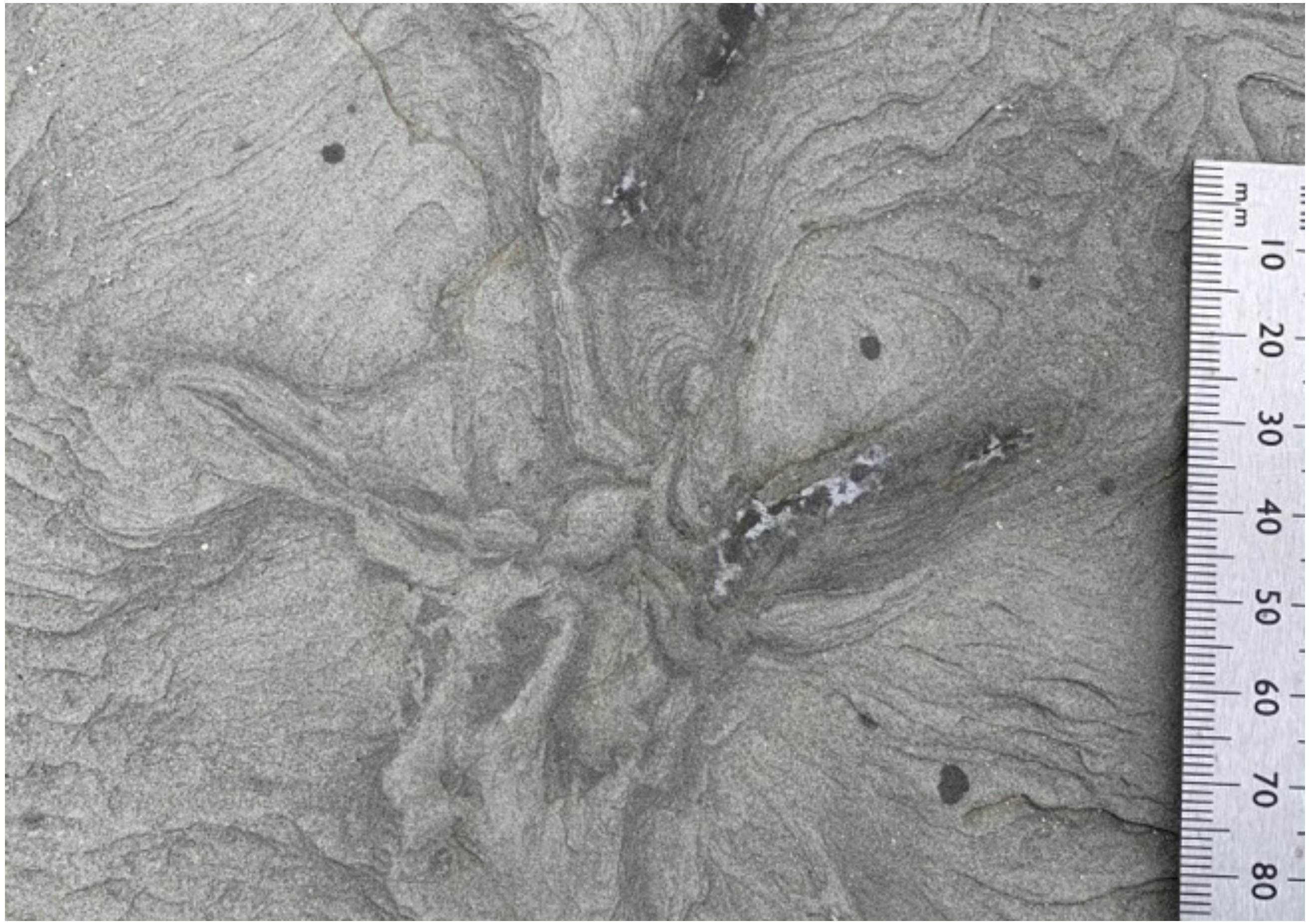


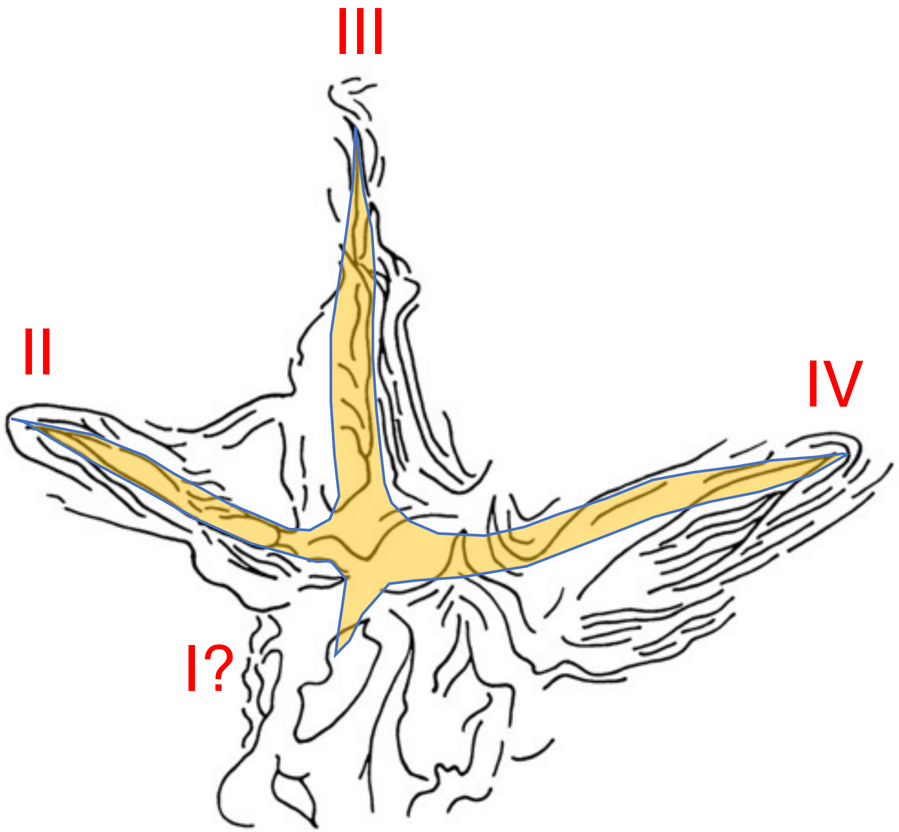


- Negative relief epichnion
- Anisodactyl incumbent, but probable presence of digit I based on disturbed bedding posterior to digit III.
- Digits definable by disruption of bedding.
- Sharp (narrow) claws, recurved on digits II and IV.
- No webbing apparent.
- *Ardeipeda?*

# Track FF-2-1: Footprint Flats

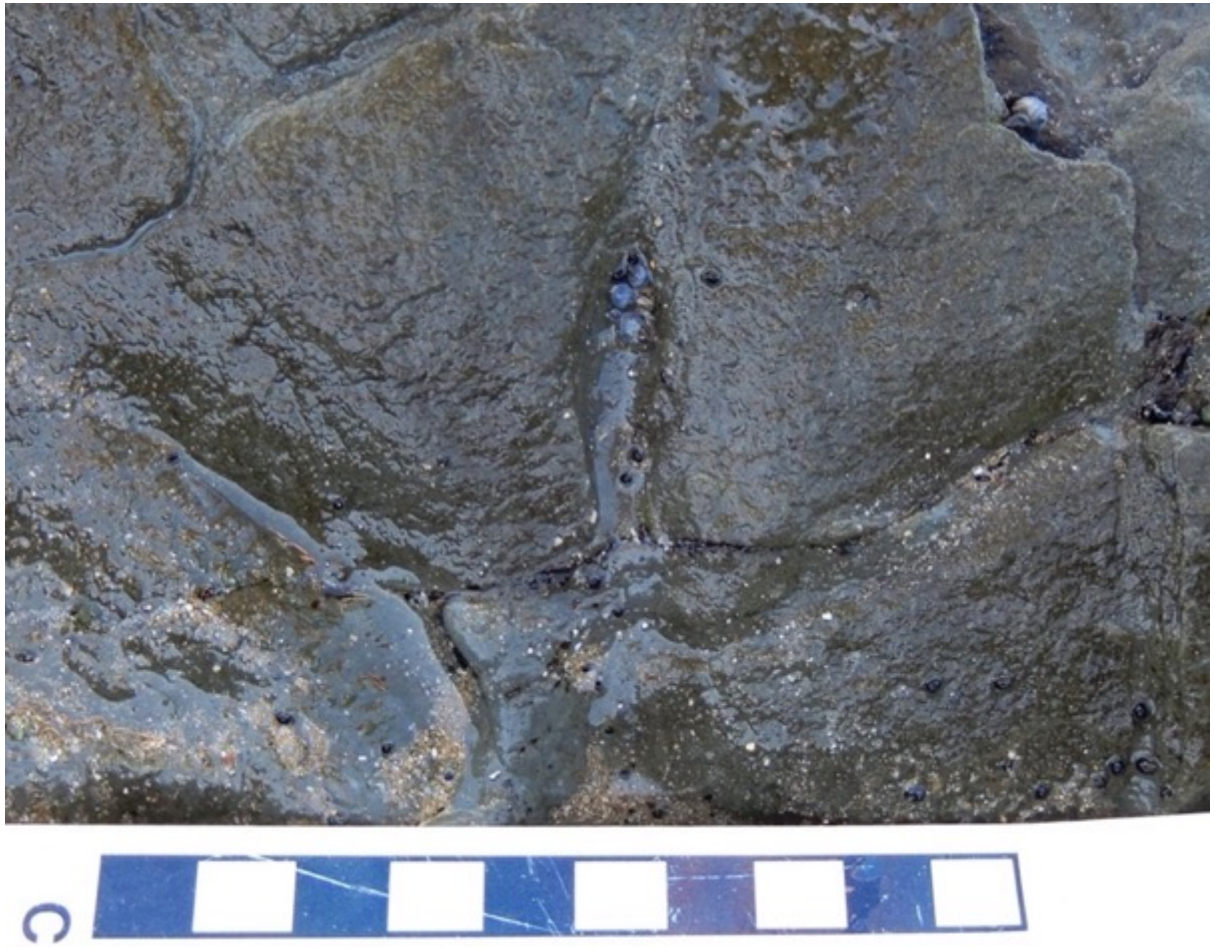

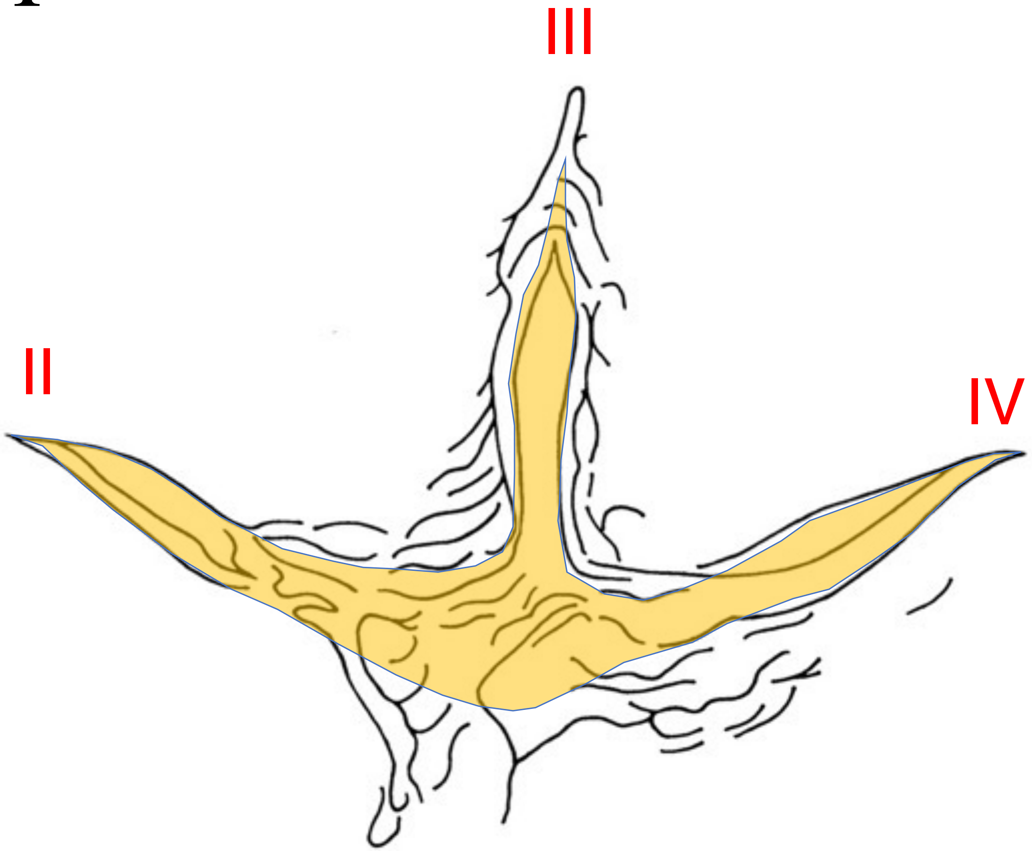


- Negative relief epichnion.
- Anisodactyl incumbent.
- Sharp (narrow) claws, recurved on digits II and IV.
- Possible proximal webbing between digits II-III and III-IV.
- *Avipeda?*

# Track FF-3-1: Footprint Flats

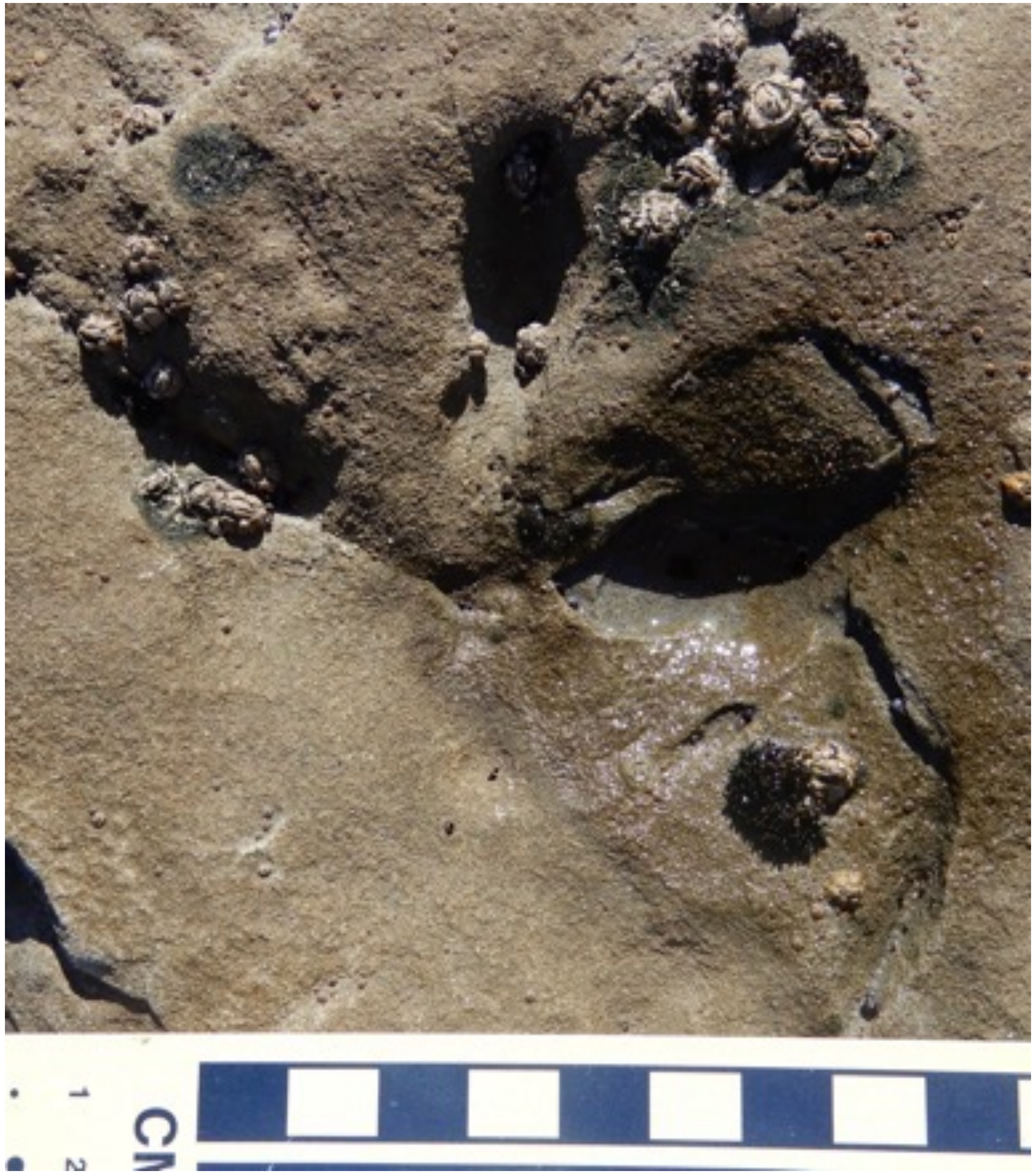


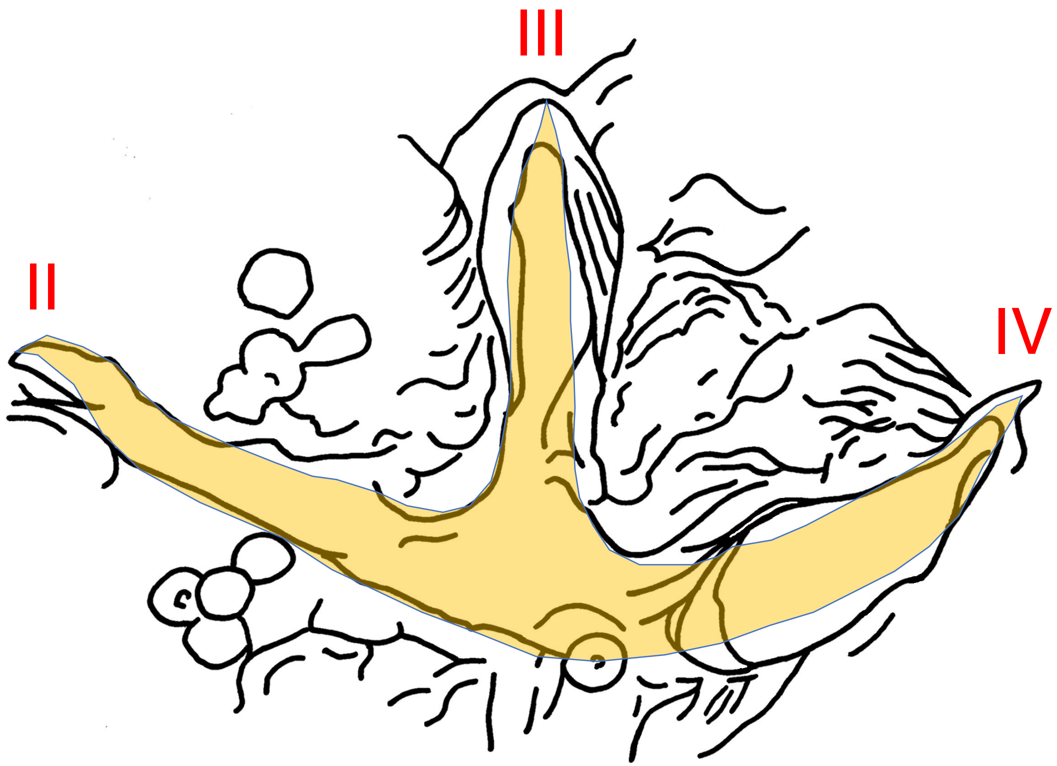


- Negative relief epichnion.
- Anisodactyl incumbent.
- Digit impressions co-occur with invertebrate burrows, but also affected modern erosion and bioencrusters.
- Claws poorly defined.
- Possible webbing between digits II-III and III-IV.
- *Wupus*?

# Track FF-3-2: Footprint Flats

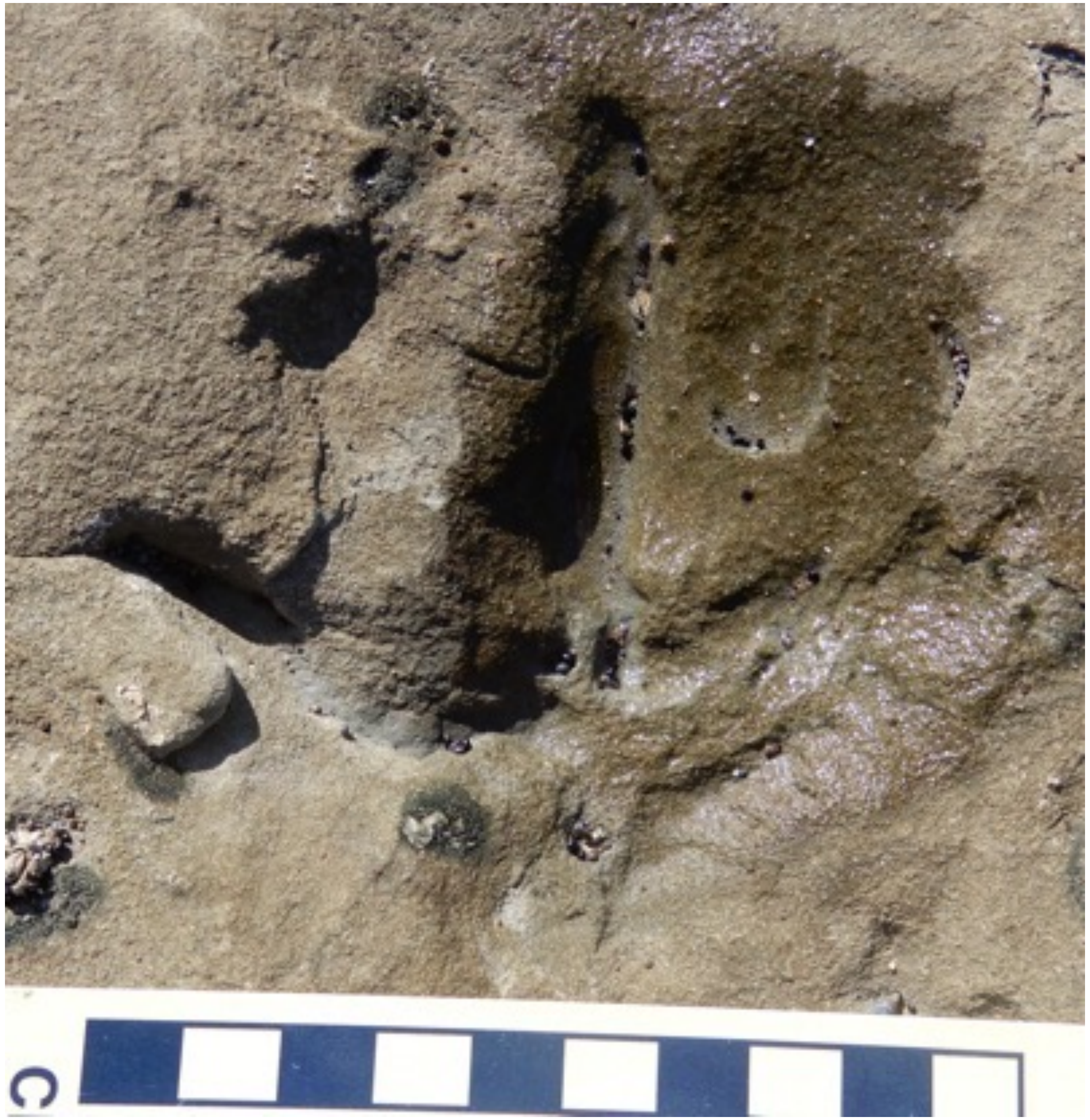

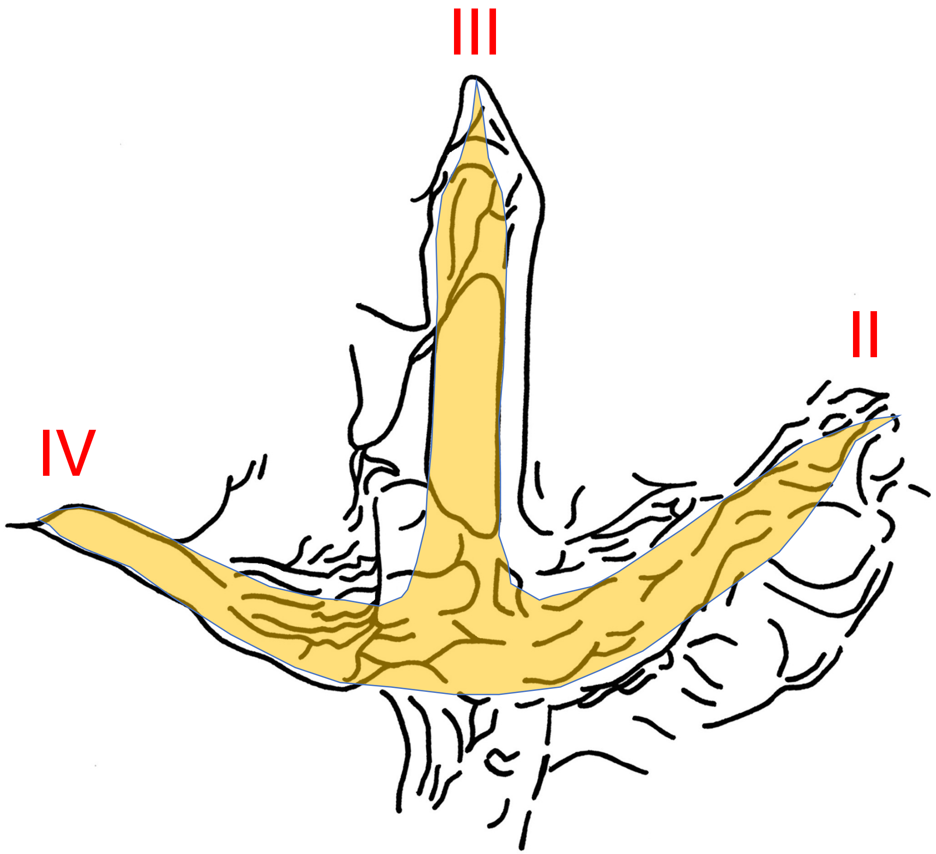


- Negative relief epichnion
- Anisodactyl incumbent.
- Digits widened by erosion.
- Claws poorly defined.
- Possible proximal webbing between digits II-III and III-IV.
- *Fuscinapeda?*

# Track FF-3-3: Footprint Flats

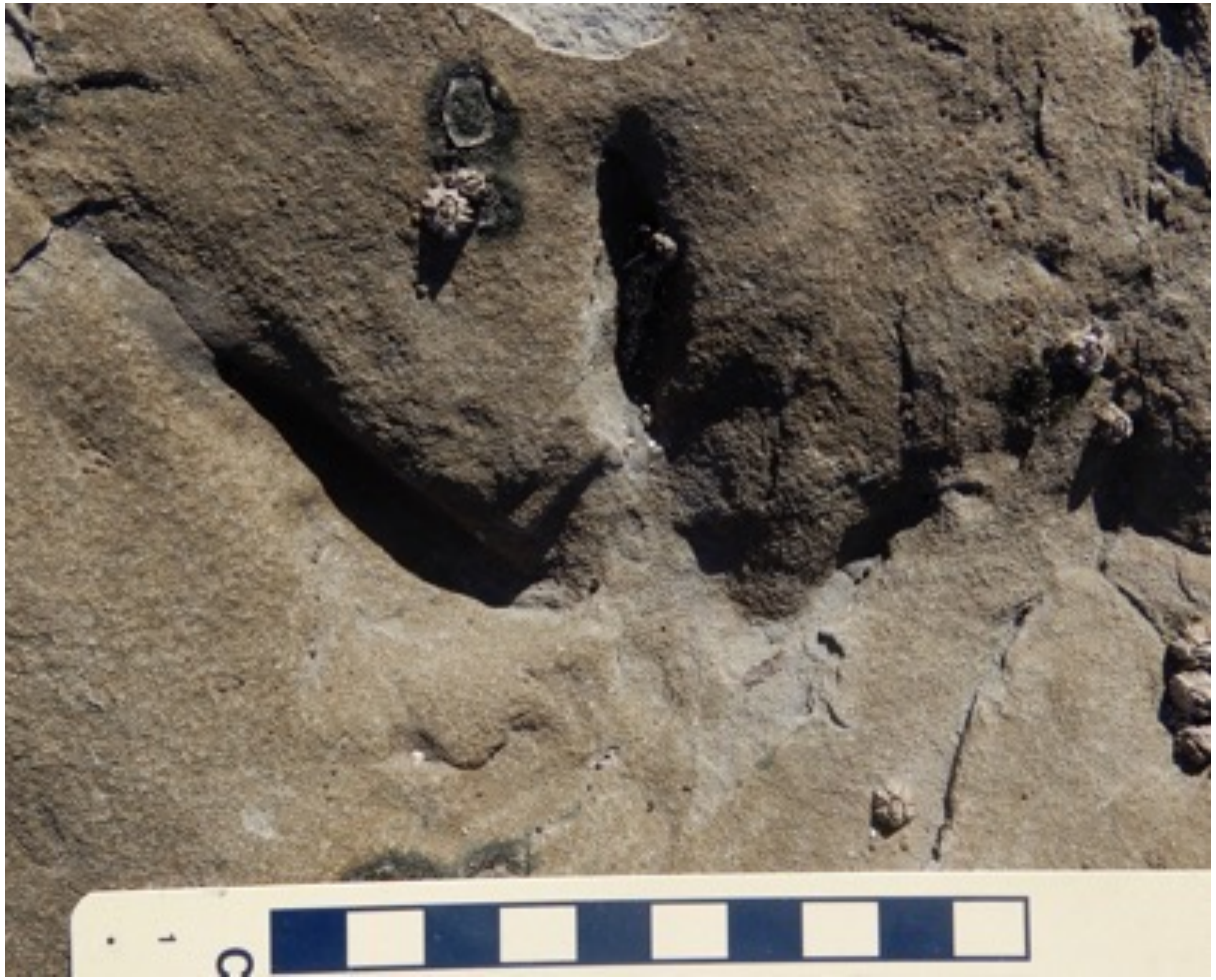

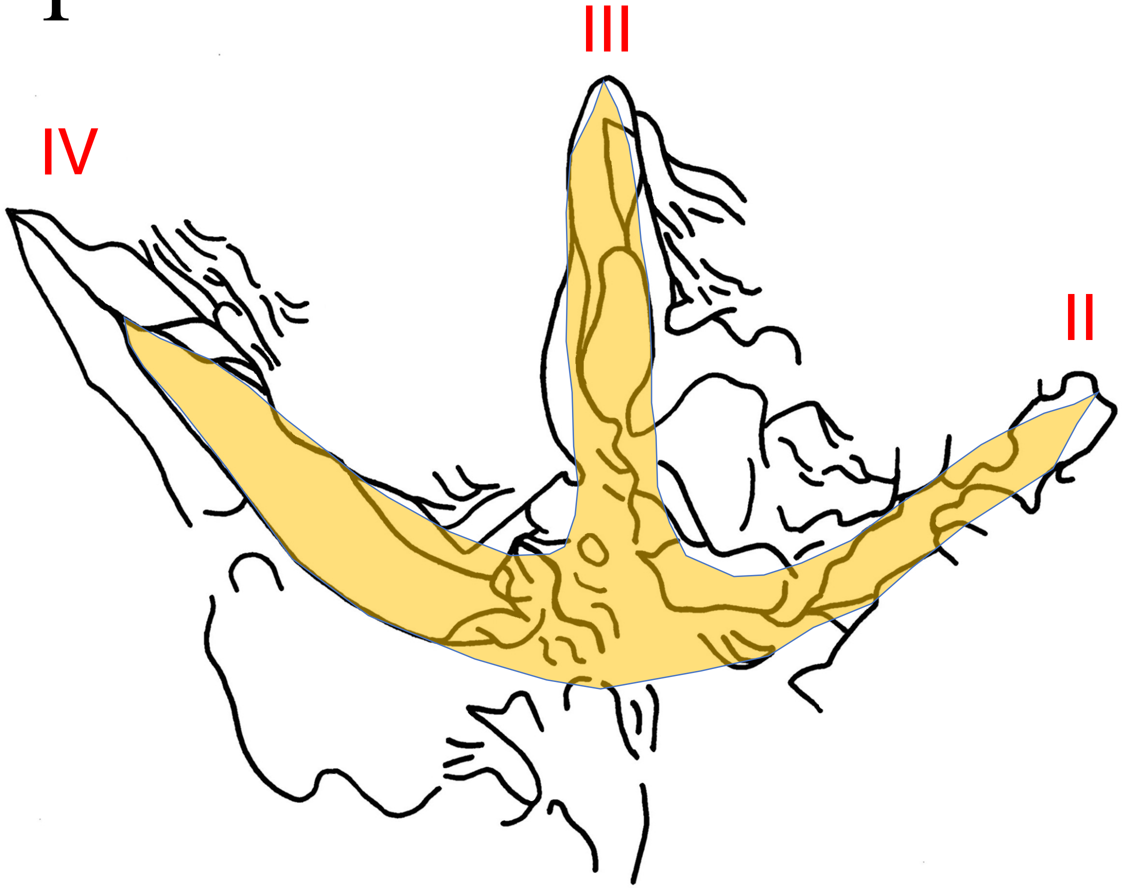


- Negative relief epichnion.
- Anisodactyl incumbent.
- Sedimentary structure (ridge) behind digit IV.
- Digits widened by erosion.
- Claws and digit I poorly defined.
- Possible proximal webbing between digits II-III and III-IV.
- *Fuscinapeda*?

# Track FF-3-4: Footprint Flats

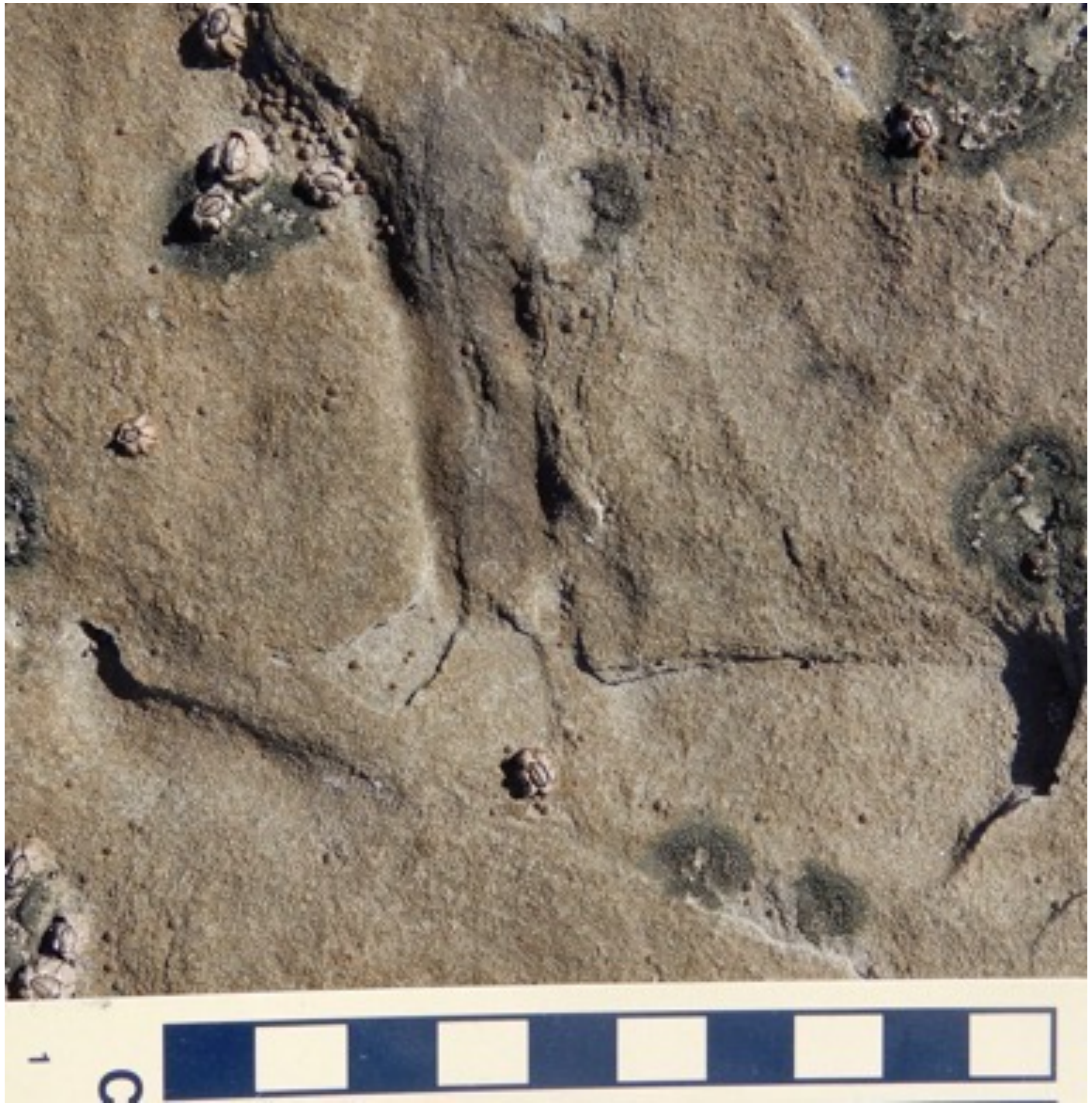

(No drawing)

- Negative relief epichnion.
- Anisodactyl incumbent, but missing one lateral digit (II or IV).
- Digits widened by erosion.
- Claw poorly defined on digit III but visible on lateral digit.
- Possible proximal webbing between lateral digit and digit III.
- Ichnogenus not determined.

# Track FF-3-5: Footprint Flats

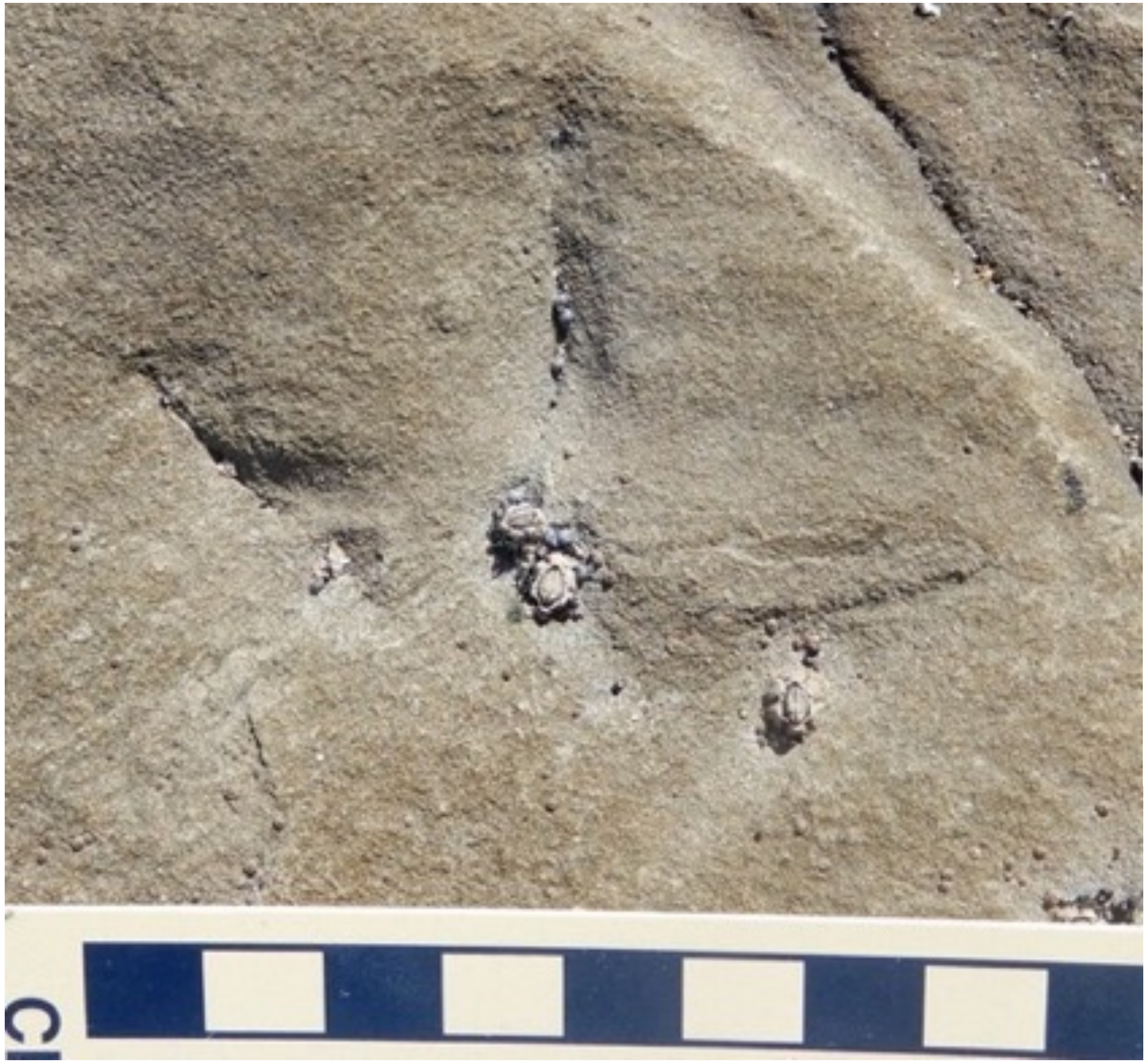

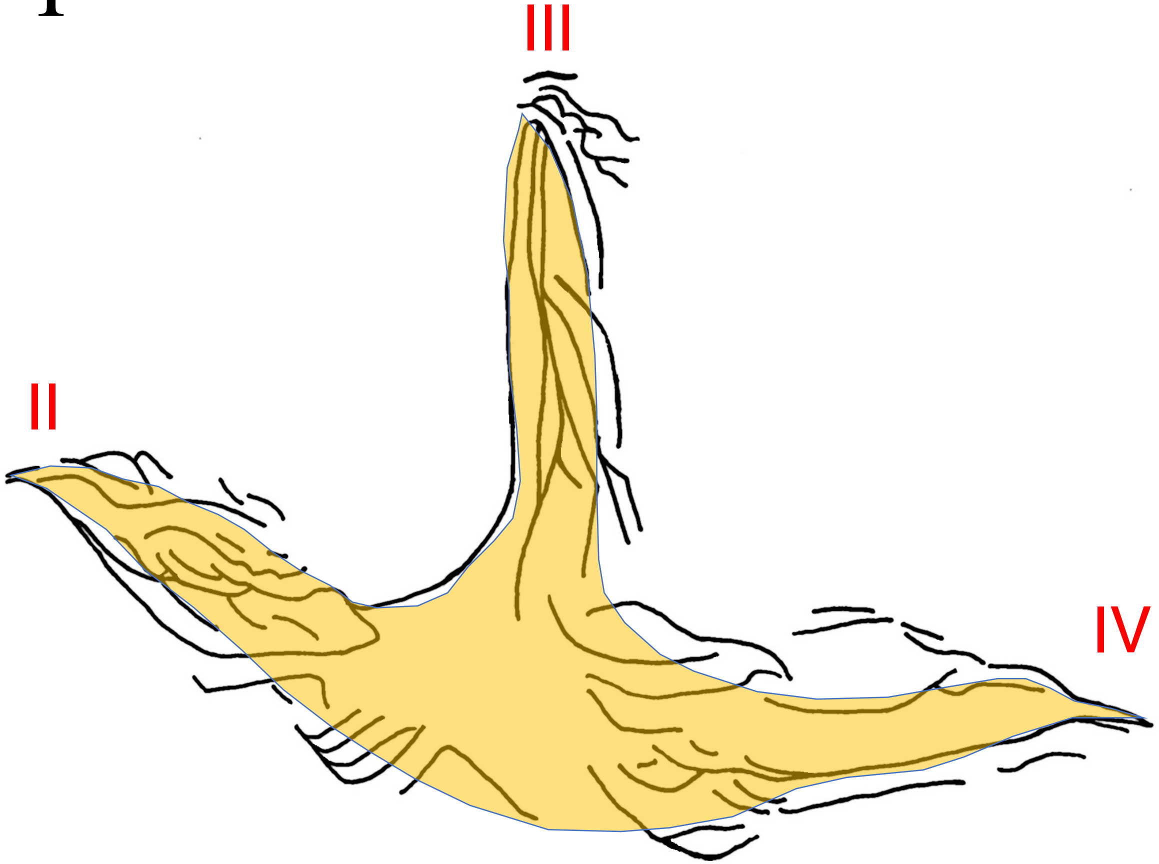


- Negative relief epichnion.
- Anisodactyl incumbent.
- Digits definable by disruption of bedding.
- Sharp (narrow) claws, recurved on digits II and IV.
- Possible proximal webbing between digits II-III and III-IV.
- *Avipeda?*

# Track FF-3-6: Footprint Flats

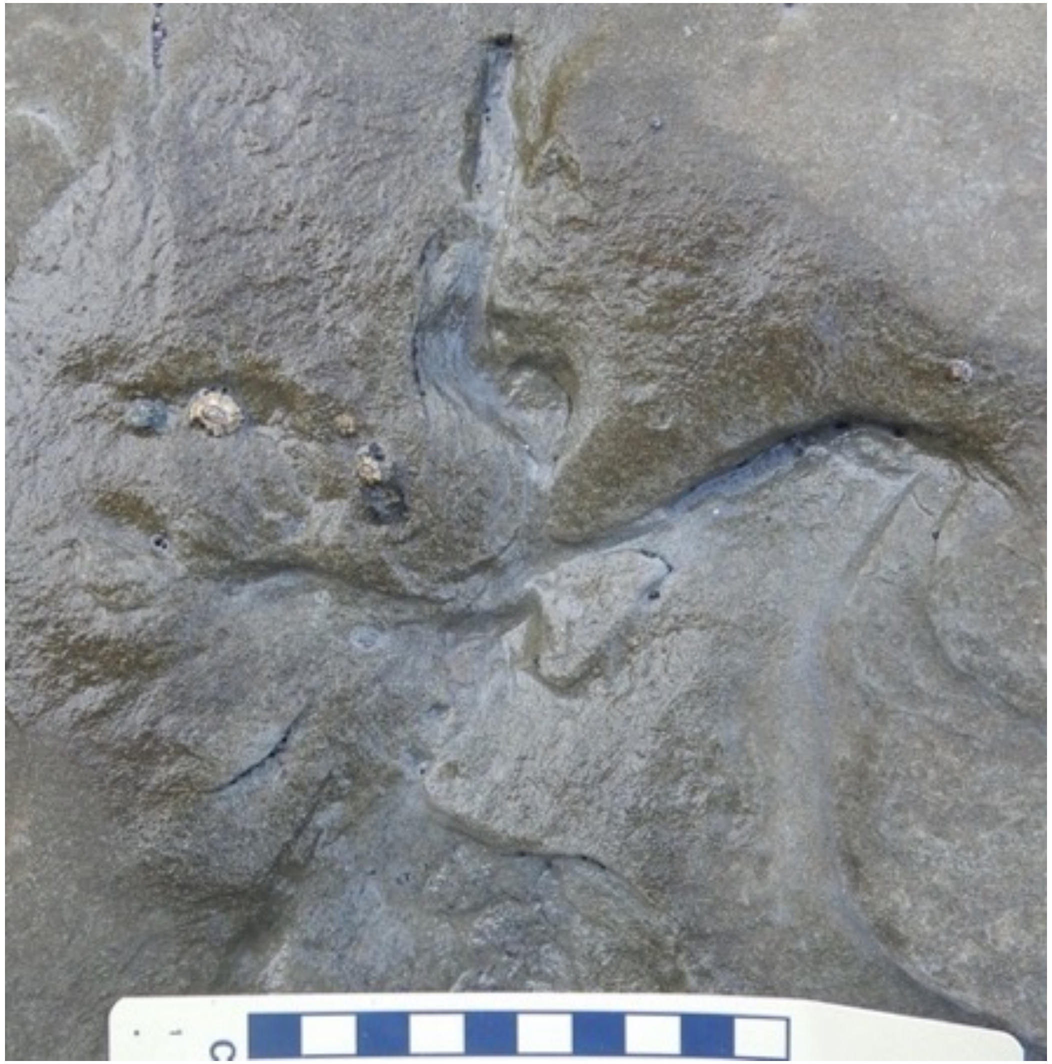

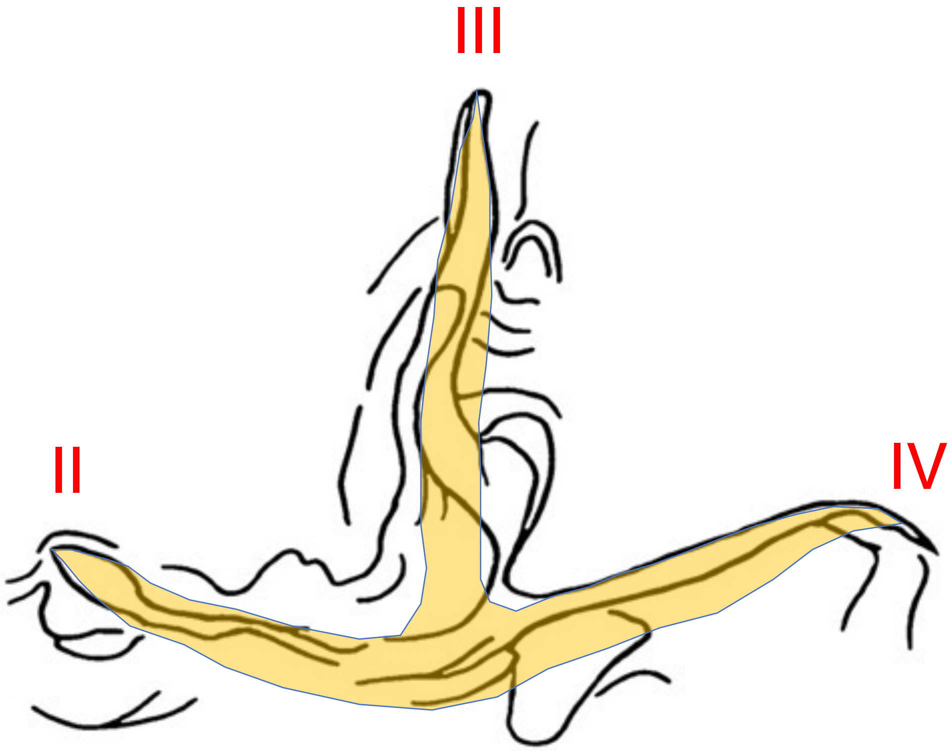


- Negative relief epichnion.
- Anisodactyl incumbent.
- Digits definable by disruption of bedding.
- Digits “bent,” perhaps by saturated sediments?
- Sharp (narrow) claws, recurved on digit IV.
- Possible proximal webbing between digits II-III and III-IV.
- *Fuscinapeda* or *Wupus*?

# Track FF-4-1: Footprint Flats

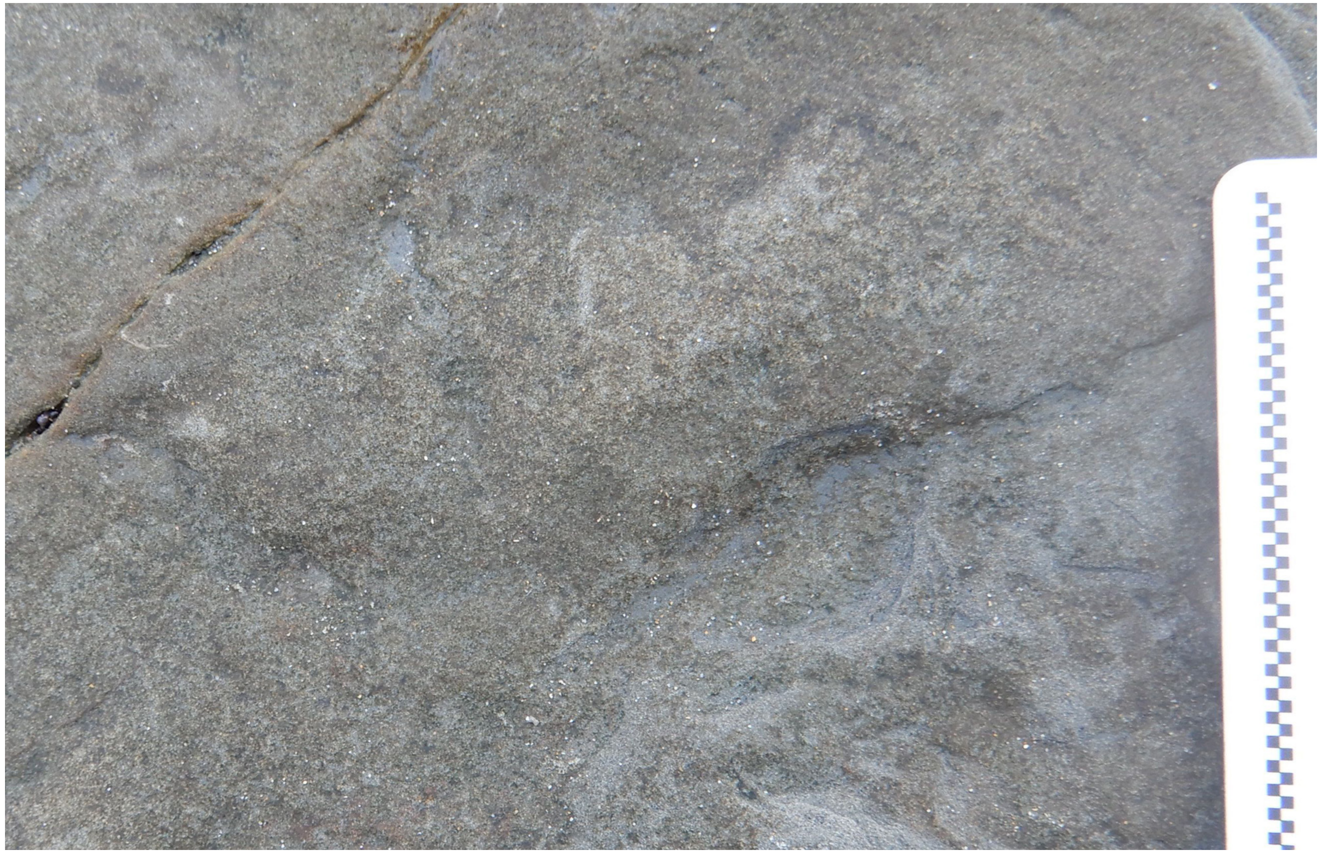

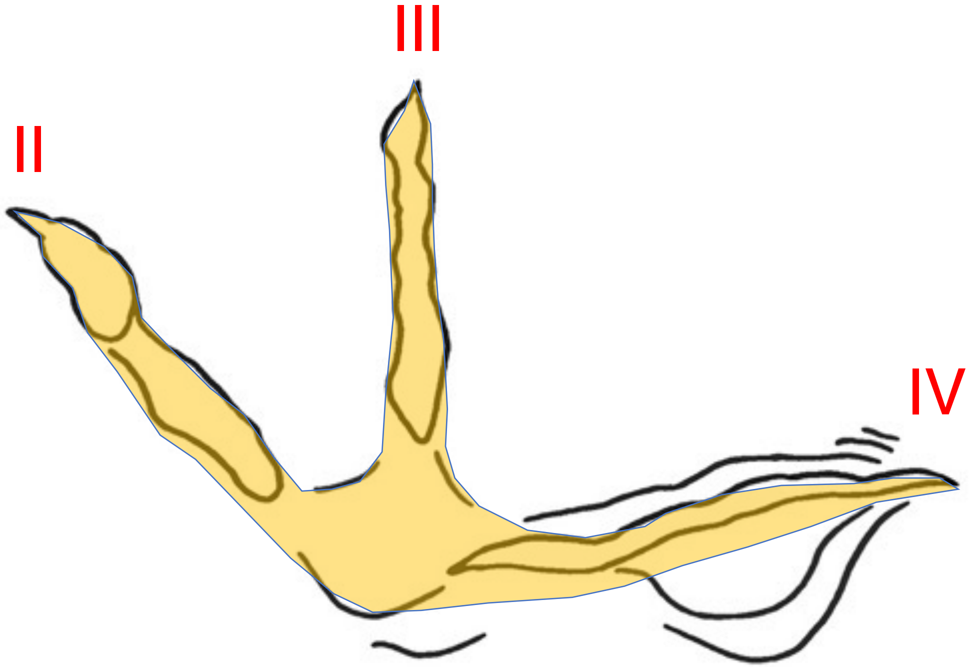


- Negative relief epichnion.
- Anisodactyl incumbent
- Digits definable by disruption of bedding
- Possible presence of phalangeal pad impressions.
- Sharp (narrow) claws, digits II and IV recurved.
- Possible proximal webbing between digits II-III and III-IV.
- *Wupus*?

# Track FF-4-2: Footprint Flats

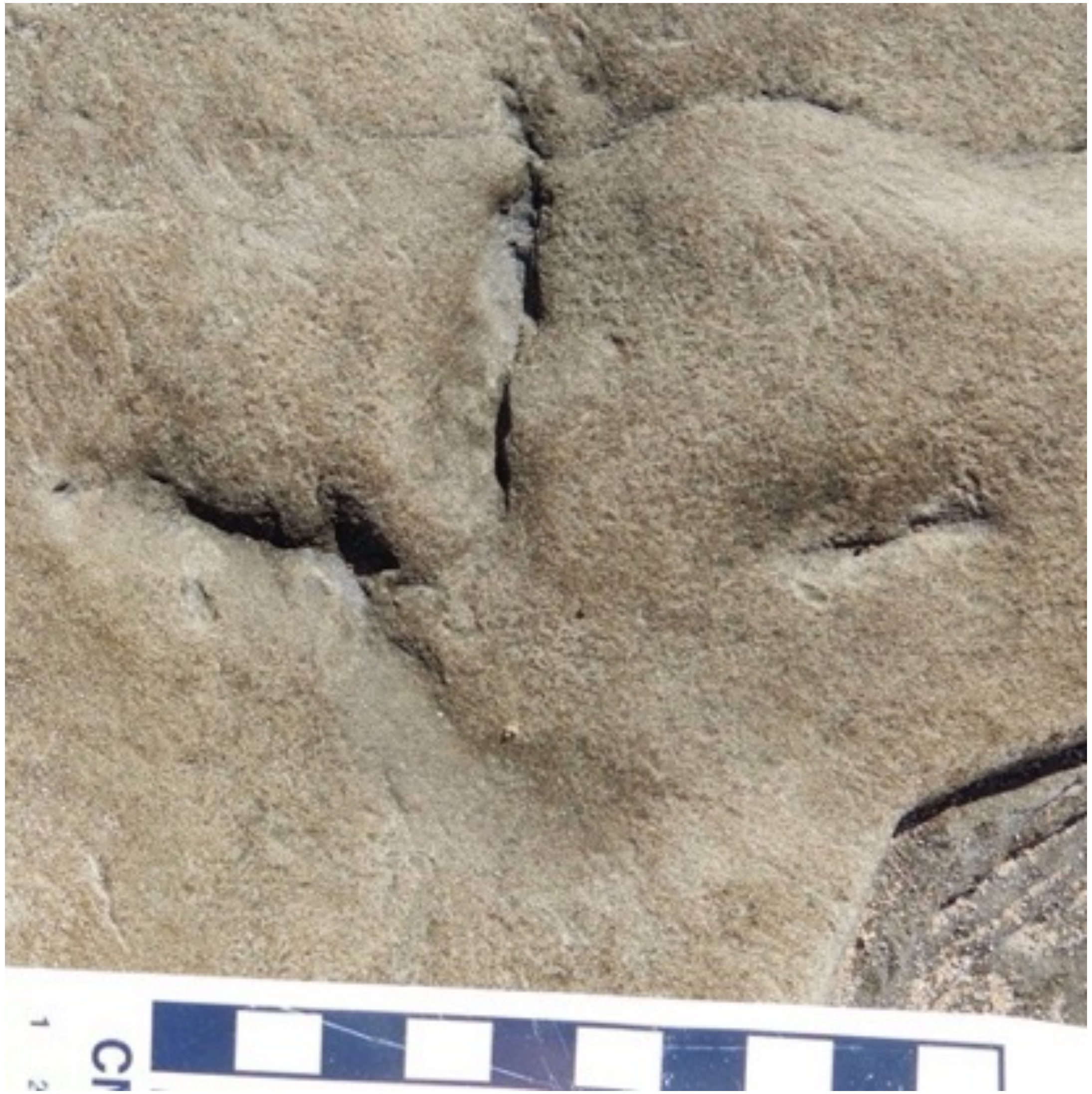

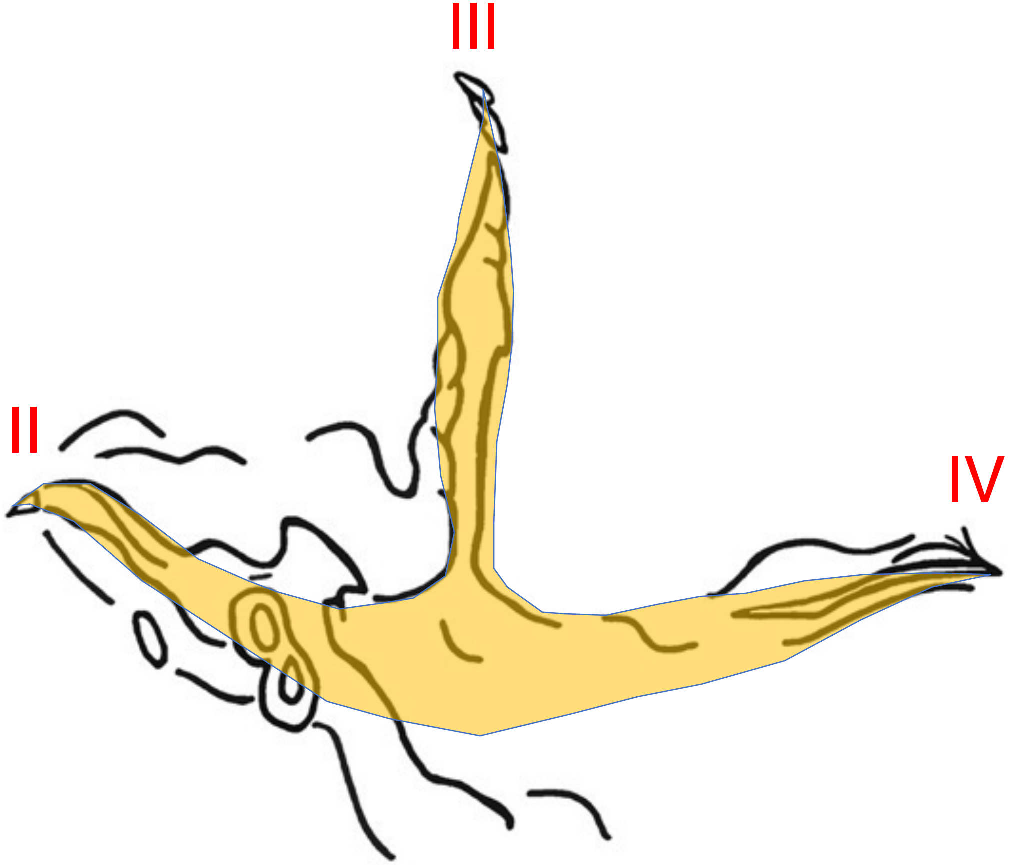


- Negative relief epichnion.
- Anisodactyl incumbent.
- Digits penetrate to underlying mudstone layer; lined invertebrate burrows associated with digits; metatarsal pad impression?
- Sharp claws, digits II and IV recurved.
- Possible proximal webbing between digits II-III and III-IV.
- *Avipeda?*

# Track FF-4-3: Footprint Flats

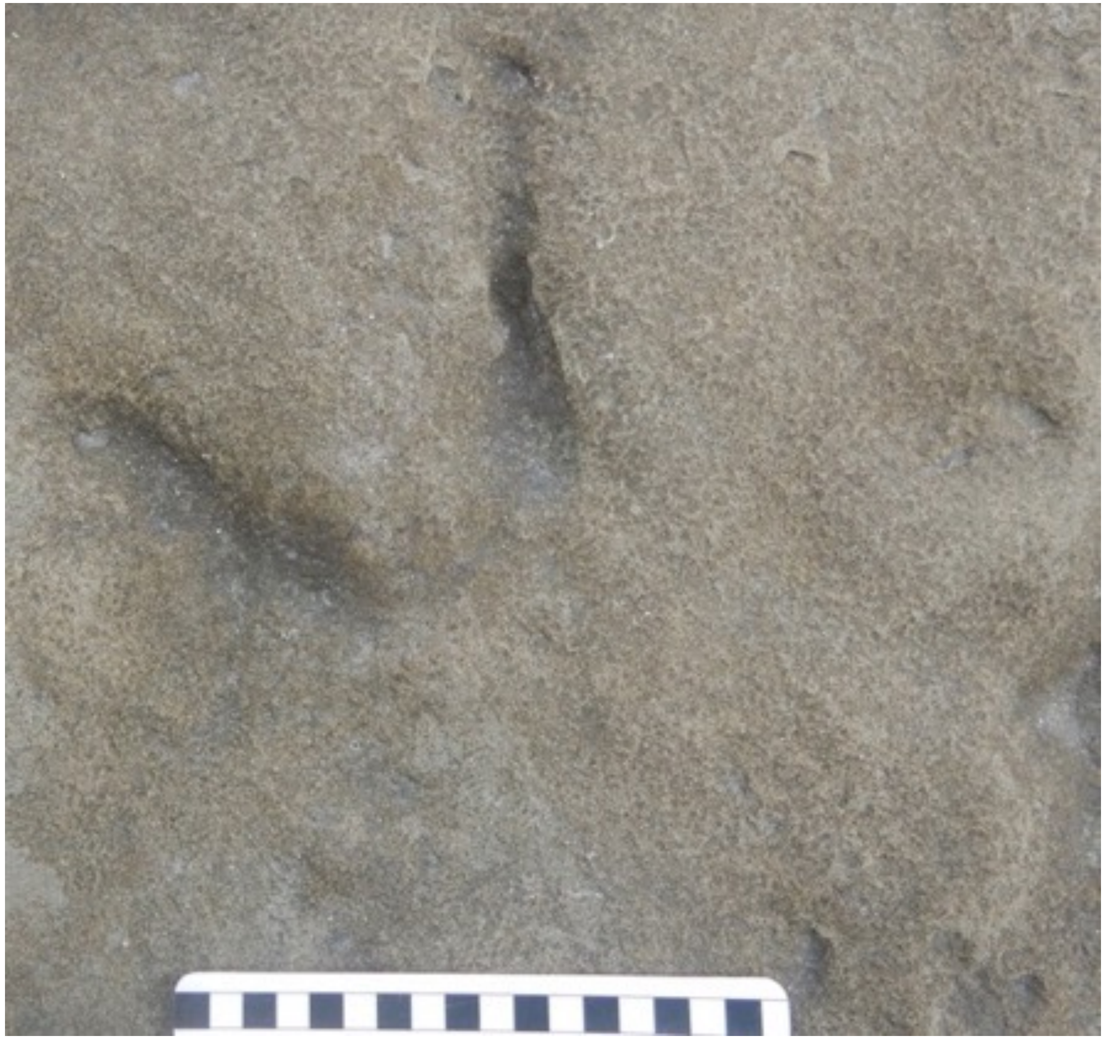

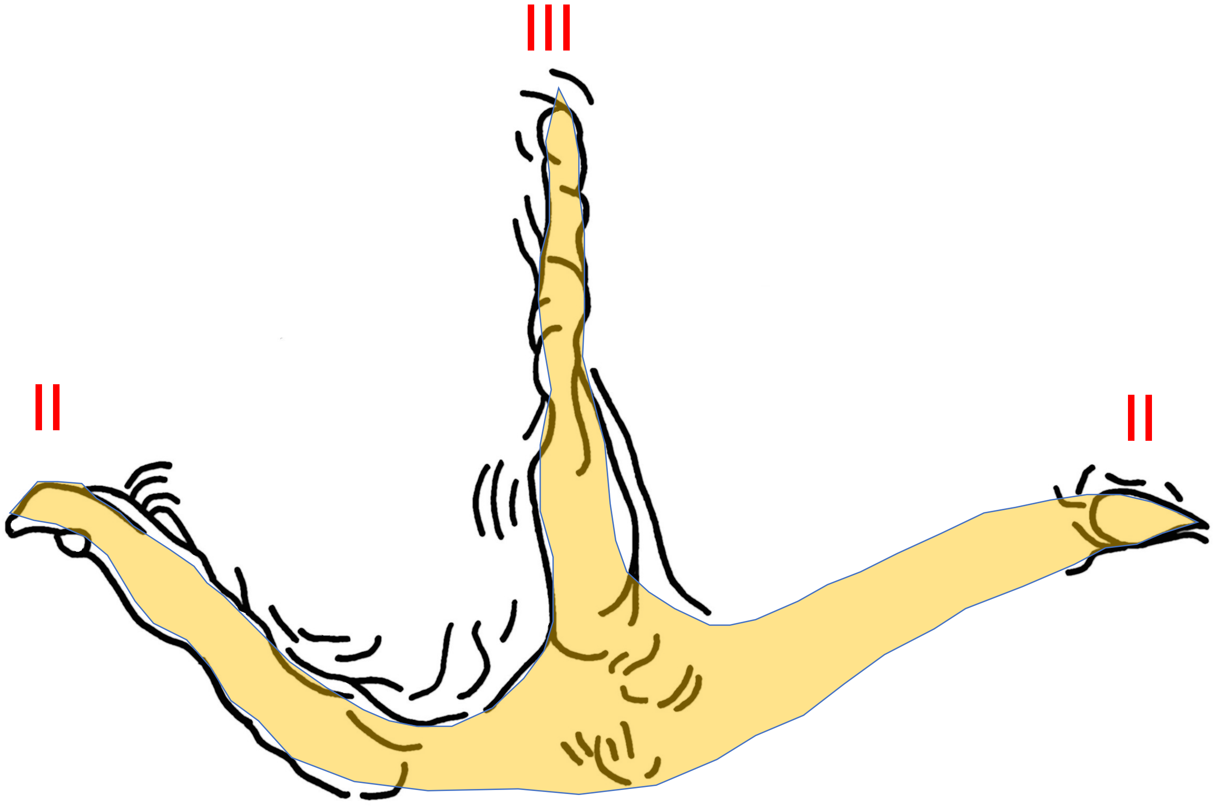


- Negative relief epichnion.
- Anisodactyl incumbent.
- Parts of digits penetrate to underlying mudstone
- Possible phalangeal pad impressions.
- Sharp (narrow) claws, recurved on digits II and IV.
- Possible proximal webbing between digits II-III.
- *Wupus*?

# Track FF-4-4: Footprint Flats

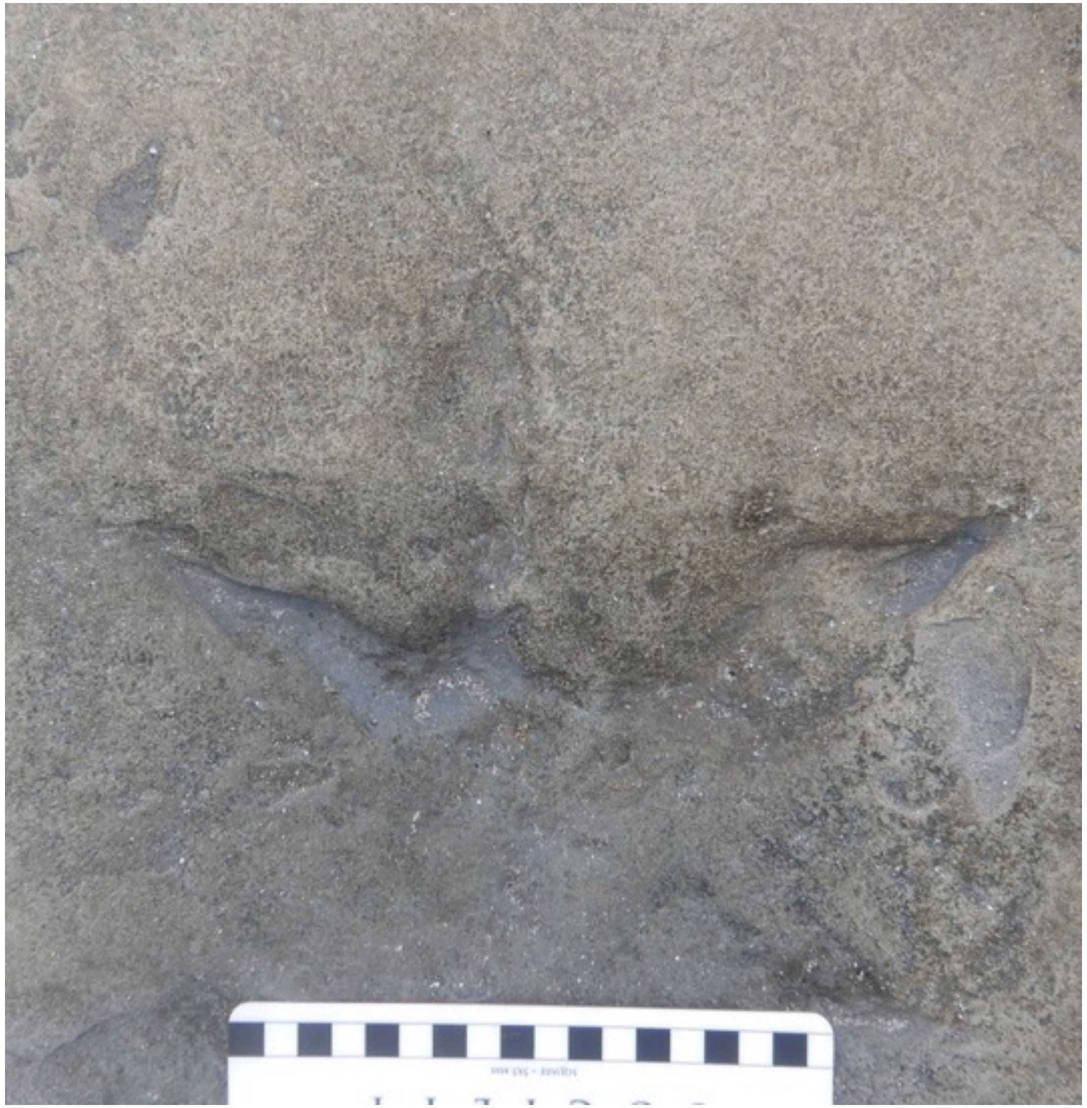

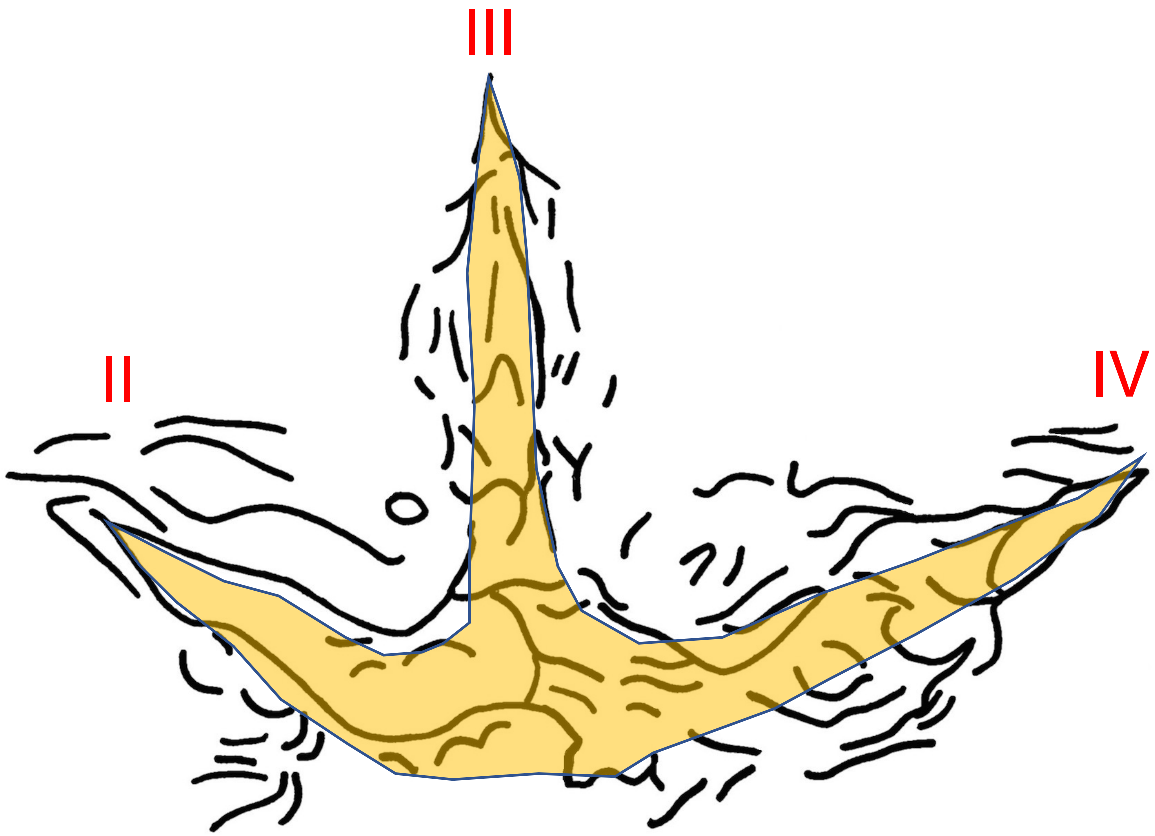


- Negative relief epichnion.
- Anisodactyl incumbent.
- Digits penetrated to underlying mudstone, co-occur with small invertebrate burrows.
- Sharp (narrow) claws, recurved on digits II and IV.
- Possible proximal webbing between digits II-III and III-IV.
- *Fuscinapeda?*

# Track FF-4-5: Footprint Flats

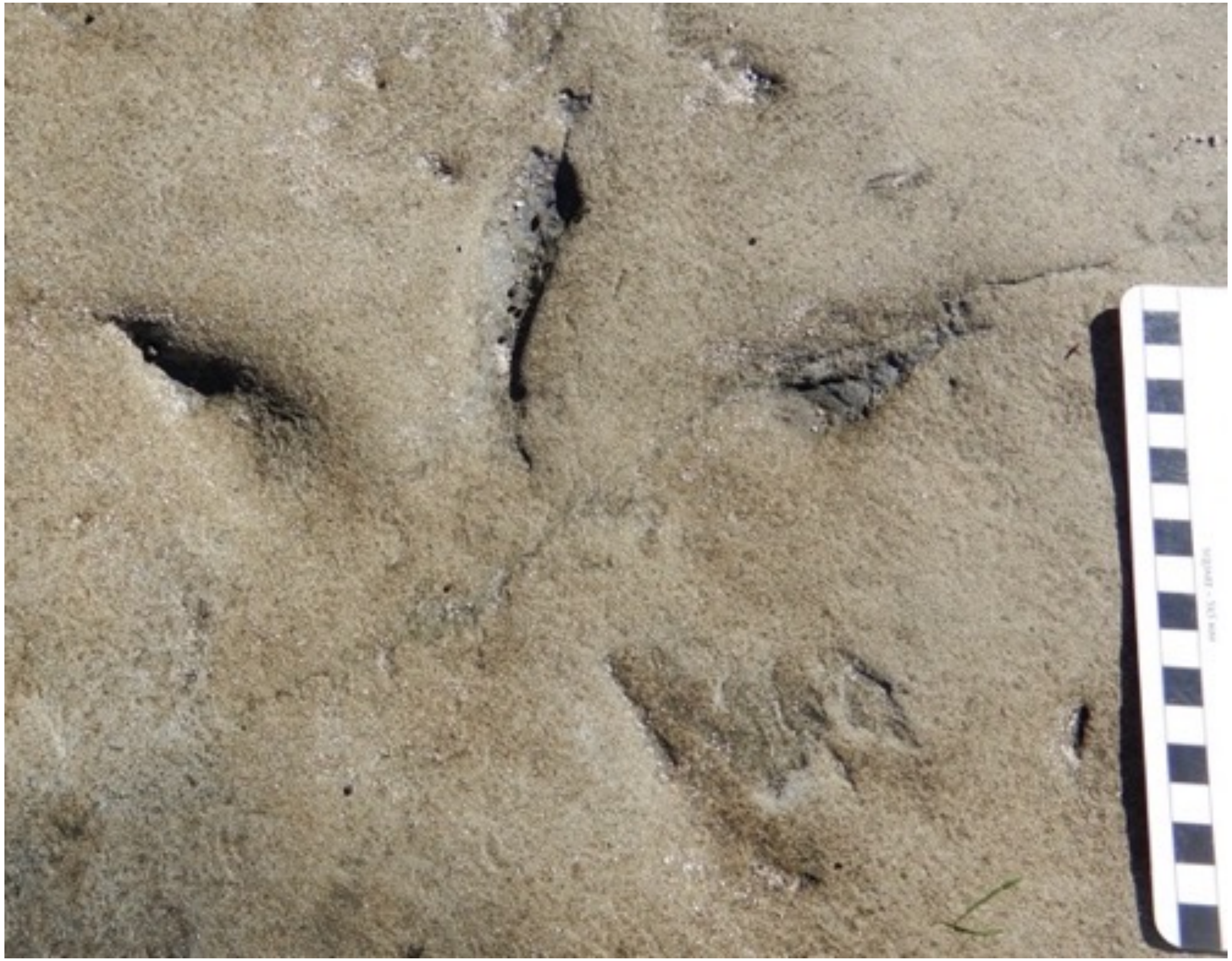

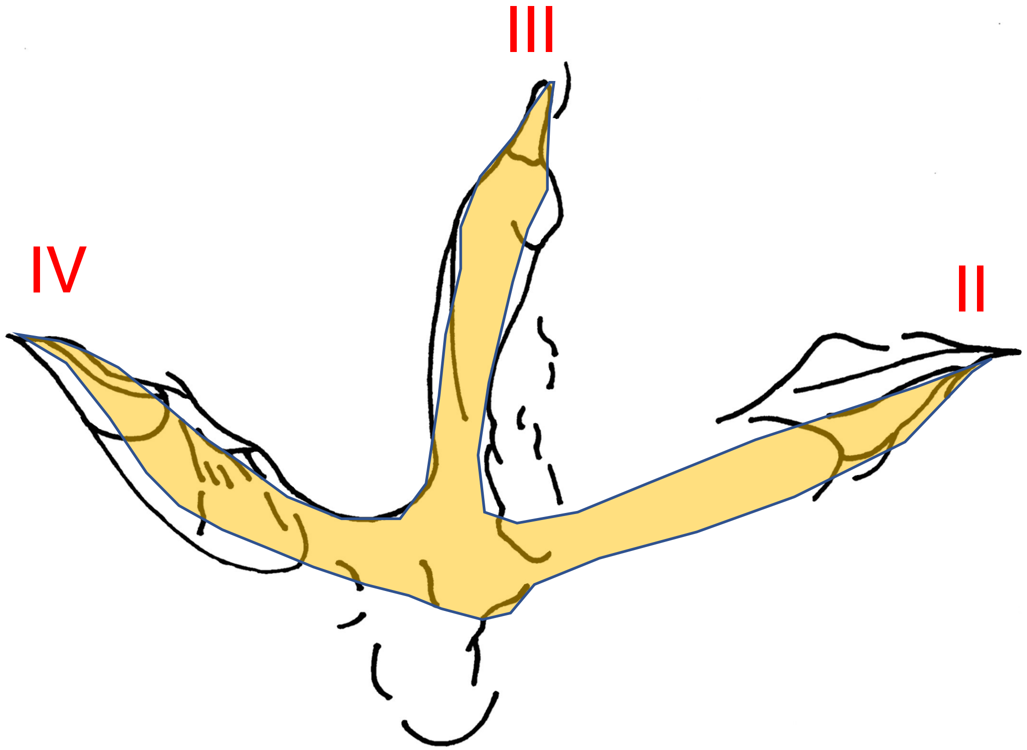


- Negative relief epichnion.
- Anisodactyl incumbent.
- Digits penetrated to underlying mudstone.
- Possible phalangeal and metatarsal pad impressions.
- Sharp (narrow) claws, recurved on digits II and IV.
- Possible proximal webbing between digits II-III and III-IV.
- *Fuscinapeda* or *Wupus*?

# Track FF-4-6: Footprint Flats

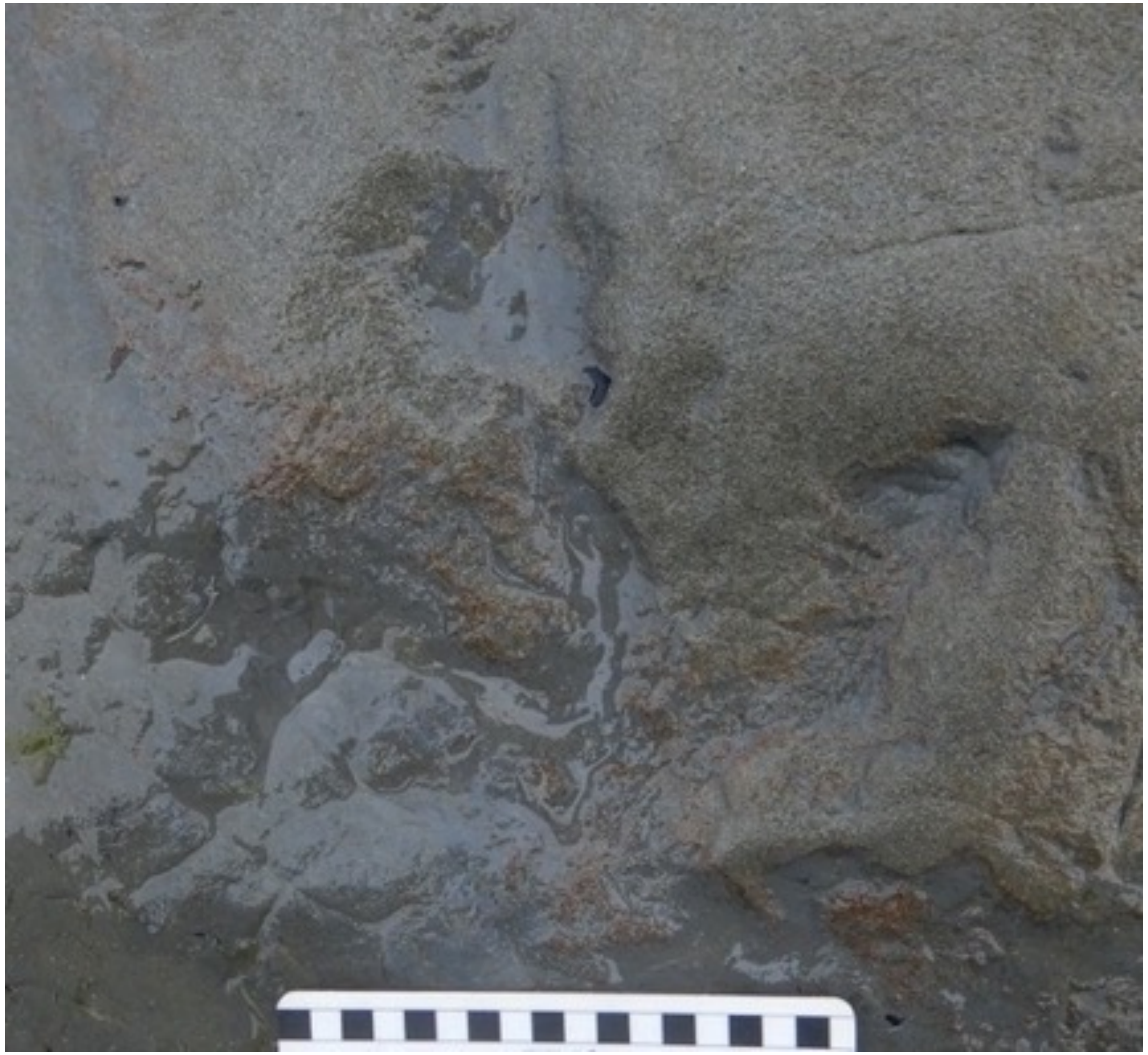

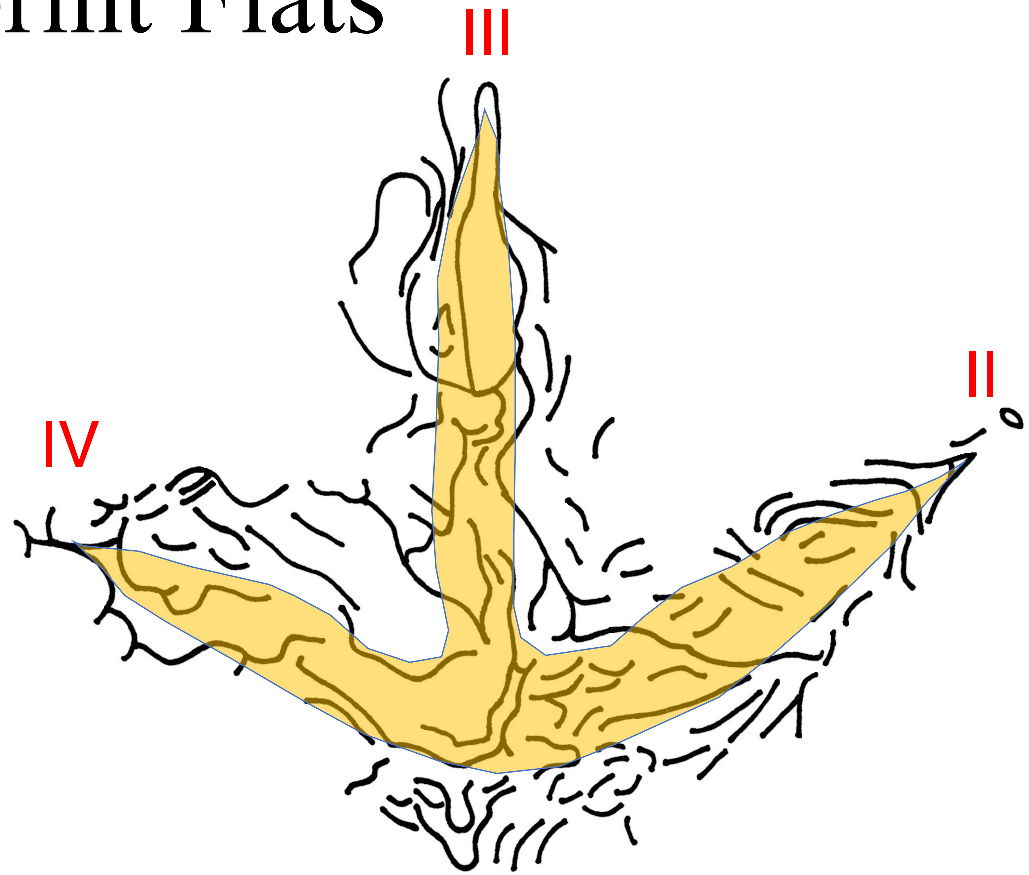


- Negative relief epichnion.
- Anisodactyl incumbent.
- Digits penetrated to underlying mudstone.
- Digit III "bent" by saturated sediments?
- Claws poorly defined, one digit (IV?) only partially preserved.
- No webbing apparent.
- Ichnogenus not determined.

# Track FF-5A-1 (cast): Footprint Flats

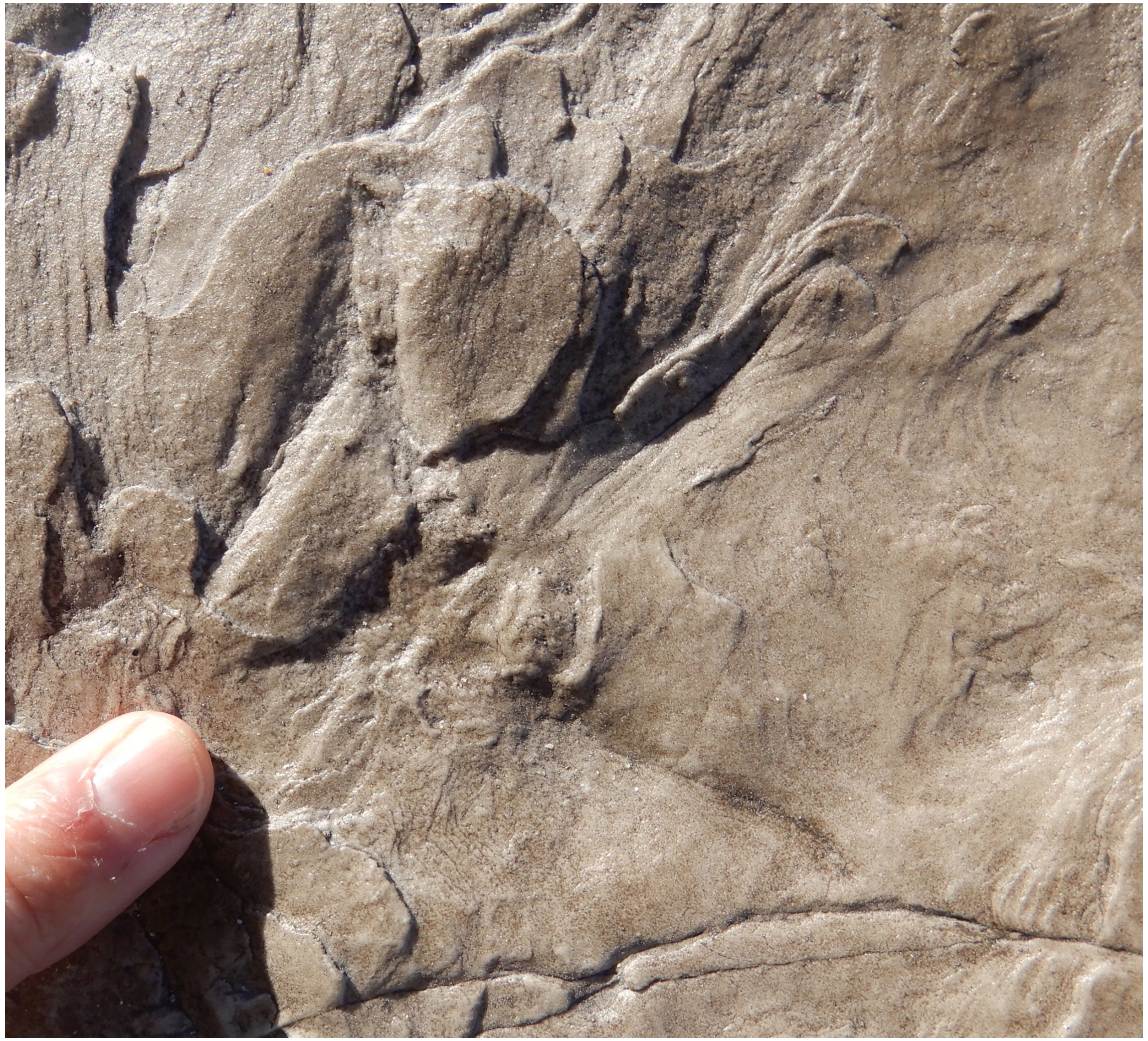

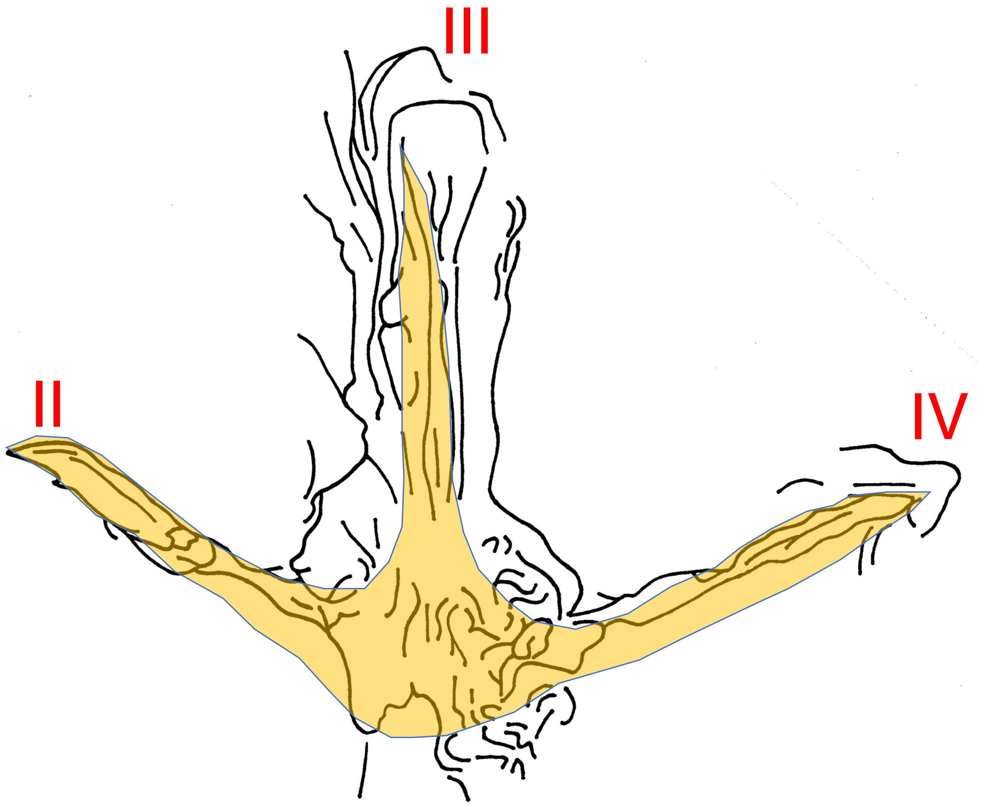


- Negative relief epichnion.
- Anisodactyl incumbent.
- Digits directly associated with sedimentary ridges (sudden stop?).
- Sharp (narrow) claws, recurved on digits II and IV, possible ungual drag in middle of digit III imprint.
- Possible webbing between digits II-III and III-IV.
- *Aquatilavipes?*

# Track FF-5A-2 (cast): Footprint Flats

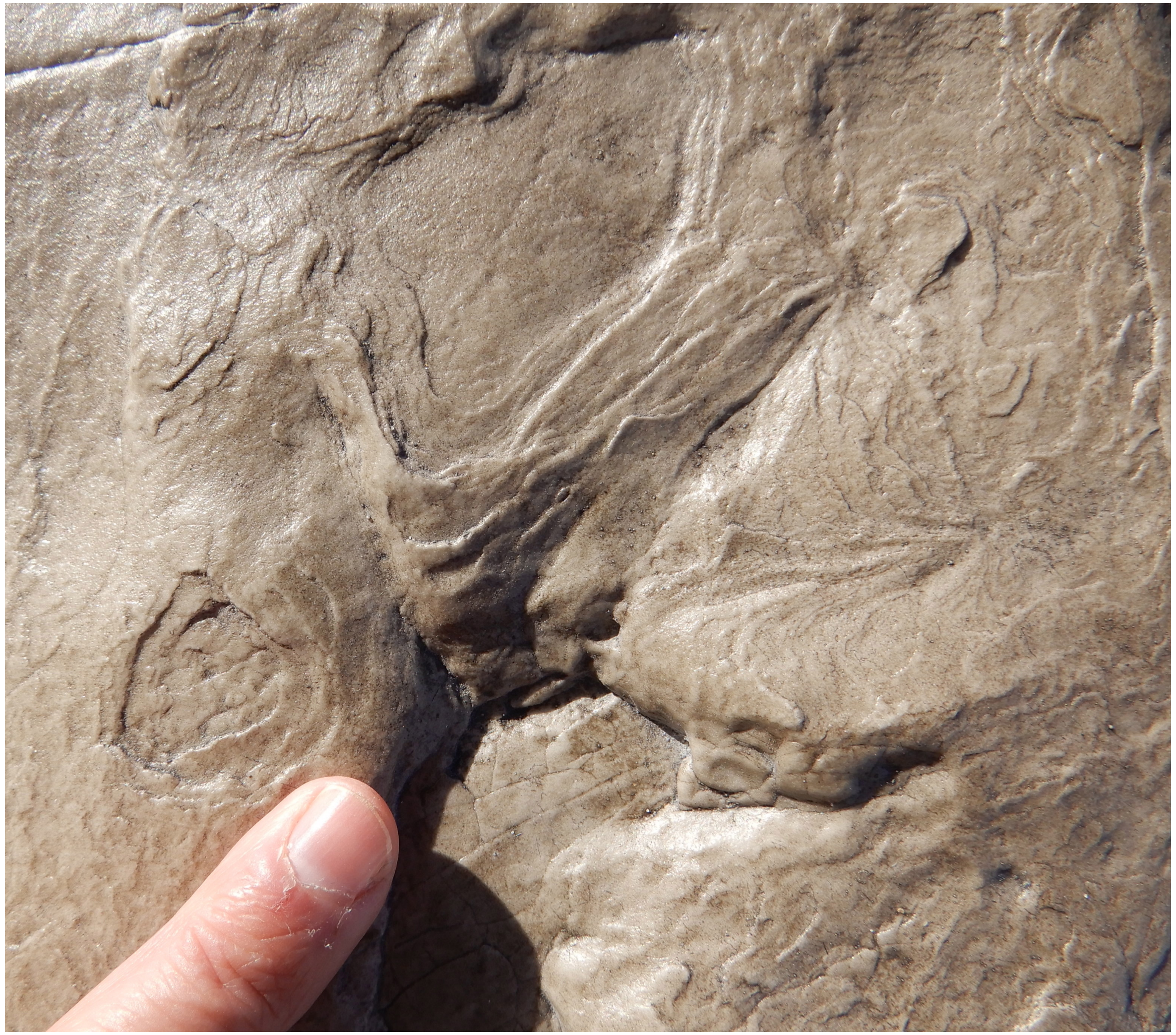

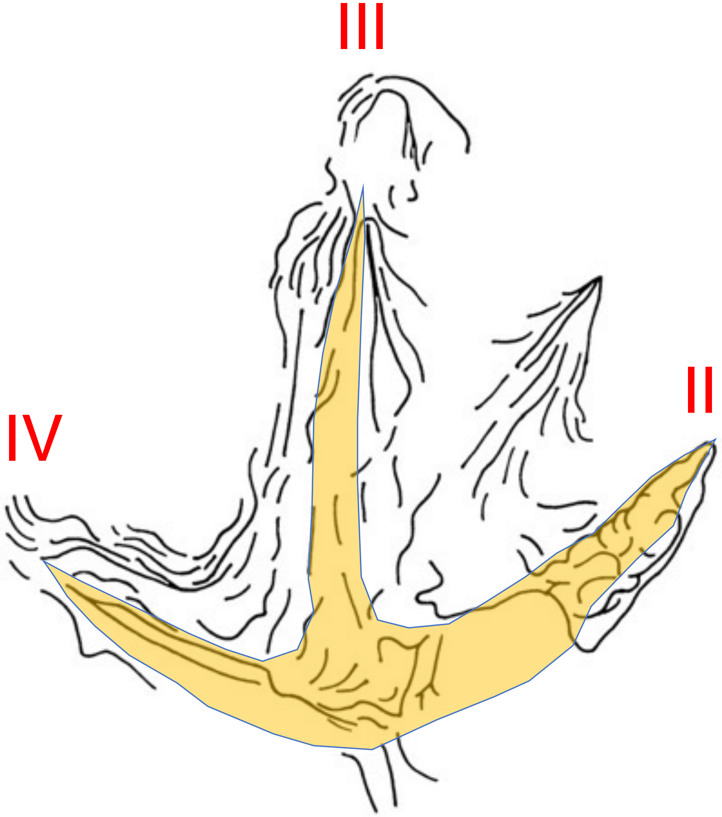


- Negative relief epichnion.
- Anisodactyl incumbent.
- 1-2 digits from another (probably underlying) track, sedimentary structures around digits III and IV (from foot movement?).
- Sharp (narrow) claws, with possible ungual drag in center of digit III.
- Possible proximal webbing between digits II-III and III-IV.
- *Avipeda?*

# Track FF-5A-3 (bottom): Footprint Flats

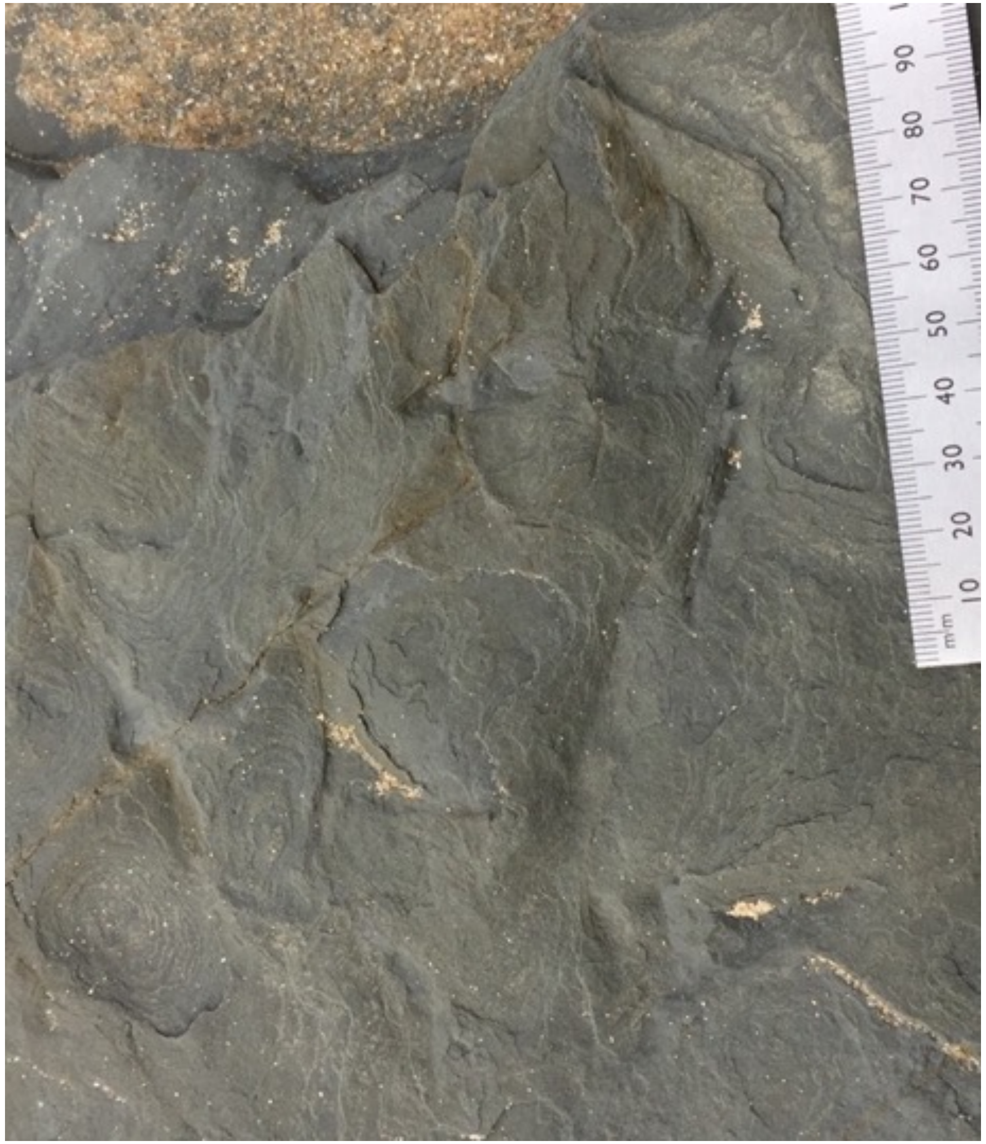


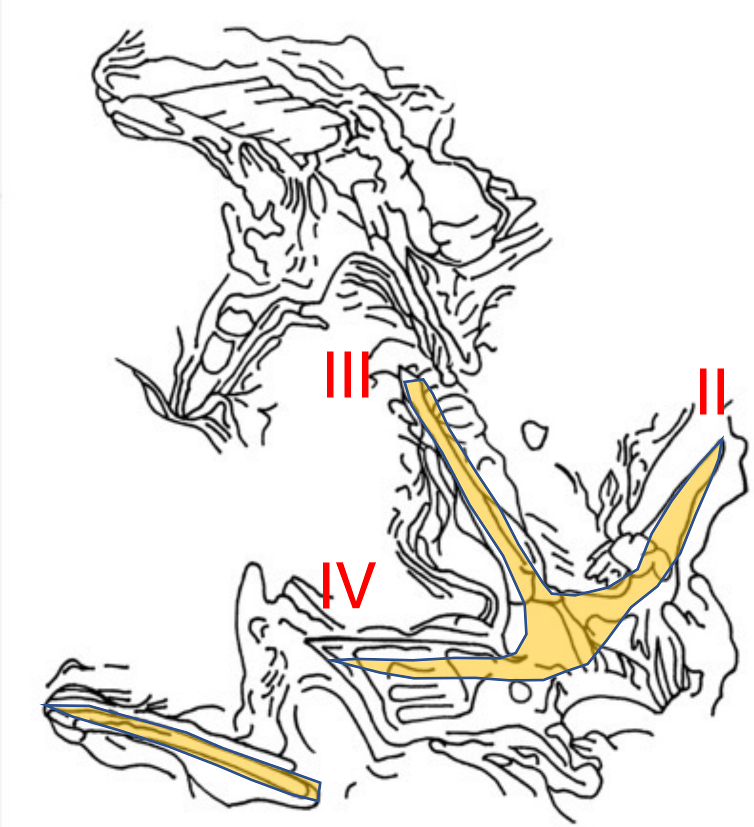


- Negative relief epichnion.
- Anisodactyl incumbent.
- Digit imprints with sediment deformation (sudden stop?), single digit from another track next to digit IV.
- Sharp (narrow) claws, one recurved.
- Possible proximal webbing between digits II-III and III-IV.
- *Fuscinapeda?*

# Track FF-5A-4 (right): Footprint Flats

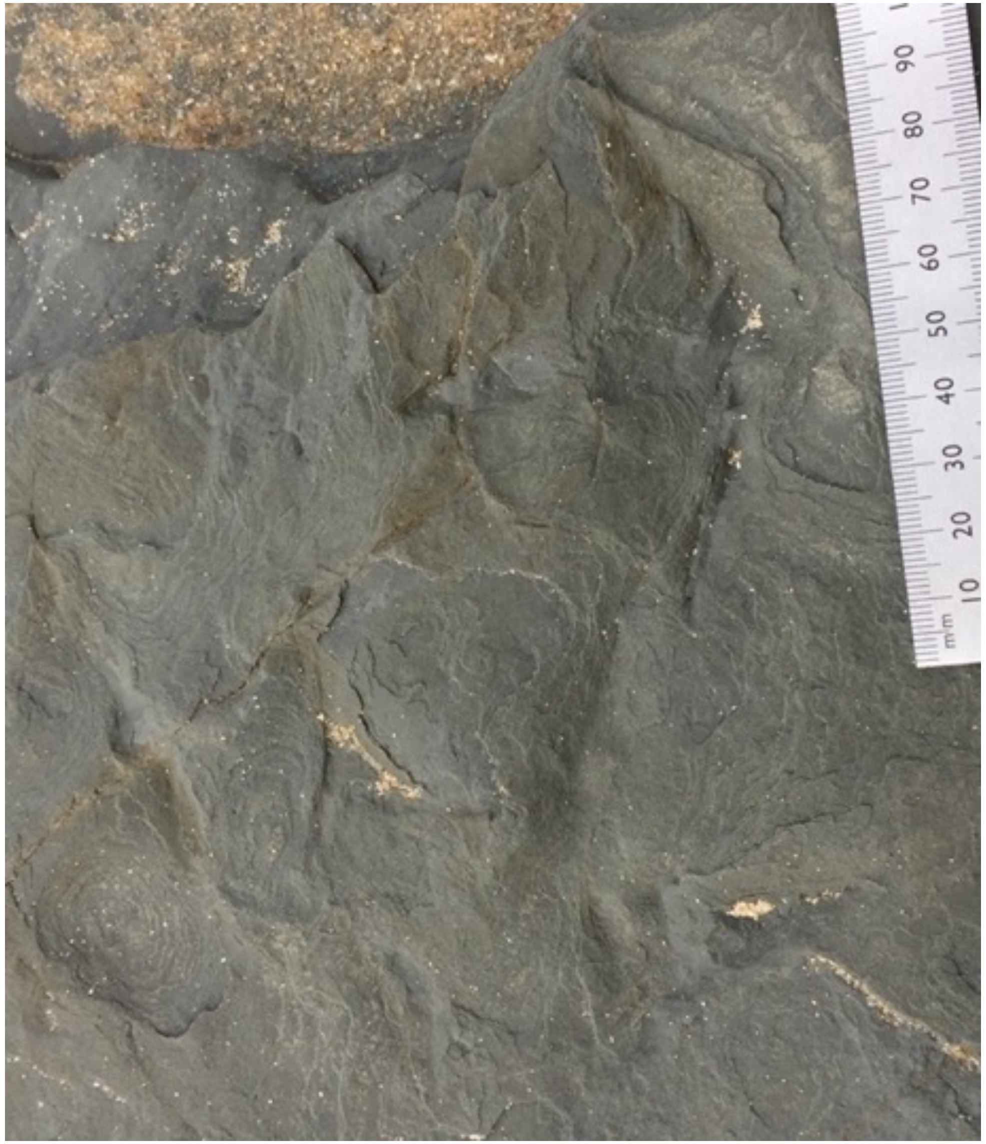


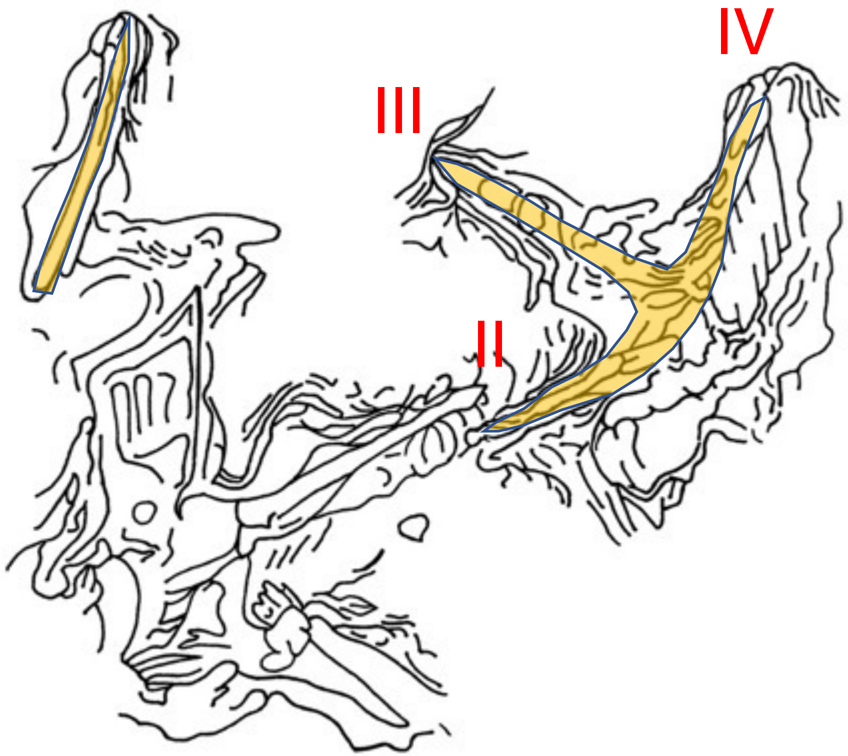


- Negative relief epichnion.
- Anisodactyl incumbent.
- Digit imprints with sediment deformation (sudden stop?).
- Sharp (narrow) claws.
- Possible proximal webbing between digits II-III and III-IV.
- *Fuscinapeda?*

# Track FF-5A-5: Footprint Flats

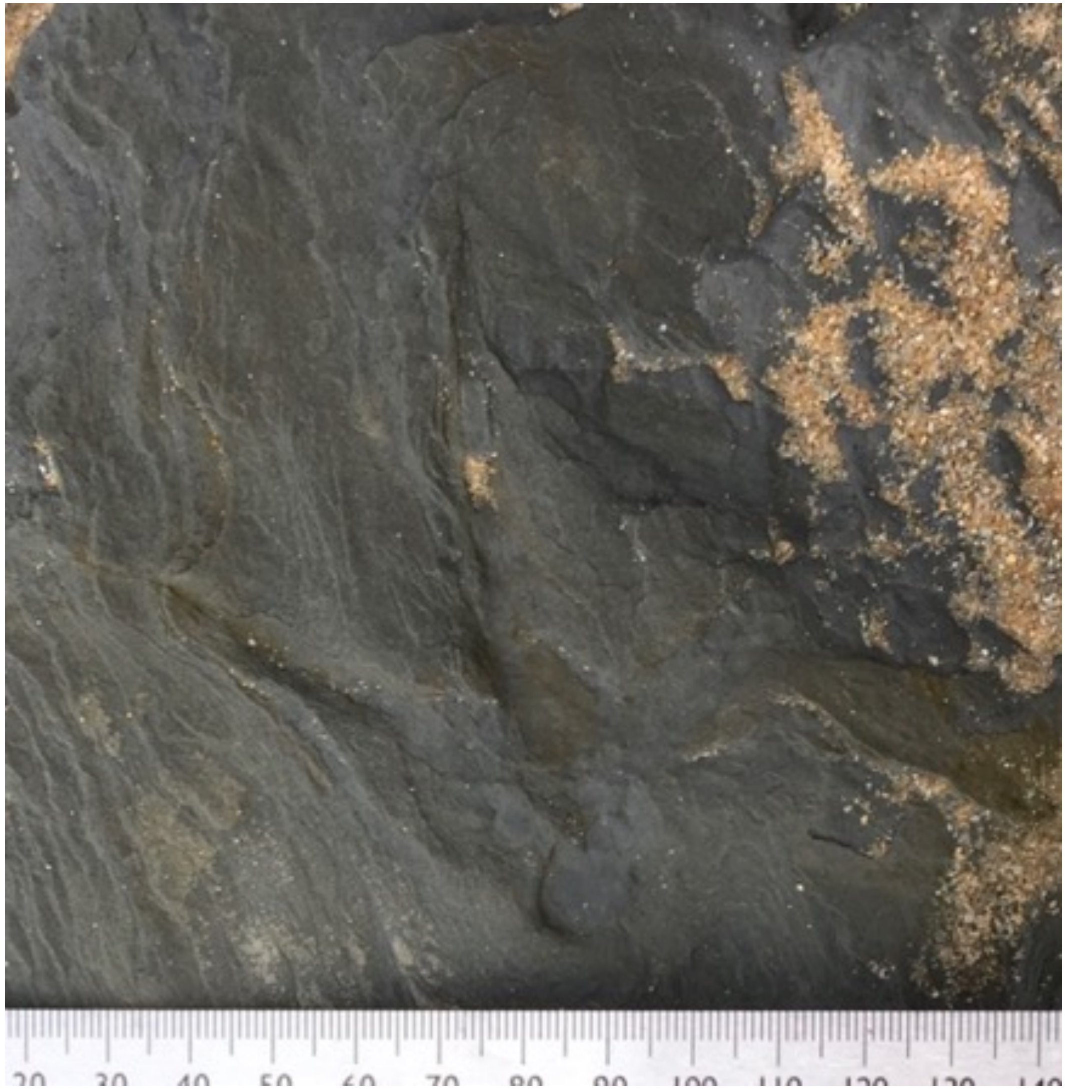

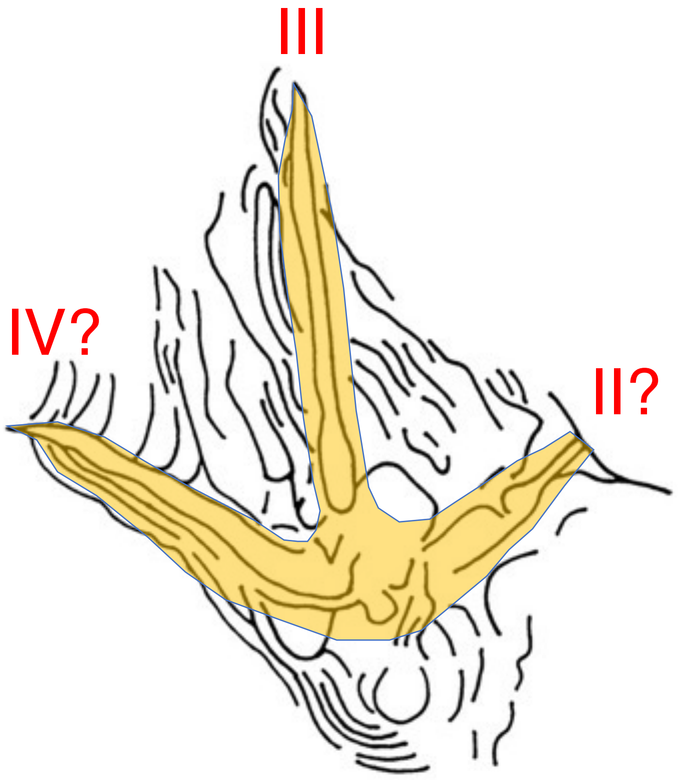


- Negative relief epichnion.
- Anisodactyl incumbent.
- Digit imprints with sediment deformation one digit (II?) incomplete (weathered from rock).
- Sharp (narrow) claws, one (digit IV?) recurved.
- No webbing evident.
- *Limiavipes?*

# Track FF-5B-1: Footprint Flats

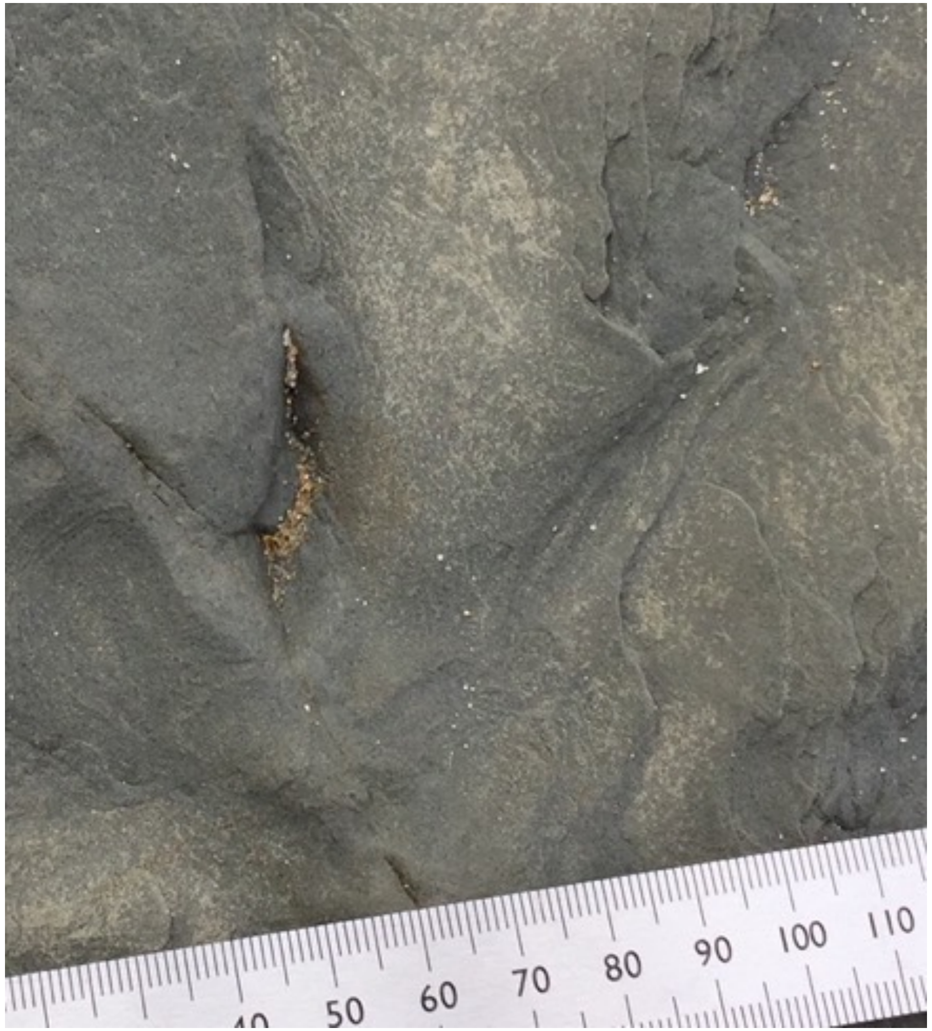


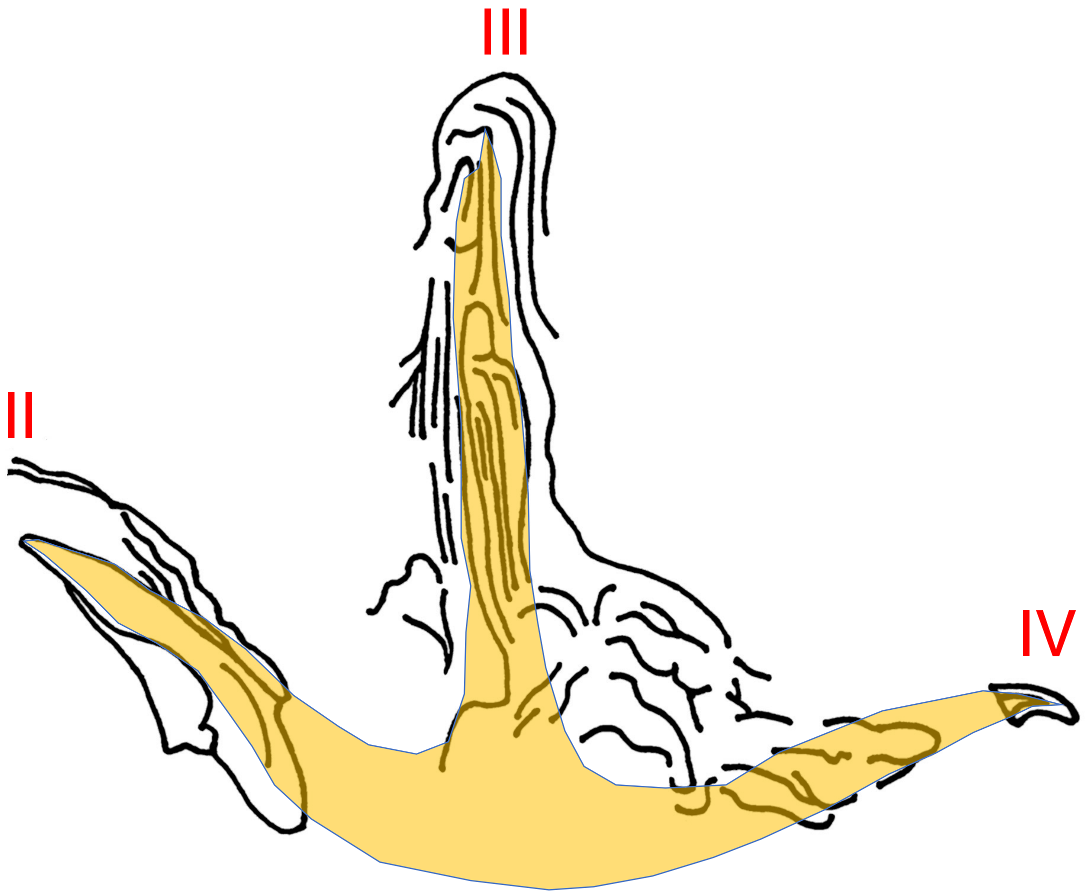


- Negative relief epichnion
- Anisodactyl incumbent
- Digit imprints with sediment deformation
- Sharp claws, recurved on digits II and IV.
- No webbing evident.
- *Wupus*?

# Track FF-5B-1 (cast): Footprint Flats

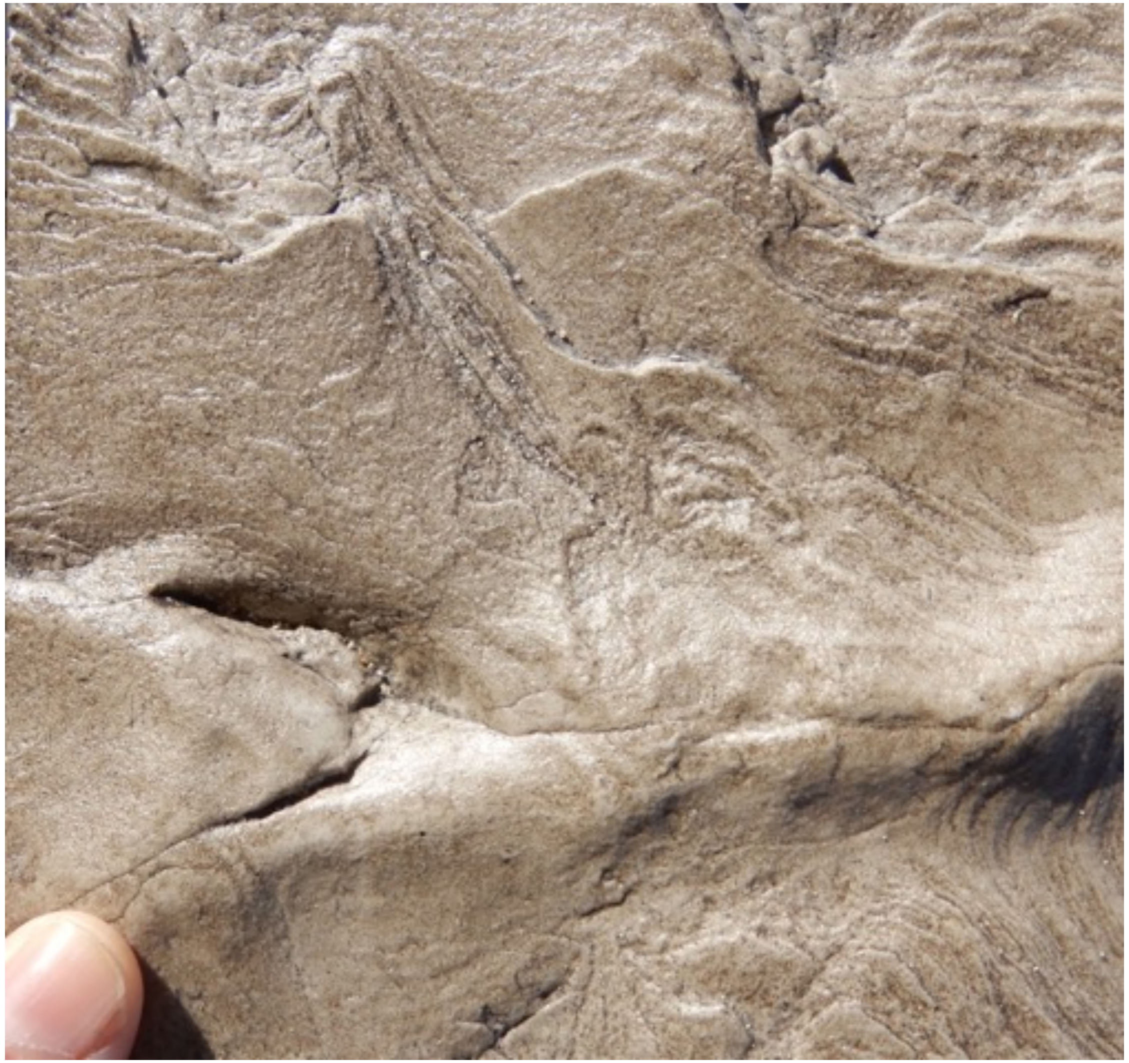

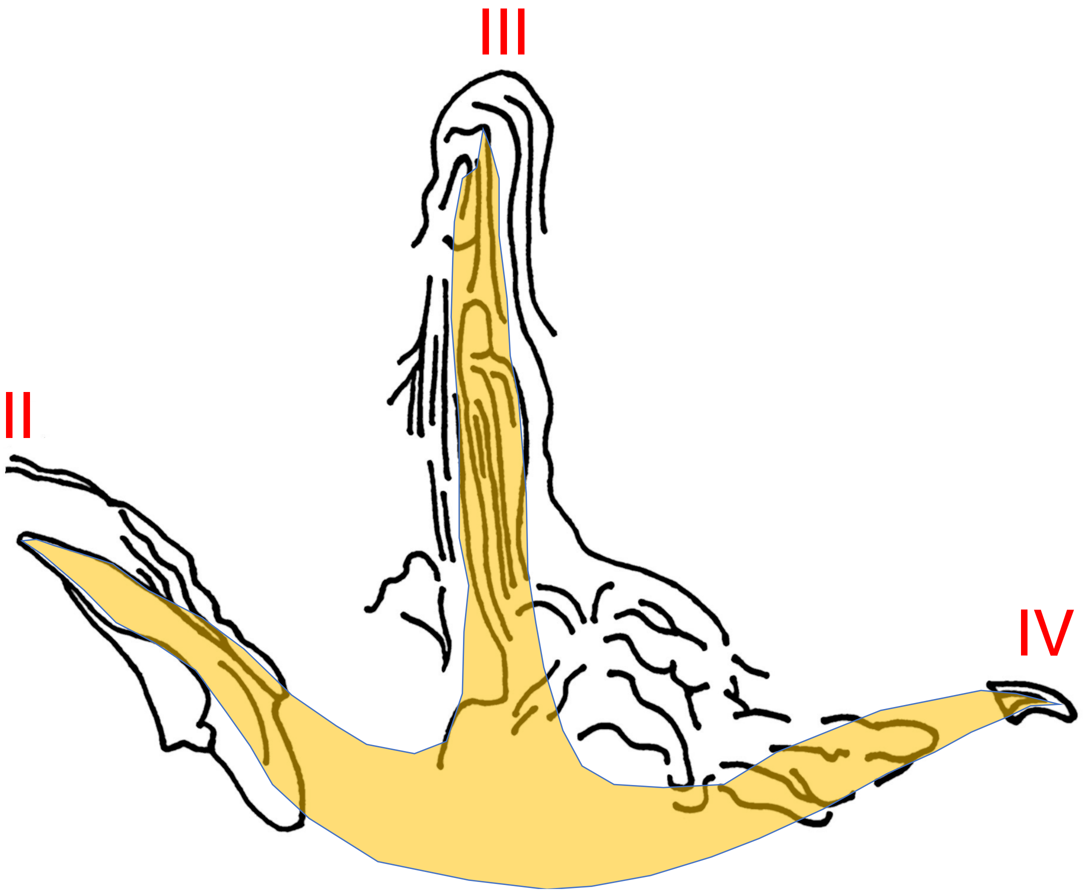


- Negative relief epichnion
- Anisodactyl incumbent
- Digit imprints with sediment deformation
- Sharp claws, recurved on digits II and IV.
- No webbing evident.
- *Wupus*?

# Track FF-5B-2: Footprint Flats

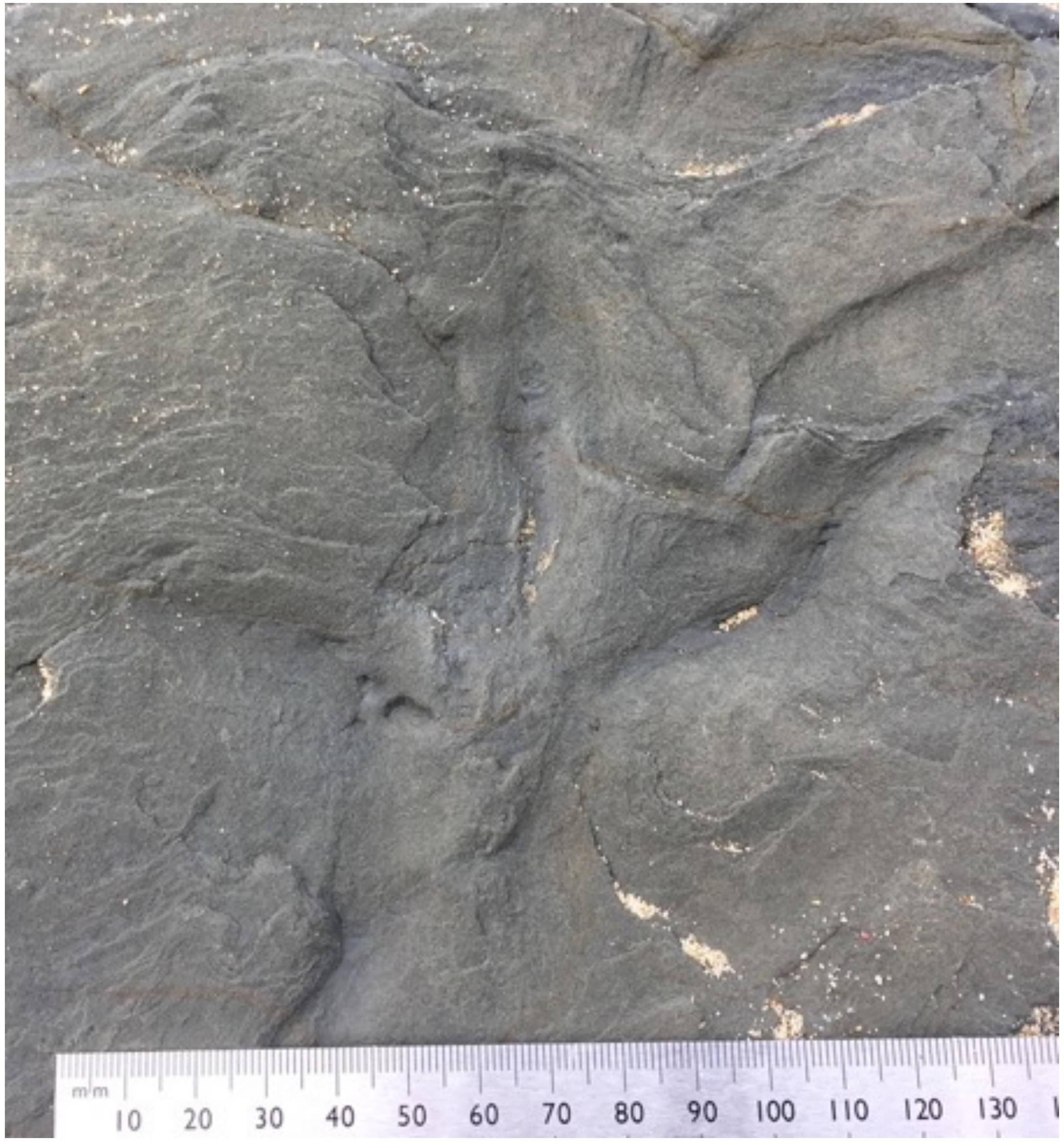


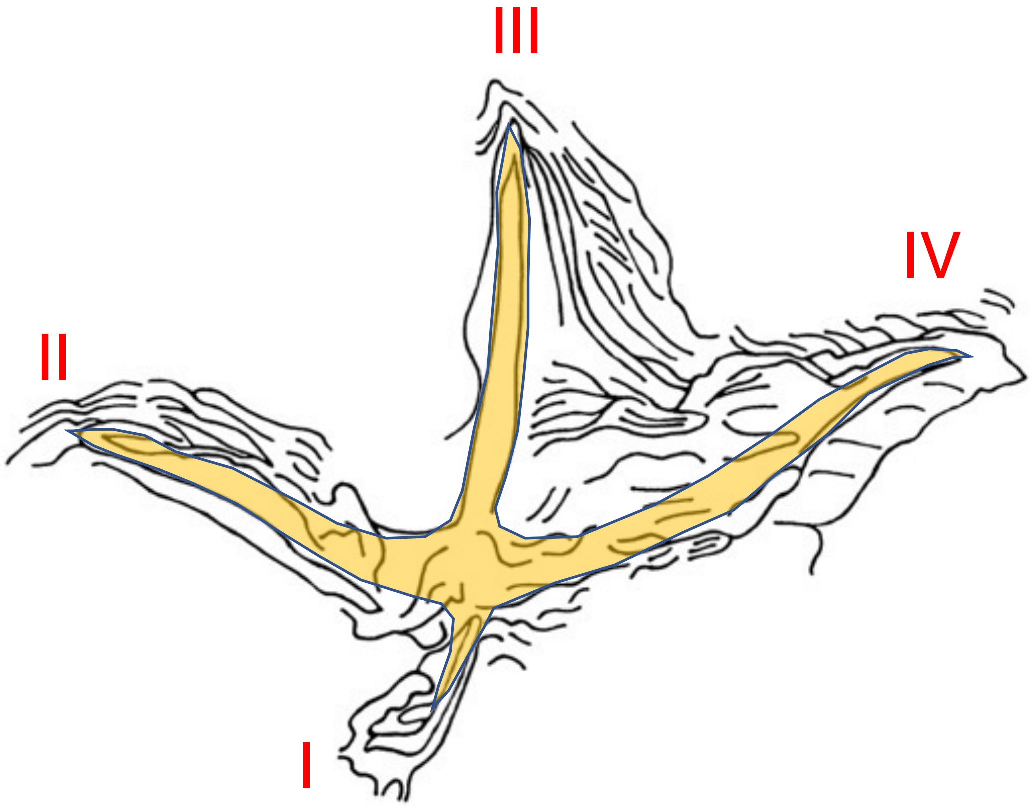


- Negative relief epichnion.
- Anisodactyl.
- Digit imprints with sediment deformation (sudden stop)?
- Sharp claws, recurved on digits II and IV.
- Possible proximal webbing between digits II-III and III-IV.
- *Hwangsanipes?*

# Track FF-5B-2 (cast): Footprint Flats

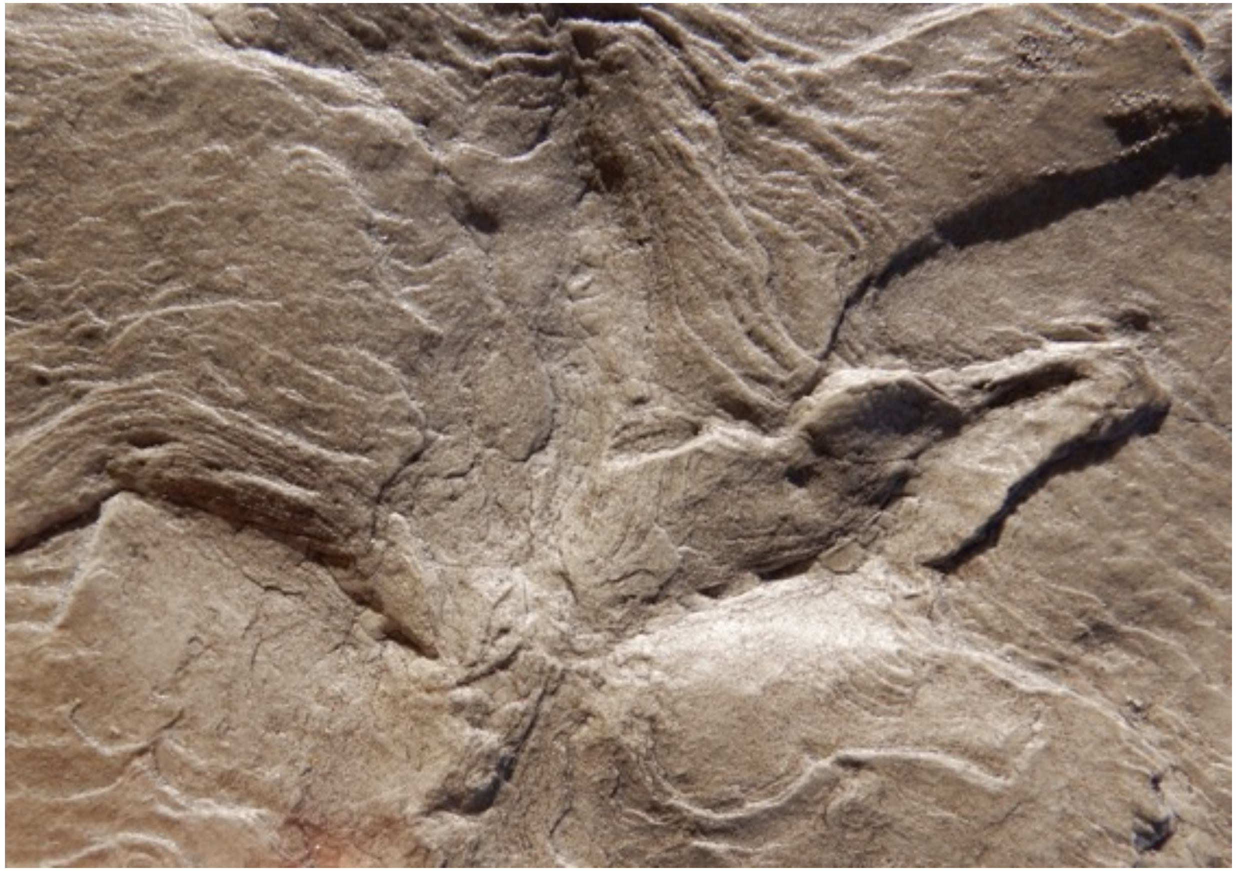

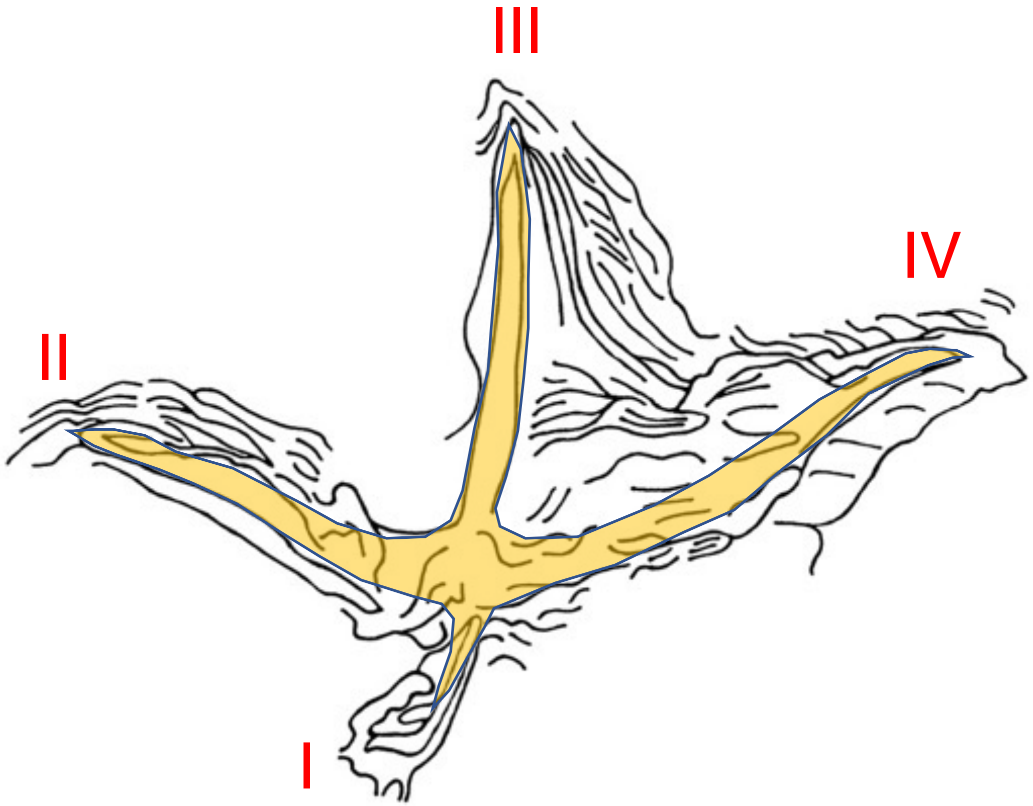


- Negative relief epichnion.
- Anisodactyl.
- Digit imprints with sediment deformation (sudden stop)?
- Sharp claws, recurved on digits II and IV.
- Possible proximal webbing between digits II-III and III-IV.
- *Hwangsanipes?*

# Track FF-5B-3: Footprint Flats

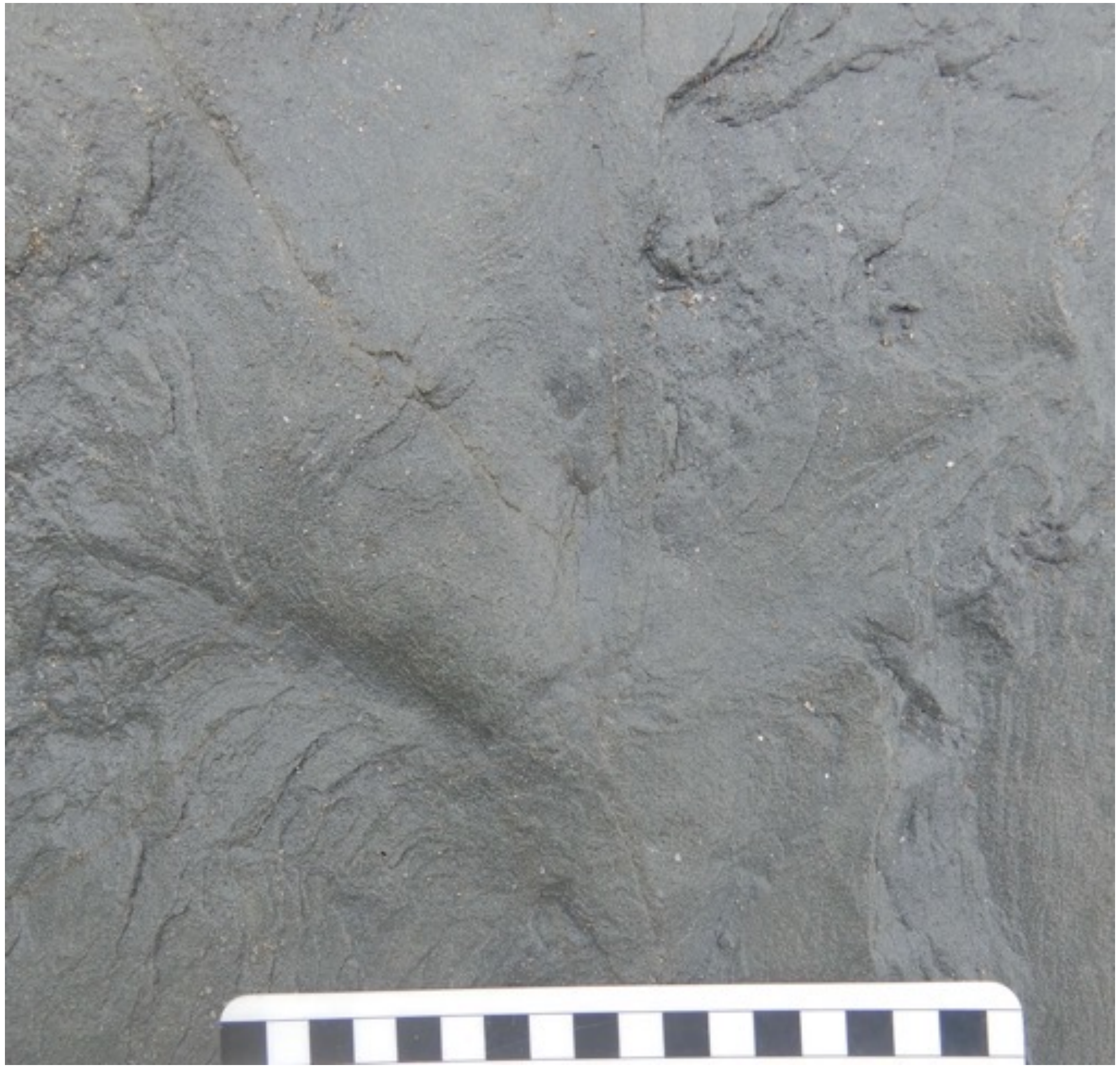

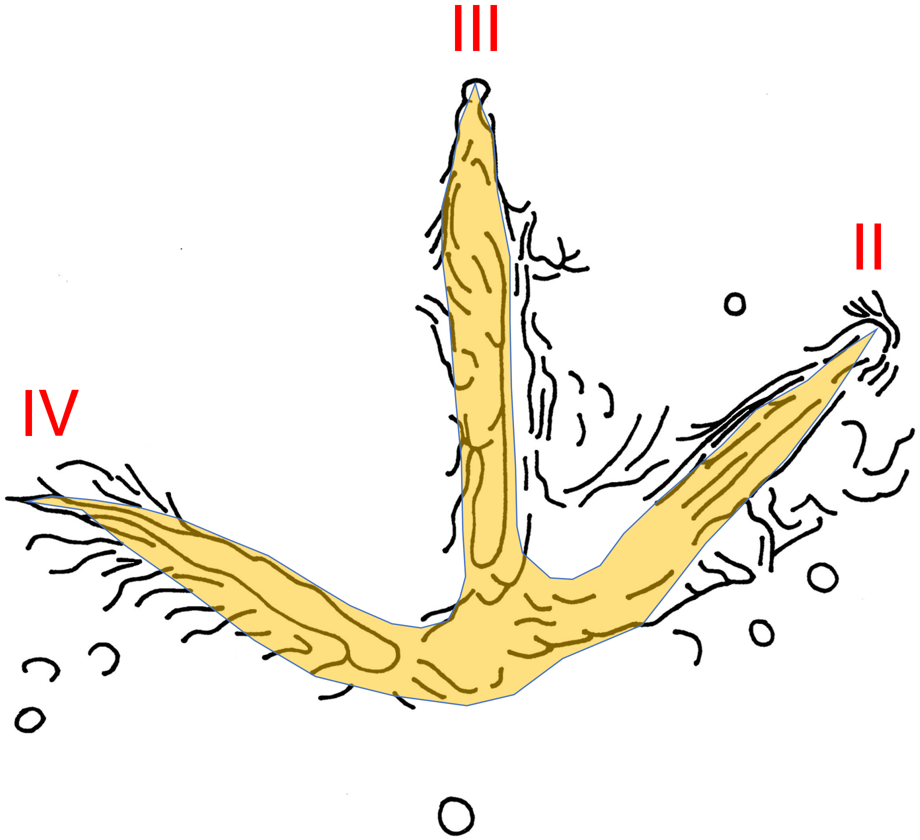


- Negative relief epichnion.
- Anisodactyl.
- Digit imprints with sediment deformation (sudden stop?); invertebrate burrows associated with track.
- Sharp (narrow) claws, recurved on digits II and IV.
- Possible proximal webbing between digits II-III and III-IV.
- *Wupus*?

# Track FF-5B-4: Footprint Flats

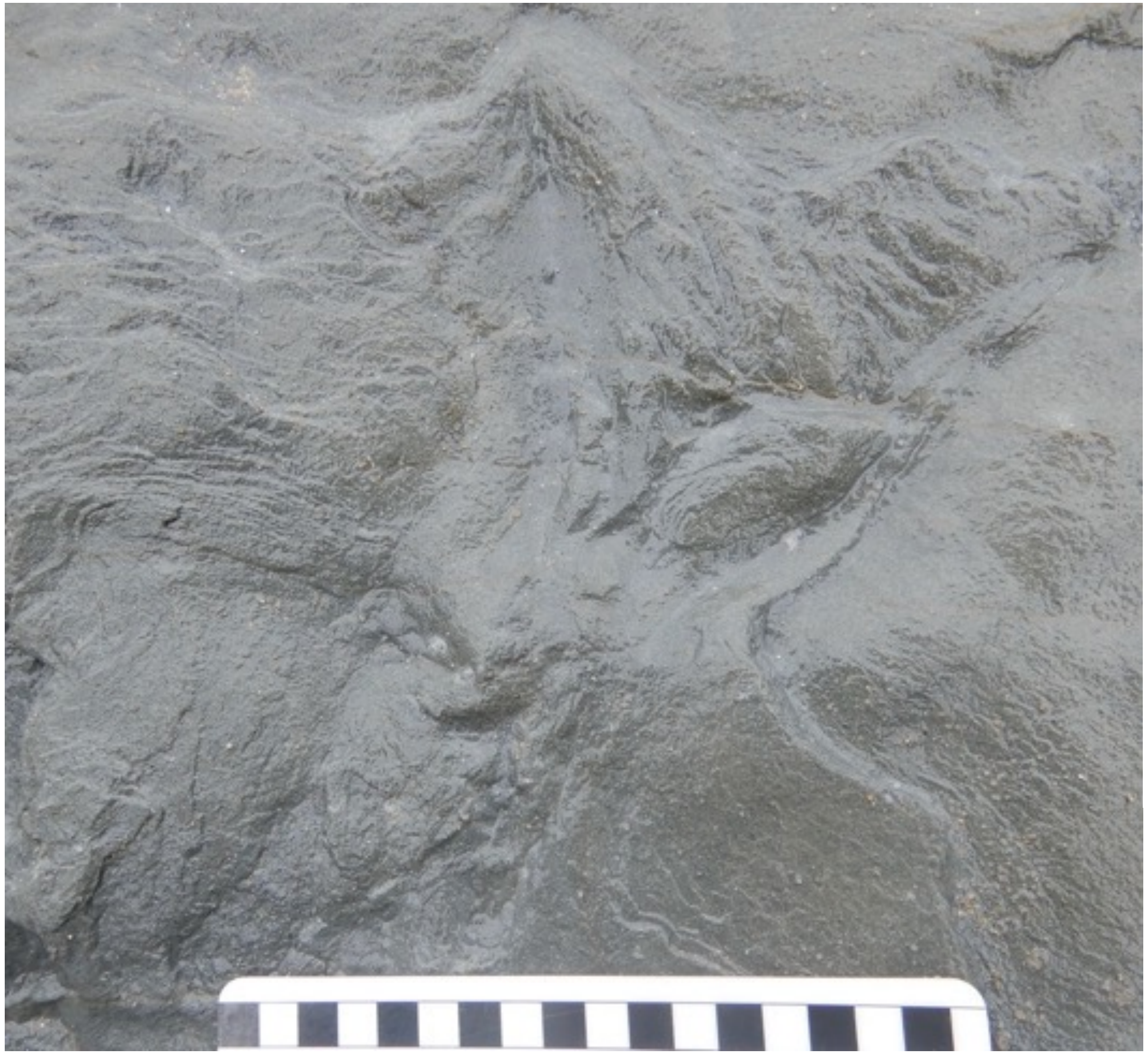

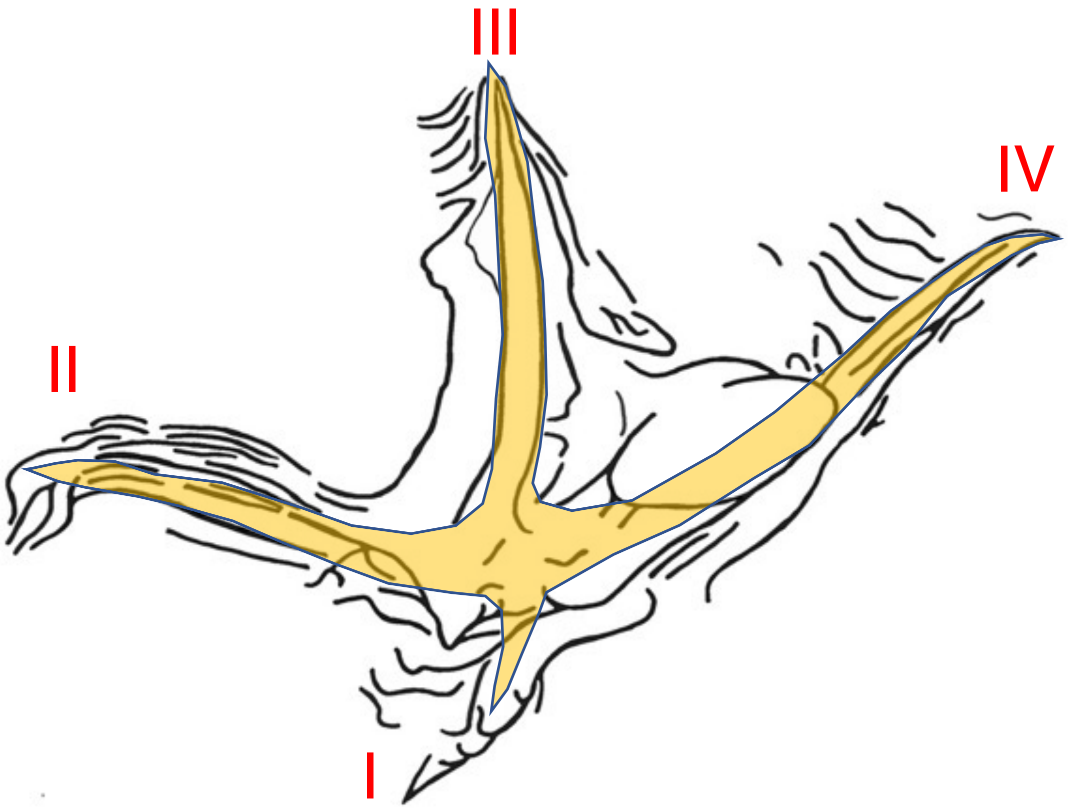


- Negative relief epichnion.
- Anisodactyl.
- Digit imprints with sediment deformation (sudden stop)?
- Sharp (narrow) claws, recurved on digits II and IV.
- Possible proximal webbing between digits II-III and III-IV.
- *Hwangsanipes?*

# Track FF-5B-5: Footprint Flats

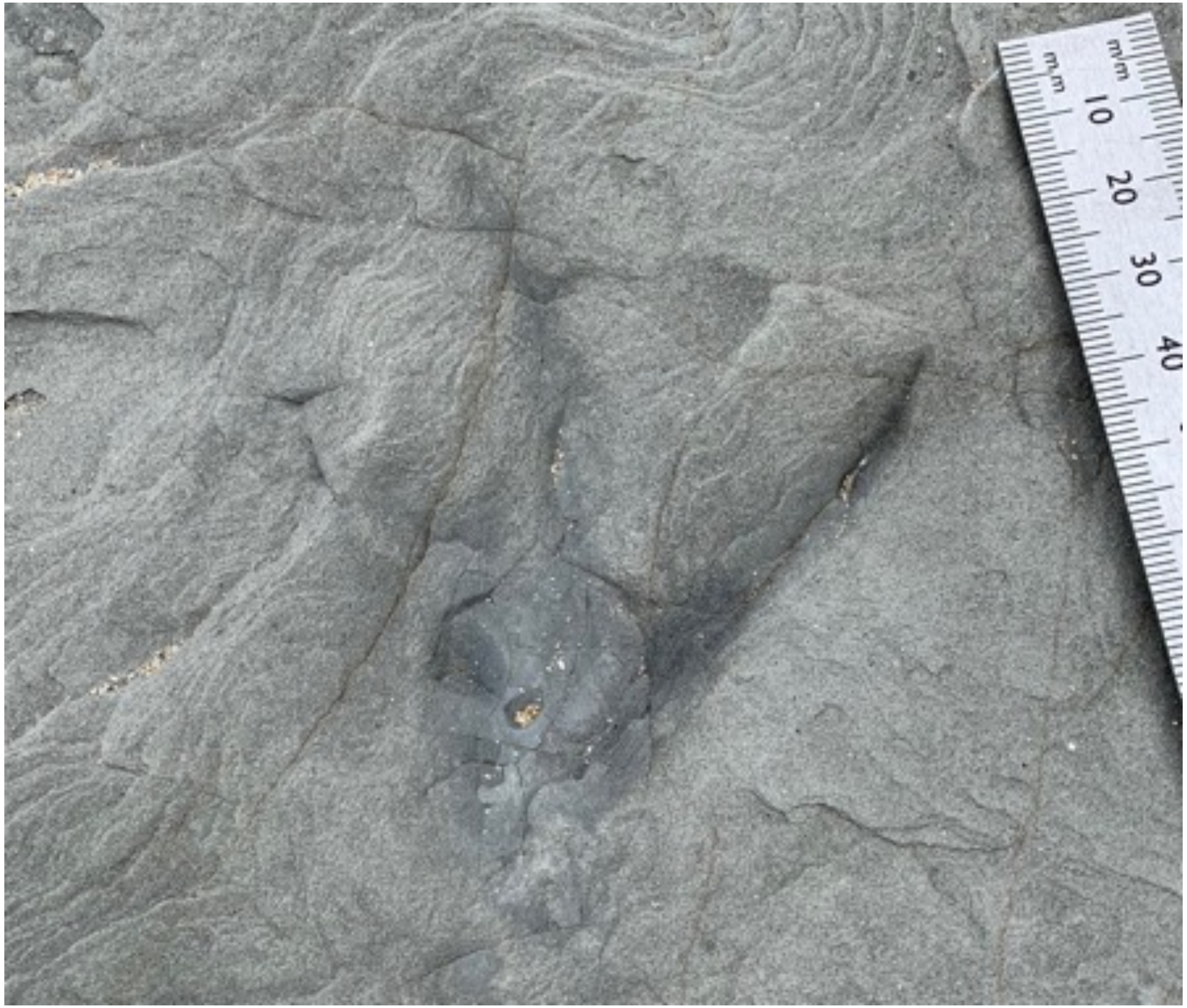


(No drawing)

- Negative relief epichnion.
- Anisodactyl incumbent, but missing one lateral digit (II or IV).
- Digits widened by erosion.
- Claw poorly defined on digit III but visible on lateral digit.
- Possible proximal webbing between lateral digit and digit III.
- Ichnogenus not determined.
