# Supplementary material for: Earliest known Gondwanan bird tracks: Wonthaggi Formation (Early Cretaceous), Victoria, Australia
Source: PLoS One. 2023 Nov 15;18(11):e0293308. doi: 10.1371/journal.pone.0293308 (PMC10651008; doi:10.1371/journal.pone.0293308)
Supplement: Attachment — Submitted filename: Review comments PONE-D-23-16348.pdf [file pone.0293308.s003.pdf]

Manuscript Number PONE-D-23-16348

"Earliest known Gondwanan bird tracks: Wonthaggi Formation (Early Cretaceous), Victoria, Australia"

I have very little to comment on regarding the actual text of the paper, so I will submit my comments here. The manuscript details a novel avian ichnofauna from Early Cretaceous Gondwana. In short, this is a tremendous find, and provides important information on the paleodiversity of early avians. Given the challenging nature of the present-day environment and the nature of the preservation, the authors approached the description of these tracks with an appropriate level of caution: suggestions of possible ichnotaxonomic assignments were made without formal designation. This provides the opportunity for more detailed examination of the tracks and the possibility for future surveys of the Footprint Flats and Honey Bay localities for more specimens.

Detailed measurements and data tables were provided, which will be very useful for future analyses, and the images in the paper and the supplemental information were clear and without overinterpretation. I agree with the authors that photogrammetric images are not required at this stage. Also, the encrusting tidal fauna on many of the tracks would provide a great deal of artificial relief to a heat map.

The only item that I would like to see expanded on may be the possible migratory potential for volant Early Cretaceous avians to polar regions during the spring and summer as we see in many of our modern analogs. Would a north-south migration of Barremian – Aptian shore and wading birds during the breeding season have any geographic barriers?

I recommend that PLOS One accept this paper for publication.
