# Supplementary material for: Earliest known Gondwanan bird tracks: Wonthaggi Formation (Early Cretaceous), Victoria, Australia
Source: PLoS One. 2023 Nov 15;18(11):e0293308. doi: 10.1371/journal.pone.0293308 (PMC10651008; doi:10.1371/journal.pone.0293308)
Supplement: Attachment — Submitted filename: PONE-D-23-16348 ML review.pdf [file pone.0293308.s004.pdf]

PLOS ONE

Earliest known Gondwanan bird tracks: Wonthaggi Formation (Early Cretaceous), Victoria, Australia

--Manuscript Draft--

**Manuscript Number:**	PONE-D-23-16348
**Article Type:**	Research Article
**Full Title:**	Earliest known Gondwanan bird tracks: Wonthaggi Formation (Early Cretaceous), Victoria, Australia
**Short Title:**	Earliest Gondwanan bird tracks from Early Cretaceous of Australia
**Corresponding Author:**	Anthony J. Martin<br><br>UNITED STATES
**Keywords:**	avian footprints; bird footprints; avian ichnology; Early Cretaceous; polar environments; avian evolution; avian paleobiogeography
**Abstract:**	The fossil record for Cretaceous birds in Australia has been limited to rare skeletal material, feathers, and two tracks, a paucity shared with other Gondwanan landmasses. Hence the recent discovery of 27 avian footprints and other traces in the Early Cretaceous (Barremian-Aptian, 128-120 Ma) Wonthaggi Formation of Victoria, Australia amends their previous rarity there, while also confirming the earliest known presence of birds in Australia and the rest of Gondwana. The avian identity of these tracks is verified by their tridactyl forms, thin digits relative to track lengths, wide divarication angles, and sharp unguals; three tracks also have hallux imprints. Track forms and sizes indicate a variety of birds as tracemakers, with some among the largest reported from the Early Cretaceous. Although continuous trackways are absent, close spacing and similar alignments of tracks on some bedding planes suggest gregariousness. The occurrence of this avian trace-fossil assemblage in circumpolar fluvial-floodplain facies further implies seasonal behavior, with trackmakers likely leaving their traces on floodplain surfaces during post-thaw summers.
**Order of Authors:**	Anthony J. Martin, Ph.D.
	Melissa Lowery
	Michael Hall, Ph.D.
	Patricia Vickers-Rich, Ph.D.
	Thomas H. Rich, Ph.D.
	Claudia Serrano-Brañas, Ph.D.
	Peter Swinkels
**Additional Information:**	
**Question**	**Response**
**Financial Disclosure**<br><br>Enter a financial disclosure statement that describes the sources of funding for the work included in this submission. Review the submission guidelines for detailed requirements. View published research articles from *PLOS ONE* for specific examples.<br><br>This statement is required for submission and **will appear in the published article** if	The author(s) received no specific funding for this work.

Powered by Editorial Manager® and ProduXion Manager® from Aries Systems Corporation

the submission is accepted. Please make sure it is accurate.<br><br>**Unfunded studies**<br>Enter: *The author(s) received no specific funding for this work.*<br><br>**Funded studies**<br>Enter a statement with the following details:<br>• Initials of the authors who received each award<br>• Grant numbers awarded to each author<br>• The full name of each funder<br>• URL of each funder website<br>• Did the sponsors or funders play any role in the study design, data collection and analysis, decision to publish, or preparation of the manuscript?<br>• **NO** - Include this sentence at the end of your statement: *The funders had no role in study design, data collection and analysis, decision to publish, or preparation of the manuscript.*<br>• **YES** - Specify the role(s) played.<br><br>* typeset	
**Competing Interests**<br><br>Use the instructions below to enter a competing interest statement for this submission. On behalf of all authors, disclose any competing interests that could be perceived to bias this work—acknowledging all financial support and any other relevant financial or non-financial competing interests.<br><br>This statement is required for submission and **will appear in the published article** if the submission is accepted. Please make sure it is accurate and that any funding sources listed in your Funding Information later in the submission form are also declared in your Financial Disclosure statement.<br><br>View published research articles from *PLOS ONE* for specific examples.	No authors have competing interests.

**NO authors have competing interests**<br><br>Enter: *The authors have declared that no competing interests exist.*<br><br>**Authors with competing interests**<br><br>Enter competing interest details beginning with this statement:<br><br>*I have read the journal's policy and the authors of this manuscript have the following competing interests: [insert competing interests here]*<br><br>* typeset	
**Ethics Statement**<br><br>Enter an ethics statement for this submission. This statement is required if the study involved:<br><br>• Human participants<br>• Human specimens or tissue<br>• Vertebrate animals or cephalopods<br>• Vertebrate embryos or tissues<br>• Field research<br><br>Write "N/A" if the submission does not require an ethics statement.<br><br>General guidance is provided below. Consult the submission guidelines for detailed instructions. **Make sure that all information entered here is included in the Methods section of the manuscript.**	This research did not involve any animal research, human subjects, or collections.

Powered by Editorial Manager® and ProduXion Manager® from Aries Systems Corporation

**Format for specific study types**<br><br>**Human Subject Research (involving human participants and/or tissue)**<br>• Give the name of the institutional review board or ethics committee that approved the study<br>• Include the approval number and/or a statement indicating approval of this research<br>• Indicate the form of consent obtained (written/oral) or the reason that consent was not obtained (e.g. the data were analyzed anonymously)<br><br>**Animal Research (involving vertebrate animals, embryos or tissues)**<br>• Provide the name of the Institutional Animal Care and Use Committee (IACUC) or other relevant ethics board that reviewed the study protocol, and indicate whether they approved this research or granted a formal waiver of ethical approval<br>• Include an approval number if one was obtained<br>• If the study involved *non-human primates*, add *additional details* about animal welfare and steps taken to ameliorate suffering<br>• If anesthesia, euthanasia, or any kind of animal sacrifice is part of the study, include briefly which substances and/or methods were applied<br><br>**Field Research**<br><br>Include the following details if this study involves the collection of plant, animal, or other materials from a natural setting:<br>• Field permit number<br>• Name of the institution or relevant body that granted permission	
**Data Availability**<br><br>Authors are required to make all data underlying the findings described fully available, without restriction, and from the time of publication. PLOS allows rare exceptions to address legal and ethical concerns. See the PLOS Data Policy and FAQ for detailed information.	Yes - all data are fully available without restriction

A Data Availability Statement describing where the data can be found is required at submission. Your answers to this question constitute the Data Availability Statement and **will be published in the article**, if accepted.<br><br>**Important:** Stating 'data available on request from the author' is not sufficient. If your data are only available upon request, select 'No' for the first question and explain your exceptional situation in the text box.<br><br>Do the authors confirm that all data underlying the findings described in their manuscript are fully available without restriction?	
**Describe where the data may be found in full sentences. If you are copying our sample text, replace any instances of XXX with the appropriate details.**<br><br>• If the data are **held or will be held in a public repository**, include URLs, accession numbers or DOIs. If this information will only be available after acceptance, indicate this by ticking the box below. For example: *All XXX files are available from the XXX database (accession number(s) XXX, XXX.).*<br>• If the data are all contained **within the manuscript and/or Supporting Information files**, enter the following: *All relevant data are within the manuscript and its Supporting Information files.*<br>• If neither of these applies but you are able to provide **details of access elsewhere**, with or without limitations, please do so. For example:<br><br>*Data cannot be shared publicly because of [XXX]. Data are available from the XXX Institutional Data Access / Ethics Committee (contact via XXX) for researchers who meet the criteria for access to confidential data.*<br><br>*The data underlying the results presented in the study are available from (include the name of the third party*	All relevant data are within the manuscript and its Supporting Information files.

and contact information or URL).<br>• This text is appropriate if the data are owned by a third party and authors do not have permission to share the data.<br><br>* typeset	
Additional data availability information:	

Powered by Editorial Manager® and ProduXion Manager® from Aries Systems Corporation

Manuscript Click here to access/download;Manuscript;Martin-et-al-Final.docx

Earliest known Gondwanan bird tracks: Wonthaggi Formation (Early Cretaceous), Victoria, Australia

Anthony J. Martin[1*], Melissa Lowery[2] ¶, Michael Hall[2]¶, Patricia Vickers-Rich[2]¶, Thomas H. Rich[3]¶, Claudia I. Serrano-Brañas[4,5]¶, Peter Swinkels[3]¶

[1] Department of Environmental Sciences, Emory University, Atlanta, GA 30322, USA

[2] School of Earth, Atmosphere, and Environment, Monash University, Clayton, VIC 3800 Australia

[3] Museum Victoria, PO Box 666, Melbourne, Victoria 3001, Australia

[4] Benemérita Escuela Normal de Coahuila, Calzada de los Maestros 858, Zona Centro Poniente, Saltillo, Coahuila, 25000, Mexico

[5] Department of Paleobiology, National Museum of Natural History, Smithsonian Institution, Washington, DC, 20013-7012, USA

*Corresponding author

Email: geoam@emory.edu (AJM)

¶ These authors contributed equally to this work.

Abstract

The fossil record for Cretaceous birds in Australia has been limited to rare skeletal material, feathers, and two tracks, a paucity shared with other Gondwanan landmasses. Hence the recent discovery of 27 avian footprints and other traces in the Early Cretaceous (Barremian-Aptian, 128-120 *Ma*) Wonthaggi Formation of Victoria, Australia amends their previous rarity there, while also confirming the earliest known presence of birds in Australia and the rest of Gondwana. The avian identity of these tracks is verified by their tridactyl forms, thin digits relative to track lengths, wide divarication angles, and sharp unguals; three tracks also have hallux imprints. Track forms and sizes indicate a variety of birds as tracemakers, with some among the largest reported from the Early Cretaceous. Although continuous trackways are absent, close spacing and similar alignments of tracks on some bedding planes suggest gregariousness. The occurrence of this avian trace-fossil assemblage in circumpolar fluvial-floodplain facies further implies seasonal behavior, with trackmakers likely leaving their traces on floodplain surfaces during post-thaw summers.

Introduction

Present-day Australia hosts a remarkable diversity of birds, with their evolutionary lineages well represented in the Cenozoic fossil record there [1–4]. In contrast, evidence of Mesozoic birds in Australia is scanty, denoted by isolated skeletal elements, a few feathers, and two footprints in Cretaceous strata [5–11]. The oldest definite body fossil evidence for birds in Australia consist of a furcula and a flight feather from the Early Cretaceous (Valangian-Aptian)

Wonthaggi Formation of Victoria [8, 11]. As for the rest of Gondwana, the only Early Cretaceous bird outside of Australia is *Cratoavis cearensis* from the Crato Formation (Aptian) of Brazil [12–13], with bird tracks reported from the Late Cretaceous of Argentina (14–17) and Tunisia [18]. Given so few Mesozoic avian fossils, then, we have little evidence of when birds got their start in Australia and most other Gondwanan landmasses, let alone how they interacted with their environments.

Hence the recent discovery of 27 bird tracks and additional traces in Barremian-Aptian (128-120 *Ma*) circumpolar fluvial facies of the Wonthaggi Formation of Victoria, Australia represents a significant advance in the fossil record of Early Cretaceous birds in Australia and the remainder of Gondwana. These trace fossils not only affirm an avian presence at this time and place, but the variety of sizes and track forms also imply a diverse avifauna in formerly polar environments of Australia [8–10, 19–20]. Moreover, some specimens are among the largest documented bird footprints from the Early Cretaceous, rivaling sizeable avian tracks from east Asia [21]. Although outcrop expressions of the tracks are limited and no continuous trackways are evident, close spacing and similar orientations of tracks on some bedding planes further point to gregariousness. This evidence, as well as the sedimentological conditions of circumpolar fluvial-floodplain facies, suggest seasonally linked behaviors and preservation of their tracks during polar summers, while also providing glimpses of the early history, biodiversity, and adaptations of birds in Gondwana.

Materials and Methods

Nearly all tracks described in this study were discovered by one of us (ML) in 2020-2022 while prospecting for fossils on Bass Coast marine platforms near Inverloch, Victoria. The remainder of tracks were noticed in May 2022 during a site visit by another of us (AJM) in the presence of most coauthors. Of the 27 tracks documented in this study, 26 were at a locality dubbed “Footprint Flats,” and one was 1.2 km southwest of there, at a place nicknamed “Honey Bay” (Figure 1). For the track at Honey Bay, it was simply labeled HB-1, whereas tracks at Footprint Flats were assigned specimen numbers for each of five beds in stratigraphic order from oldest to youngest, with FF-1-1 on the lowest bed through FF-5B-5 on the uppermost bed (Figure 2). The top two assemblages at Footprint Flats are in a silty sandstone bed but on different bedding planes, hence tracks in this bed were assigned to two levels, 5A (lower) and 5B (upper). Lithology, physical sedimentary structures, and additional biogenic sedimentary structures (e.g., invertebrate burrows) associated with the tracks were also noted and evaluated. Beds on marine platforms at both localities were dipping 10-15° east-northeast, resulting in narrow exposures of bedding planes that likely prevented our observing continuous trackways. With no sure way to tell whether tracks were from the same trackmakers or not, all were treated as individual specimens.

Fig. 1. Locality map of Wonthaggi Formation avian tracksites, Victoria, Australia.
(A) Victoria (VIC), Australia, with approximate outcrop area of Wonthaggi Formation indicated. (B) Wonthaggi Formation coastal exposures of tracksites for this study, “Honey Bay” (HB) at S 38° 39.9’, E 145° 40.5’, and “Footprint Flats” (FF) at 38° 39.4’, E 145° 41.0’, separated by a northeast-southwest straight-line distance of 1.2 km. Image from EarthExplorer (https://earthexplorer.usgs.gov), accessed May 11, 2023.

Fig. 2. Stratigraphic section of Wonthaggi Formation at Footprint Flats locality. Sandstone bed numbers (FF-1 through FF-5A-B) for stratigraphic levels bearing tracks. "F" and red lines denote faults, with one fault less than a meter above FF-1 and another about 11 meters above FF-5. Colors used in this section do not match those of lithofacies.

Tracks described in this study were measured with Mitutoyo™ digital calipers and track measurements were recorded to the nearest millimeter. Measured parameters included track length, track width, digit lengths and widths, and interdigital angles between digits I-II (if digit I was evident), digits II-III, and digits III-IV (Figure 3). Track length was measured from the rearward-most point where the midlines of digits II-IV met on an axis defined by digit III, and track width was measured from the outermost edges of digits II and IV. Extended track length was also noted for tracks with digit I. Because nearly all tracks were preserved as negative-relief features on bed tops, measurements were taken as minimum outlines from where track bottoms ("floors") met their sides ("walls"), rather than where they intersected uppermost bedding planes. Length:width ratios were calculated from length and width measurements, and divarication was the sum of interdigital angles II-III and III-IV. Track orientations were measured as azimuths with a Silva™ compass placed above the linear trend of digit III on each track.

Fig. 3. Measurements applied to avian tracks in this study. L = length, W = width, EL = extended length (with digit I), DL = digit length, DW = digit width, IA-I-II = interdigital angle for digits I-II (if present), IA-II-III = interdigital angle for II-III, IA-III-IV = interdigital angle for III-IV. Total divarication is the sum of interdigital angles for digits

II-IV. Example track is depicted with average interdigital angles for all tracks with digits II-IV in this study (n = 25), with digits II-III = 53°, digits III-IV = 63°, and a divarication of 116°.

Descriptive statistics – such as mean and standard deviation – were then applied to tracks expressed as tridactyl patterns (digits II-IV). As for qualitative data, we noted presence or absence of phalangeal pads, ungual (claw) impressions, interdigital webbing, and sedimentary structures outside of footprint outlines formed by tracemaker interactions with the host sediment [9, 22–24]. Orientations of claws impressions on digits II and IV relative to the track midlines were also noted, as these are often used as a diagnostic criterion for bird tracks [25–28]. One of us (AJM) made detailed line drawings of tracks and closely associated sedimentary structures (e.g., bedding, burrows), with outline sketches of the inferred foot anatomy overlain for each footprint.

Tracks were exposed on an active marine platform, and hence have been subjected to physical weathering from daily tidal and wave activity, as well as bioerosion and encrustation by algae, barnacles, and molluscans. Most tracks examined in this study were only exposed at low tide, attesting to conditions that assured ephemeral preservation. For example, in one spot, seven tracks were erased from a bedding plane at Footprint Flats (FF-5) over the course of 18 months, from November 2020 to May 2022. Given these conditions, one of us (PS) photographed and recorded GPS coordinates of track-bearing bedding planes, then made silicone molds and polyester-resin casts, which will serve as lasting and tactile (non-digital) records of track forms and dimensions. (S1 Docx). For tracks eroded since their initial discovery, we measured track parameters from their polyester-resin casts, with those specimens indicated (Table 1). In May 2022, we also placed a cast of seven tracks from bed FF-5 onto their former bedding planes to

measure in-situ orientations of those footprints on the two surfaces of that bed (levels 5A and 5B; Figure 4).

Table 1. Measurements and descriptive statistics of Wonthaggi Formation tracks at Honey Bay (n = 1) and Footprint Flats (n = 26).

(A) Anisodactyl incumbent tracks (digits II-IV) from Footprint Flats (FF) and Honey Bay (HB), Wonthaggi Formation, Victoria, Australia.

Track	L	W	L/W	D2-L	D2-W	D3-L	D3-W	D4-L	D4-W	D23-A	D34-A	TD	Az
FF-1-1	44	75	0.59	34	5	46	6	43	5	55	70	125	255
FF-1-2	45	65	0.69	39	ND	45	4	41	5	55	64	119	190
FF-1-4	64	97	0.66	57	4	64	5	58	4	53	73	126	210
FF-2-1	58	89	0.64	53	5	58	6	57	5	53	58	111	170
FF-3-1	56	78	0.72	44	1	56	11	48	13	58	64	122	170
FF-3-2	88	92	0.96	58	9	88	11	58	12	58	67	125	340
FF-3-3	85	96	0.89	57	7	85	12	62	15	53	60	113	290
FF-3-5	53	72	0.74	41	7	53	9	41	8	50	69	119	315
FF-3-6	117	132	0.89	67	9	117	1	74	6	55	64	119	55
FF-4-1	59	76	0.78	43	7	59	6	55	5	64	65	129	155
FF-4-2	77	98	0.79	62	3	77	6	57	6	52	60	112	340
FF-4-3	102	142	0.72	90	11	102	9	84	7	52	55	107	160
FF-4-4	92	116	0.79	75	1	92	9	77	16	53	60	113	165
FF-4-5	91	122	0.75	78	14	91	11	86	14	57	60	117	330
FF-4-6	102	121	0.84	59	4	102	11	70	6	52	56	108	135
FF-5A-1	55	84	0.65	47	3	55	3	49	5	52	64	116	230
FF-5A-2	61	82	0.74	41	4	61	4	51	3	46	55	101	230

FF-5A-3	79	84	0.94	53	5	79	5	69	nd	54	61	115	235
FF-5A-4	63	80	0.79	45	3	63	5	45	3	55	64	119	130
FF-5A-5	69	78	0.88	44	4	69	4	35	4	49	52	101	270
FF-5B-1	69	76	0.91	42	4	69	6	44	3	45	61	106	140
FF-5B-3	91	116	0.78	69	5	91	6	54	6	48	64	112	105
HB-1	46	77	0.6	34	5	46	6	43	5	67	71	138	240

(B) Anisodactyl tracks (digits I-IV) from Footprint Flats, Wonthaggi Formation, Victoria, Australia.

Track	L	W	EL	L/W	D1-L	D1-W	D2-L	D2-W	D3-L	D3-W	D4-L	D4-W	D12-A	D23-A	D34-A	TD	Az
FF-1-3	76	96	86	0.79	18	4	61	5	76	7	74	5	127	48	56	104	265
FF-5B-2	83	118	98	0.7	19	4	48	6	83	3	67	6	88	54	69	123	150
FF-5B-4	93	115	102	0.81	21	5	43	6	93	8	65	8	92	55	69	124	160

(C) Incomplete anisodactyl incumbent tracks (digit III and one lateral digit) from Footprint Flats, Wonthaggi Formation, Victoria, Australia

Track	L	(W)	L/W	D2-L	D2-W	D3-L	D3-W	D23-A	Az
FF-3-4	82	94*	0.87*	43	9	82	14	54	275
FF-5B-5	66	69*	0.96*	61	5	66	7	47	140

(D) Summary descriptive statistics for anisodactyl incumbent and anisodactyl tracks with digits II-IV (n = 25).

	L	W	L/W	D2-L	D2-W	D3-L	D3-W	D4-L	D4-W	D23-A	D34-A	TD	Az
Mean	74	95	0.77	53	5	74	7	58	7	54	63	116	209
Standard Deviation	20	21	0.01	14	3	20	3	14	4	5	6	9	76

Legend: (A) Track = specimen number; L = length (mm); W = width (mm); L/W = length:width ratio; D2-L = digit II length; D2-W = digit II width; D3-L = digit III length; D3-W = digit IV width; D4-L = digit IV length; D4-W = digit IV width; D23-A = angle between digits II and III (degrees); D34-A = angle between digits III and 4; TD = total divarication (sum of D23-A and D34-A); Az = azimuth (compass degrees).

(B) Track = specimen number; L = length (mm); W = width (mm); EL = extended length (with digit I); L/W = length:width ratio; D1L = digit I length; D1W = digit I width; D2-L = digit II length; D2-W = digit II width; D3-L = digit III length; D3-W = digit IV width; D4-L = digit IV length; D4-W = digit IV width; D12-A = angle between digits I and II; D23-A = angle between digits II and III (degrees); D34-A = angle between digits III and 4; TD = total divarication (sum of D23-A and D34-A); Az = azimuth (compass degrees).

(C) L = length (mm); (W) = extrapolated width (based on 2X digit 3 + lateral digit);

(D) L = length (mm); W = width (mm); L/W = length:width ratio; D2-L = digit II length; D2-W = digit II width; D3-L = digit III length; D3-W = digit IV width; D4-L = digit IV length; D4-W = digit IV width; D23-A = angle between digits II and III (degrees); D34-A = angle between digits III and 4; TD = total divarication (sum of D23-A and D34-A); Az = azimuth (compass degrees).

Fig. 4. Polyester-resin cast duplicating tracks in rocky intertidal environment at Footprint Flats (FF) locality.

(A) Bedding planes of FF-5A (lower surface) and FF-5B (upper surface), with seven tracks (circled) photographed just before molding on November 11, 2020; ruler = 15 cm long. (B) Resin cast of bedding plane, with same seven tracks indicated; scale with centimeters. See text for key to specimen numbers, Table 1 for measurements of tracks, and S1 Docx. for description of molding and casting methods.

Owing to safety protocols in Australia necessitated by the Covid-19 pandemic throughout 2020-2022, we limited the number of participants and time together in the field while documenting tracks and their geological context on site. This documentation included field mapping by one of us (MH), and others taking two-dimensional digital photos, measurements, and descriptions, and making labeled field sketches. As mentioned previously, one of us (AJM) later made line drawings based on photographs taken from 2020-2022. Although we did not apply photogrammetric methods recommended for vertebrate footprints [29], we are nonetheless confident that our non-photogrammetric methods were more than adequate for accurately describing and interpreting the Wonthaggi Formation footprints. If future researchers wish to test and replicate our results, we encourage them to examine polyester-resin casts of the tracks, which will be curated by Museum Victoria. These casts then can be further studied through photogrammetry, laser scanning, and other digital methods.

Results

Study Area and Stratigraphy

All tracks described in this study were exposed in marine-platform outcrops of the Wonthaggi Formation west of Inverloch, Victoria. As mentioned before, tracks at Footprint Flats were preserved on five stratigraphic levels (FF-1 to FF-5) on tops of ripple-bedded and silty to fine- to medium-grained sandstones; these sandstones were interbedded with mudstones and thin (< 1 cm thick) coal beds in a 7-m interval (Figure 2). The single track at Honey Bay was similarly on the top surface of a ripple-bedded fine-grained sandstone. Based on palynological data from the Wonthaggi Formation in this area, track-bearing strata at both localities are interpreted as late Barremian to early Aptian (128-120 *Ma*) [10, 30]. Two faults interrupt the stratigraphic sequence at the Footprint Flats site, with FF-1 just below the one fault and the remaining four track-bearing strata below the second fault. However, we did not detect any repetition of strata at this locality as a result of faulting.

The Wonthaggi Formation is a thick sequence of clastic conglomerates, sandstones, and mudstones formed mostly by alluvial, fluvial, and lacustrine processes in rift valleys of the Gippsland Basin [31–34]. Based on a wide range of geological evidence, southern Australia was affected by glaciation during Early Cretaceous [35–36]; the paleolatitude of the Gippsland basin was 75-80° S, with southern Australia diverging from Antarctica then [37; 38; 39; 19; 40; 33, 41; 20]. Although mean annual air temperatures of polar regions during the Cretaceous were higher than those of modern settings, these ecosystems and their communities still experienced freezing temperatures and months of darkness [20, 39–44]. Ice accumulation during winter months led to torrential run-off and high depositional rates in polar springs, followed by waning flows and

emergent floodplains and channel margins [32-34, 39]. Given these climatic and sedimentological conditions, most biogenic sedimentary structures – such as footprints and burrows – were more likely formed and preserved on floodplain surfaces exposed during lower flow regimes, such as during late springs and summers. Hydrological, sedimentological, and ichnological conditions of a modern fluvial point bar during summer in the Arctic Circle were used as analogs for the formation and preservation of the trace fossils reported here [24].

Descriptions of Lithofacies and Tracks

Most tracks at Footprints Flats were evident as negative-relief epichnia, forming impressions on bed tops of 10-20 cm thick ripple-bedded sandy siltstones and fine-grained sandstones. However, a few footprints were also preserved as positive-relief epichnia, with their sediment fills more resistant to weathering compared to the host substrate. Tracks are preserved on at least five stratigraphic levels, with the highest on different bedding planes within the same stratum. The single track from Honey Bay (HB-1) was expressed in positive-relief (1-3 mm) on a 15-cm thick ripple-bedded fine-grained sandstone, but was also definable as a disturbance of underlying and surrounding bedding (Figure 5).

Fig. 5. Avian track in sandy siltstone from Wonthaggi Formation at Honey Bay locality. (A) Footprint HB-1, with digits II-IV indicated. (B) Line drawing of HB-1 footprint with overlay of inferred foot morphology. Note deformation of bedding immediately behind digit III and the remainder of the track, suggesting the possible effect of a digit I (I?). Scale = 1 cm in both.

Using the terminology of Elbroch and Marks [45], tracks were categorized as anisodactyl (digits I-IV present) or anisodactyl incumbent (only digits II-IV present). Tracks with only digit III and one lateral digit preserved were termed as incomplete. Of these categories, anisodactyl incumbent tracks are the most common (n = 22), followed by anisodactyl (n = 3), and incomplete (n = 2). Other than the 25 complete and two incomplete tracks, several single-digit and/or ungual impressions were also present on bedding planes, such as those near or cross-cut by more complete footprints (Figure 6). Although tracks represented by single-digit or ungual traces were not included in our total track count, these are still worth noting as additional evidence of tracemakers.

Fig. 6. **Ungual and digit impressions near a more clearly defined avian footprint in the Wonthaggi Formation, Footprint Flats.**

(A) Two ungual prints next to FF-4-5 (left) and a nearby incomplete track (two digits registered), with each ungual and digit denoted by arrows; ungual print behind FF-4-5 gives misleading appearance of a digit I for that track. (B) Overlay of inferred foot morphology for FF-4-5 and estimated anisodactyl-incumbent track based on two ungual prints. (C) Overlay of inferred foot morphology for incomplete track with only partial prints of two digits. For paired ungual and digit prints, one is presumed as digit III and the other as either II or IV. Photo scale = 5 millimeter squares.

Most tracks from Footprint Flats are relatively shallow (1-8 millimeters), with some parts impressed deeply enough to intersect underlying mudstone layers, but other portions nearly coincided with uppermost bedding planes. As a result, few tracks expressed the full foot anatomy

of their makers, with incomplete prints as the norm (Figure 7). Although skin impressions were not observed in any tracks, outlines of phalangeal pads were discernable in a few, such as FF-4-1, FF-4-3, and FF-4-5. Most digits taper distally along their lengths, and lateral digits typically end with thin and pointed ungual impressions that curve posteriorly. In some specimens, incomplete digits were only defined only by distal claw imprints and/or linear disruptions of bedding (Figure 8).

Fig. 7. **Wonthaggi Formation avian tracks with uneven depths of digit impressions.**
(A,B) Photo (A) and line drawing (B) of track FF-4-3, interpreted as a right pes, with overlay of inferred foot morphology. Digits II and III penetrated to the underlying mudstone layer, but with shallow and distal impressions of digit IV. (C,D) Photo (C) and line drawing (D) of FF-4-4, interpreted as a right pes with overlay of inferred foot morphology. Digits II and IV reached the underlying mudstone layer, but digit III is barely registered. Proximal interdigital webbing is inferred for both tracks, but these features also may be from foot-sediment interactions. Scale = 5 cm in all parts; see Figure 2 and Table 1 for stratigraphic position and specimen measurements, respectively.

Fig. 8. **Wonthaggi Formation avian track with digits defined by distal ends and ungual impressions.**
(A) Photo and (B) line drawing of track FF-5B-1 (polyester cast), interpreted as right pes, with ungual imprint of digit IV and slight disruption of bedding (arrows) as evidence for that digit, and inferred foot morphology overlain. Scale in both = 1 cm.

Proximal interdigital webbing (semipalmated) seemed present in a few tracks, such as FF-4-3, FF-4-4, and FF-4-5 (Figures 6-7), but these and other anatomical features were difficult to distinguish from sedimentary structures formed by an interaction of foot movement and original sedimentary conditions. For example, track FF-1-4 has a swath of disturbed sediment behind digits II-IV, obscuring any outline of a probable digit I impression there (Figure 9).

Fig. 9. Differing sedimentary expressions of digit I in Wonthaggi avian tracks.

(A,B) Photo and line drawing FF-1-3, with inferred foot morphology overlain. Because digit I is clearly defined, this track is classified as anisodactyl. (C,D) Photo and line drawing of FF-1-4. A probable digit I impression in FF-1-4 is obscured by disturbed sediment behind digits II-IV. Because of this uncertainty, the latter track was classified as anisodactyl incumbent. Scale = 5 cm in both B and D.

Other extramorphological structures are associated with two of the three anisodactyl tracks, FF-5B-2 and FF-5B-4, both of which have prominent ridges in front of digits II-IV, as well as linear grooves and ridges outlining digit I (Figures 10, 11). Two anisodactyl incumbent tracks that nearly intersect one another, FF-5A3 and FF-5A-4, as well as a nearby single-digit impression, also had prominent ridges around track outlines (Figure 12). These sedimentary structures are likely related to foot movement interacting with sediments, explained more later.

Fig. 10. Anisodactyl track FF-5B-2.

(A) Outcrop view of track on November 11, 2020. (B) Polyester-resin cast of track. (C) Line drawing with overlay of inferred foot morphology. Track is interpreted as a right pes with full

anisodactyl expression, sedimentary structures (ridges) in front of digits II-IV, and a thin hooked groove associated with digit I. Scale bar = 5 cm in all parts.

Fig. 11. Anisodactyl track FF-5B-4.

(A) Outcrop view of track on May 25, 2022. (B) Line drawing with overlay of inferred foot morphology. Track is interpreted as right pes, with sedimentary structures (ridges) in front of digits II-IV and a thin linear groove associated with digit I, presumably imparted by the ungual. Scale bar = 5 cm in both parts.

Fig. 12. Closely associated Wonthaggi tracks and single-digit impression with prominent extramorphological features.

(A) Outcrop view of FF-5A3, FF-5A-4, and single digit (SD); photo taken on November 11, 2020. (B) Fiberglass cast of tracks and single-digit impression. (C) Line drawings of FF-5A-3 and FF-5A-4 tracks, single-digit impression, and extramorphological structures, with overlays of inferred foot morphology for each track and digit. Ruler in A is in millimeters, scale bar in B and C = 5 cm.

In terms of descriptive statistics, average sizes and standard deviations for all tracks with digits II-IV expressed were 74 $\pm$ 20 mm long and 95 $\pm$ 21 mm wide, with ranges of 44 to 117 mm long and 65 to 142 mm wide (n = 25; Table 1, Figure 13). Digit II and digit IV lengths averaged 53 $\pm$ 14 mm and 58 $\pm$ 14 mm, respectively. Digit widths varied from 3 to 14 mm, but digits II, III, and IV averaged 5-7 mm wide, or less than 10% of footprint lengths. Length:width ratios of tracks ranged from 0.59 to 0.96, with a mean of 0.77. Interdigital angles for digits II-III

ranged from 45° to 67°, with a mean and standard deviation of 53.5 ± 4.8°, whereas interdigital angles for digits III-IV were from 52° to 71°, and with a mean and standard deviation of 63 ± 6°. Total divarication angles between digits II and IV ranged from 101° to 139° and averaged 116 ± 9°.

Fig. 13 – Size-frequency distributions of lengths and widths for Wonthaggi Formation tracks with digits II-IV.

(A) Track lengths. (B) Track widths. Bins are 10 mm for each parameter (e.g., 40-49 mm), with number of tracks in each bin and mean, standard deviation, minimum, and maximum values; lengths exclude extended lengths for tracks with digit I. See Table 1 for specimen numbers and values.

However, sources of potential variations and errors in track measurements include their exposure on a marine platform, which resulted in considerable physical and biological weathering of some track features. For example, a few tracks on bed FF-3 had widened digits altered by erosion, whereas others had barnacles affixed to digit impressions and gastropods actively grazing algae on their surfaces (Figure 14).

Fig. 14. Wonthaggi bird tracks affected by modern erosion and marine organisms at Footprint Flats locality.

(A) FF-3-1-1, interpreted as left pes, with widened (eroded) digits and encrusted by algae, barnacles, and gastropods. (B) FF-3-5, interpreted as right pes, with barnacles and gastropods on track center and within digits. Scale bar = 5 cm in both.

Folding and faulting of Wonthaggi strata rendered track orientations meaningless in a regional sense, but these data were applicable to local clusters of tracks sharing the same bedding plane. For example, at Footprints Flats, four closely spaced tracks on FF-3 and a possible fifth track had southwest-to-northwest orientations between 235° and 340° (Figure 15A). On FF-5A, four of five closely spaced tracks were within a narrower range of orientations, from 230° to 270°, whereas two tracks on stratigraphic level 5B were identically oriented at 165° (Figure 15B). These data help test for possible tracemakers' group behaviors, discussed later.

Fig. 15. Similar orientations of closely spaced bird-track assemblages on Wonthaggi bedding planes.

(A) Bed FF-3, with azimuth orientations between 235-340° for tracks on bedding plane. Depression denoting possible track (PT) also points in northwestern quadrant at 310°; photo scale in centimeters. (B) Bed FF-5, with azimuth orientations between 230-270° for four of five tracks on lower bedding plane (5A) and identical azimuths of 165° for two tracks on upper bedding plane (5B). Ruler (left) = 15 cm long.

Although the Wonthaggi tracks vary in sizes and forms, they share the following morphological traits: anisodactyl or anisodactyl incumbent, with three forward-pointing digits (II-IV); a limited size range (<15 cm length); relatively thin digits relative to footprint length (<10%); divarication angles between digits II and IV that consistently exceeded 90°, which corresponded with track length:width ratios of about 0.8; and narrow, pointed ungual impressions at digit ends. In most instances, digit II and IV ungual imprints also curved

posteriorly with respect to the main axis of their digits (Figures 6-10). As mentioned previously, at least three tracks (FF-1-3, FF-5B-2, FF-5B4), and perhaps a fourth (FF-1-4) also had a digit I imprint, resulting in fully anisodactyl tracks. All other tracks with no clearly defined digit I were considered as anisodactyl incumbent. Proximal webbing may be present at intersections between digit III and digit II or IV of some tracks, but none have evidence of distal webbing as fully palmate tracks [45].

Invertebrate trace fossils in track-bearing strata at Footprint Flats include small (5-10 mm wide) and scarce horizontal to inclined burrows intersecting rippled bed tops that occasionally occur with tracks (Figure 16). These burrows are evident as raised, circular to oval outlines intersecting upper bedding planes; a few had sandy linings (Fig. 16B,C). None of the burrows are distinctive enough to assign ichnogenera, but they nevertheless indicate infaunal invertebrates were living in these sediments. As for other vertebrate trace fossils, some strata immediately above and below the studied intervals at both Footprint Flats and Honey Bay also preserve larger non-avian theropod tracks, which are the subject of a separate study by the authors.

Fig. 16. Invertebrate burrows and avian tracks in Wonthaggi Formation.

(A) Specimen FF-4-2 with oval cross-sections of sand-lined burrows closely associated with track (arrows); scale = 5 mm squares. (B) Close-up of burrow cross-sections on left side of track, with two sand-lined nearly intersecting on right; scale = 1 cm. (C) Specimen FF-5B-4 with circular cross-sections of unlined burrows (arrows); scale = 5 mm squares. (D) Line drawing of FF-5B-4 denoting locations of circular to semi-circular burrow cross-sections with track; scale = 5 cm.

Discussion

Probable Identities of Trackmakers and Sedimentary Conditions

All of the described Wonthaggi tracks here are interpreted as those of theropod dinosaurs, but more specifically of avian theropods. Because every track is minimally tridactyl – either anisodactyl or anisodactyl incumbent – they readily eliminate a wide range of other potential tetrapod tracemakers documented from body fossils in the Wonthaggi Formation, such as temnospondyls, chelonians, ceratopsians, ankylosaurs, and mammals [10]. Tridactyl tracks in Cretaceous strata are normally attributed to two broad categories of dinosaurian tracemakers, ornithopods and theropods [46], with theropod tracks further split into non-avian or avian. However, ornithopod tracks typically have equant widths and lengths (length:width ratios about 1.0), thick digits relative to track lengths, blunt ungual impressions, digit II-IV divarication of 90° or less, and no hallux [47 and references therein]. As a result, we do not regard any of the Wonthaggi tracks at either locality described here as those of ornithopods.

As for distinguishing fossil avian tracks from those of non-avian theropods, Lockley and others [25] listed the following diagnostic criteria for avian footprints: (1) resemblance to modern bird tracks; (2) relatively smaller sizes; (3) indefinite phalangeal pad impressions; (4) wide divarication angles between digits II and IV (110–120° or more); (5) a rearward-pointing hallux; (6) thin claws; (7) claws on digits II and IV curving away from digit III; and (8) narrow digits relative to footprint length. This checklist has been amended since [27; 46, 48] and augmented by refined descriptions of modern bird tracks as analogues [45, 49].

Given these guidelines, Wonthaggi Formation tracks described in this study meet most of the diagnostic criteria for an avian identity, and some fulfill all prescribed traits, including a posterior-pointing hallux. Those tracks lacking a hallux are relatively small (with some exceptions), have indefinite phalangeal pads, wide divarication angles, thin claws, and narrow digits relative to track length. More than half of the tracks (n = 15) have claw impressions on digits II and IV that curve posteriorly from a midline defined by digit III. Non-avian theropods are the closest alternative tracemakers, but their tracks are characterized by: (1) larger sizes; (2) definite phalangeal pads; (3) narrower divarication between digits II and IV (typically < 90°) and hence length:width ratios exceeding 1.0; (4) no hallux; (5) thicker claws; (6) claw impressions of digits II and III turned medially, but that of digit IV is turned laterally; and (7) thicker digits relative to track length [25, 27, 46, 48].

The lack of skin impressions or other fine anatomical details of the Wonthaggi tracks, as well as their common occurrence on rippled bed tops, points toward their original preservation either as undertracks, surface tracks that were shallowly impressed, or tracks that were partially eroded before burial. Digit impressions of some tracks on bedding-plane surfaces, such as those of stratum 1 at Footprint Flats (FF-1-1, FF-1-2, etc.), were also evident primarily as linear disturbances of underlying bedding. This expression implies that sediments were moist and collapsed into footprints after feet were pulled out, similar to results from neoichnological experiments using sediments of varying grain size and water content [50–51]. For example, Falk and others [51] demonstrated that finer-grained (silt-sand) sediments preserve more details in tracks, and that sediment moisture contents of 6.7-8.8% (by volume) result in thinner and tapered digits that are also less likely to record pad impressions. Most Wonthaggi tracks similarly have

thin and tapered digits with rare pad impressions, thus these more likely formed in moist sediments on fluvial floodplains.

The fine-grained sediments of the 7-m thick track-bearing sequence at Footprint Flats, consisting of rippled sandy siltstones and silty sandstones bounded by mudstones, suggest these facies were distal overbank-floodplain deposits formed by low flow regimes, contrasting with high-energy conglomeratic alluvial- and fluvial-channel bedforms [32–34, 39]. Considering the circumpolar setting of these facies, high discharge and flow regimes were likely associated with spring thaws and consequent run-off from upland areas, whereas lesser flows were more typical of late spring or early summer. A modern Arctic fluvial point bar in the Colville River shows the same seasonal dynamics, in which massive discharges followed spring thaws, but summer resulted in ample exposure of the point bar, enabling tracemaking activities and preservation of their traces (24).

Ichnotaxonomy

Modern weathering and erosion affected Wonthaggi track preservation from their exposure on marine platforms, a daily attrition inflicted by tides, waves, and rocky-intertidal organisms (Figure 14). Owing to these processes and incomplete or indistinct preservation of foot anatomy, previously established ichnotaxa are not readily applied to most tracks. This cautionary approach is especially justified when the Wonthaggi tracks are compared to better preserved and far more abundant specimens of avian-attributed ichnotaxa from Cretaceous tracksites outside of Australia, such as those in east Asia and North America [27, 52–59].

With that said, a large number of avian-affiliated ichnogenera could be eliminated on the basis of gross morphology. For example, none of the tracks are zygodactyl, nor are any fully palmate with distal webbing, which are forms expressed in Cretaceous bird tracks elsewhere [52, 61–64]. We accordingly evaluated possible ichnogenera by first placing the Wonthaggi specimens into broad anatomical categories of: anisodactyl incumbent with proximal webbing; anisodactyl incumbent without proximal webbing; and anisodactyl without webbing. A few tracks, such as FF-4-1, FF-4-3 and FF-5B-3, still do not correspond with ichnogenera based on known bird-track morphotypes, but the majority are at least comparable to some described outside of Australia (Table 2).

Table 2. Avian-affiliated ichnogenera that might be applied to Wonthaggi Formation tracks. See text and S2 for descriptions, depictions, and diagnoses of individual tracks.

Ichnogenus	Specimens
Aquatilavipes	HB-1, FF-1-1, FF-1-2, FF-5A-1
Ardeipeda	FF-1-3, FF-1-4
Avipeda	FF-2-1, FF-3-1, FF-3-5, FF-4-2, FF-5A-2
Fuscinapeda	FF-3-2, FF-3-3, FF-4-4, FF-4-5, FF-4-7, FF-5A-3, FF-5A-4
Gyeongsangornipes	FF-2-1, FF-3-1, FF-3-5, FF-4-2, FF-5A-2
Hwangsanipes	FF-5B-2, FF-5B-4
Limiavipes	5A-5
Wupus	FF-3-6, FF-4-3, FF-4-6, FF-5B-1

Tracks expressed as anisodactyl incumbent with proximal webbing, such as FF-2-1, FF-3-1, FF-3-5, FF-4-2 and FF-5A-2, somewhat resemble *Gyeongsangornipes* from Early Cretaceous strata of Korea [65; 54; 66], and *Uhangrichnus* from Late Cretaceous strata of Korea and Alaska [67; 68]. However, the Wonthaggi specimens do not exactly match these ichnogenera, as *Gyeongsangornipes* is smaller, has asymmetrical web impressions between digits II-IV, and a higher divarication (~125-130°) between digits II-IV [54; 56; 64]. *Uhangrichnus* is even smaller than the Wonthaggi tracks and has equally developed web impressions between digits II-IV, approaching fully palmate [67; 56]. A better candidate for these Wonthaggi tracks is *Avipeda*, an ichnogenus that is typically applied to Cenozoic avian tracks [21; 64]. For *Avipeda*, tracks are anisodactyl incumbent, digits are shorter and relatively thicker, and webbing is either lacking or limited to the most proximal parts of interdigital angles [69; 70]. However, the Wonthaggi tracks also differ slightly from *Avipeda* in size and divarication. In short, we note that anisodactyl incumbent tracks with proximal webbing are similar to *Avipeda*, but may not entirely fit that ichnogenus, either.

Other Wonthaggi tracks such as FF-3-2, FF-3-3, FF-4-4, FF-4-5, FF-4-7, FF-5A-3 and FF-5A-4, resemble the Cenozoic ichnogenus *Fuscinapeda*, which some researchers have placed in the “ichnofamily” Avipedidae [69; 71; 27; 21]. Such tracks are anisodactyl incumbent and relatively larger than similar ichnogenera, with slim to moderately thick digits, a divarication of about 105°, and webbing either confined to the most proximal parts of interdigital angles or absent [70; 27]. Nonetheless, the Wonthaggi tracks also have a few morphological differences from previously known specimens of *Fuscinapeda*, such as greater size and divarication. Thus we can only suggest that the avian tracemakers of these specimens had ecomorphological affinities to this ichnogenus.

Wonthaggi tracks that were anisodactyl incumbent but without obvious proximal webbing are similar to a few previously reported Cretaceous avian ichnogenera. Among these are HB-1, FF-1-1, FF-1-2 and FF-5A-1, which align with the ichnogenus *Aquatilavipes* and other ichnogenera from the "ichnofamily" Avipedidae [21, 64, 72–74]. Still, lengths and widths of the Wonthaggi specimens are greater than most *Aquatilavipes,* although size differences in this ichnogenus are reported in other deposits [75]. Wonthaggi track FF-5A-5 is also morphologically similar to the ichnogenus *Limiavipes* ("ichnofamily" Limiavipedidae), but its length and width are slightly smaller than others identified from elsewhere [76]. Some anisodactyl incumbent tracks, such as FF-3-6, FF-4-3, FF-4-6, and FF-5B-1, are probably assignable to the ichnogenus *Wupus* ("Ichnofamily" Limiavipedidae), which is likewise an asymmetrical and larger track with a sub-rounded metatarsal impression [21, 27], a feature evident in tracks FF-4-3 and FF-4-5. *Wupus* was originally interpreted as belonging to nonavian theropods, but was later credited to avians [27, 77), affirmed by Lockley and others [21]. Diagnostic features of *Wupus* are its relatively large size (10-11.5 cm long), length:width ratios of 0.7-0.9, digit II-IV divarication angles averaging about 98°, webbing either indistinct or absent, and no hallux [21, 27]. These criteria match at least three of the specimens from Footprint Flats, FF-3-6, FF-4-3, FF-4-6. *Aquatilavipes*, *Limiavipes*, and *Wupus* have been identified in Early Cretaceous strata of eastern Asia and North America, but never before in Gondwanan strata.

Wonthaggi tracks that are anisodactyl (digits I-IV present) but with no webbing evident, such as FF-1-3 and FF-1-4, are similar to the Cenozoic ichnogenus *Ardeipeda*, which resembles tracks of modern long-legged wading birds (e.g., *Ardea alba*, *A. herodias*). Such tracks have three thin forward-pointing digits and a well-developed hallux pointing nearly opposite of those impressions. Ichnospecies reported for this ichnogenus include *A. egretta* and *A. gigantea* [63,

78]. Wonthaggi tracks that are anisodactyl (digits I-IV present) and with proximal webbing, such as FF-5B-2 and FF-5B-4, closely resemble the ichnogenus *Hwangsanipes* (“ichnofamily” Ignotornidae), which also has a long posteromedially oriented hallux and asymmetrical web impressions between digits II-III and III-IV [55, 66–67]. However, the lengths and widths of FF-5B-2, FF-5B-2, and FF-5B-4 are greater than those of previously known examples of *Hwangsanipes*.

Several Wonthaggi tracks (e.g., FF-1-4) have possible hallux imprints, but distorted sediment in the rear part of the track obscures its presence. In a similar vein, sedimentary structures formed as parts of footprints, such as cohesive mounds and wedges of sediment around track interiors, may result in false proximal or distal webbing between digits [79]. With this factor in mind, we cannot reliably interpret proximal webbing between digits II-III or III-IV, or semipalmated feet, from the Wonthaggi tracks. Thus we prefer to suggest that a few tracks show evidence of proximal webbing, which would accordingly affect their naming.

Ichnotaxonomic conventions aside, the Wonthaggi avian-track assemblage represent an unprecedented variety and number of Early Cretaceous avian tracks from the Southern Hemisphere. Although ichnodiversity does not necessarily equal biodiversity, the morphological and size variations of the Wonthaggi tracks (Fig. 13) imply they were made by multiple species of birds during the Early Cretaceous in this part of Australia. Moreover, some of the Wonthaggi specimens, such as FF-4-3, FF-4-6, and FF-4-7, are among the largest known avian tracks reported from the Early Cretaceous [21]. Although some ichnogenera identified here are only “best fits” for now, and the naming of new ichnotaxa may be warranted in the future, we must emphasize that such nomenclatural quibbles will not change our base interpretation of the avian diversity represented by these footprints in this time and place.

Paleontological and Paleoecological Significance of the Wonthaggi Tracks

The only undoubted avian body fossils from the Wonthaggi Formation include a flight feather [11] and a furcula; the latter was identified as that of an enantiornithine [8]. However, most Wonthaggi tracks more closely resemble those of ornithurines, an identity Martin and others (2014) suggested for two bird tracks from the Eumeralla Formation at Dinosaur Cove. The greater age of the Wonthaggi tracks (Barremian-Aptian) versus the Eumeralla tracks (Albian) nonetheless implies the former are more likely from enantiornithean birds. The oldest body fossils of ornithurines are from China (*Archaeornithura*, *Protopteryx*), which are Hauterivian, whereas the oldest enantiornithine other than *Archaeopteryx* is *Noguerornis* from the earliest Barremian of Spain, placing these taxa at about 131-129 *Ma* [80–82]. As mentioned previously, the oldest named enantiornithine in Gondwana is *Cratoavis cearensis* from the Crato Formation (Aptian) of Brazil [12–13]. The greater age of the Wonthaggi tracks would thus extend the probable record of enantiornithines to earlier in the Cretaceous, and closer to the oldest known birds outside of Gondwana.

This variety of avian tracks in formerly circumpolar Early Cretaceous environments is also paleoecologically significant, suggesting a diverse suite of birds living in these ecosystems between 120-128 *Ma*. Two avian footprints from the geologically younger (Albian) Eumeralla Formation at Dinosaur Cove, dating at about 106 *Ma* [9], further argue for an avian presence in circumpolar ecosystems of Australia for about 15-20 million years. Circumpolar avian tracks are documented in the Northern Hemisphere from Alaska [68, 83], but these are also geologically younger (Campanian-Maastrichtian) than both the Eumeralla and Wonthaggi tracks. In short, the

Wonthaggi tracks not only represent the oldest documented bird-track assemblage in Australia and Gondwana, but also the oldest from formerly polar environments.

This presence of birds on rippled sandy floodplain surfaces – presumably during a circumpolar spring or summer – also prompts a query of why they were there. Small invertebrate burrows in the same strata preserving avian tracks provide one answer (Figure 16), as this infauna would have provided food for birds foraging in these environments. Such behaviors have been interpreted from bird tracks and feeding sign in Early Cretaceous rocks of Korea [84]. Bird foraging as flocks on floodplains also may have resulted in similarly oriented tracks, such as those measured on strata 3 and 5 (Figure 15). Furthermore, some tracks, such as FF-5A-3, FF-5A-4, FF-5B-2, and FF-5B-4, have prominent sedimentary structures that imply sudden stops, perhaps indicating landing from either a hop or flight. Flighted behavior was proposed for producing similar features in one of the two avian footprints from the Early Cretaceous Eumeralla Formation (9), and from Early Cretaceous bird tracks of Korea [84].

Regardless of paleolatitude, bird trace fossils are either rare or rarely recognized from Cretaceous strata of former Gondwanan landmasses, and are not yet verified from the Late Jurassic, either. Other than tracks from the Eumeralla Formation (Albian) of Australia [9] and this study, Gondwanan bird tracks have only been reported from Late Cretaceous strata, such as in the Anacleto Formation (Campanian) and Yacoraite Formation (Maastrichtian) of Argentina [14–17], as well as in the Zebbag Formation (Cenomanian) of Tunisia [18]. Late Triassic-Early Jurassic avian-like footprints were interpreted by Ellenberger [85–86] in South Africa, as well as in Argentina [87]. However, the South African tracks are more likely those of non-avian theropods, whereas the Argentine tracks are avian, but were later dated as Late Eocene [88–89].

As a result, Triassic or Jurassic avian footprints are so far unconfirmed from Gondwanan landmasses.

In contrast to Gondwana, Early and Late Cretaceous bird tracks are abundant and diverse in former Laurasian landmasses, such as North America [64, 68, 72, 75, 83, 90] and Asia [27–28, 52–53, 55–60, 73, 91], including those in Early Cretaceous strata [27, 52, 55–59, 60, 62, 74, 92]. The origin of birds is currently hypothesized to have happened during the Middle to Late Jurassic, and in Laurasia [93, 94]. Hence the greater numbers and varieties of Cretaceous bird tracks in Laurasia may reflect proximity to their paleogeographic origin. As of now, we do not know if the uneven distribution of Cretaceous bird tracks in Laurasia and Gondwana is related to: the timing of bird origins and their subsequent evolution and dispersal; preservation bias; researchers not recognizing avian tracks in Gondwanan strata; or a combination of these factors. We nevertheless hope the results of our study provides impetus for researchers to seek more evidence of Early Cretaceous birds in former Gondwanan landmasses, and in particular via their trace fossils.

A paleoecologically significant facet of the Wonthaggi Formation bird tracks is their presence in formerly circumpolar environments during the Early Cretaceous. Their occurrence on fluvial floodplains also implies seasonally linked behaviors, in which they would have walked across emergent surfaces after flooding from spring thaws. Martin [24] similarly concluded that modern avian tracks and other vertebrate tracks on an Arctic point bar were more likely made and preserved during summer, rather than other seasons. The recurrence of avian tracks on at least five stratigraphic levels at Footprint Flats also hints at multiple seasonal behaviors that allowed birds to gather on exposed fluvial floodplains, which also aided in the formation and preservation of their tracks.

In summary, through a combination of avian body fossil evidence in the Wonthaggi Formation [8, 10–11] and trace fossils in the Wonthaggi and Eumeralla Formations [9], we propose that a variety of birds were participating members of polar-ecosystem communities in Australia for at least 15-20 million years during the Early Cretaceous. We anticipate that this ichnologically aided revelation will contribute to and spur on further inquiries about the global dispersal of birds since their origin from non-avian theropods during the Jurassic [93, 94].

Conclusions

- Tracks in the Wonthaggi Formation (Barremian-Aptian, 128-120 *Ma*) of Victoria, Australia are the earliest known trace fossils attributed to birds in Australia, while also supplementing rare body fossil evidence of birds in the same formation.
- The Wonthaggi avian tracks are also the oldest documented from former Gondwanan landmasses and the oldest from formerly polar environments.
- Based on track forms and sizes, the avian trackmakers were varied, including birds larger than most other Early Cretaceous examples, and with sizes overlapping those of non-avian theropods.
- The Wonthaggi avian tracks occurring on multiple stratigraphic levels indicate a variety of birds on circumpolar floodplains of southern Australia more than 120 *Ma*, and their recurrent presence in those environments suggest seasonal formation of these traces during polar summers.

Supporting information

S1 – File. Description of molding and casting process for avian tracks in Wonthaggi Formation.
(DOCX)

S2 – File. Detailed descriptions of individual tracks from the Wonthaggi Formation augmented by labeled photos and line drawings.
(PDF)

Acknowledgments

The research was conducted on the ancestral lands of the Bunurong people, and we appreciate the guidance of Dan Turnbull in advising us of respectful practices for studying the paleontological legacy of these places. We are grateful to the Royal Auto Club of Victoria (RACV) for providing discounted lodging for most of us during field work there in May 2022. Partial support for travel and field expenses was provided by our respective institutions. Field work was assisted by Aidan Lowery, Ruth Schowalter, Doris Seeget-Villers, Lesley Kool, and Mike Cleeland. We thank the editor and reviewers for their helpful suggestions to improve the manuscript.

References

1. Murray PF, Vickers-Rich P. Magnificent mihirungs: The colossal flightless birds of the Australian dreamtime. Bloomington, Indiana: Indiana University Press; 2004.

2. Mayr G. Two-phase extinction of "Southern Hemispheric" birds in the Cenozoic of Europe and the origin of the Neotropic avifauna. Palaeobiodiversity and Palaeoenvironments 2011;91: 325–333.

3. Jetz W, Thomas G, Joy J, Hartman K, Mooers AO. The global diversity of birds in space and time. Nature 2012;491: 444–448.

4. Worthy TH, Nguyen JMT. An annotated checklist of the fossil birds of Australia. Transactions of the Royal Society of South Australia 2020;144: 66-108.

5. Molnar RE. An enantiornithine bird from the Lower Cretaceous of Queensland, Australia. Nature 1986;322: 736-738.

6. Kurochkin EN, Molnar, RE. New material of enantiornithine birds from the Early Cretaceous of Australia. Alcheringa 1997;21: 291-297.

7. Molnar RE. Avian tibiotarsi from the Early Cretaceous of Lightning Ridge, New South Wales. In: Tomida, Y, Rich, TH, Vickers-Rich, P., editors. Proceedings of the Second Gondwanan Dinosaur Symposium, Tokyo; 1992. pp. 197–209.

8. Close RA, Vickers-Rich P, Trusler P, Chiappe LM, O'Connor J, Rich, TH, Komarower, P. Earliest Gondwanan bird from the Cretaceous of southeastern Australia. Journal of Vertebrate Paleontology, 2009;29: 616-619.

9. Martin AJ, Vickers-Rich P, Rich, TH, Hall, M. Oldest known avian footprints from Australia: Eumeralla Formation (Albian), Dinosaur Cove, Victoria. Palaeontology 2014;57: 7-19.

10. Poropat SF, Martin SK, Tosolini A-MP, Wagstaff BE, Bean LB, Kear BP. 2018. Early Cretaceous polar biotas of Victoria, southeastern Australia—an overview of research to date. Alcheringa 2018;42: 157-229.

11. Kundrát M, Rich, TH, Lindgren J, Sjövall P, Vickers-Rich P, Chiappe, LM. A polar dinosaur feather assemblage from Australia. Gondwana Research 2020;80: 1-11.

12. Carvalho IS, Novas FE, Agnolín FL, Isasi MP, Freitas FI, Andrade JA. A new genus and species of enantiornithine bird from the Early Cretaceous of Brazil. Brazilian Journal of Geology 2015;45: 161–171.

13. Carvalho IS, Novas FE, Agnolín FL, Isasi MP, Freitas FI, Andrade JA. A Mesozoic bird from Gondwana preserving feathers. Nature Communications 2015;6: 7141.

14. Alonso RN, Marquillas RA. Nueva localidad con huellas de dinosaurios y primer hallazgo de huellas de aves en la Formaci´on Yacoraite (Maastrichtiano) del norte argentino. 4th Congreso Argentino de Paleontología y Bioestratigrafía, Actas 1986;2: 33–42.

15. Coria RA, Currie PJ, Eberth D, Garrido, R. Bird footprints from the Anacleto Formation (Late Cretaceous) in Neuquén Province, Argentina. *Ameghiniana* 2002;39: 453-463.

16. de Valais S, Cónsole-Gonella C, I. Díaz-Martínez, I. Registro de huellas avianas en la Formación Yacoraite (Maastrichtiano-Daniano), NOA, Argentina. Ameghiniana 2016:53: 15.

17. de Valais S, Cónsole-Gonella C. An updated review of the avian footprint record from the Yacoraite Formation (Maastrichtian-Danian), northwestern Argentina. Ichnos 2019:26: 224–241.

18. Contessi M, Fanti, F. First record of bird tracks in the Late Cretaceous (Cenomanian) of Tunisia. Palaios 2012;27: 455-464.

19. Rich TH, Vickers-Rich P, Gangloff RA. Polar dinosaurs. Science 2002;295: 979–980.

20. Rich TH, Vickers-Rich P. Dinosaurs of darkness, 2nd ed. Bloomington, Indiana: Indiana University Press; 2020.

21. Lockley MG, Abbassi N, Helm CW. Large, unwebbed bird and bird- like footprints from the Mesozoic and Cenozoic: a review of ichnotaxonomy and trackmaker affinity. Lethaia 2021;54: 969-987.

22. Gatesy, SM, Middleton KM, Jenkins FA, Shubin NH. Three-dimensional preservation of foot movements in Triassic theropod dinosaurs. Nature 1999;399: 141–144.

23. Gatesy SM. Direct and indirect track features: what sediment did a dinosaur touch? Ichnos 2003;10: 91–98.

24. Martin AJ. Neoichnology of an Arctic fluvial point bar, Colville River, Alaska (USA). Geological Quarterly 2009;53: 383-396.

25. Lockley MG, Yang SY, Matsukawa M, Fleming F, Lim, SK. The track record of Mesozoic birds: evidence and implications. Philosophical Transactions of the Royal Society of London, 1992;336: 113–134.

26. Falk AR, Martin LD, Hasiotis ST. A morphologic criterion to distinguish bird tracks. Journal of Ornithology 2011;152: 701-716.

27. Xing L, Buckley LG, McCrea RT, Lockley MG, Zhang J, Piñuela L. Reanalysis of *Wupus agilis* (Early Cretaceous) of Chongqing, China as a large avian trace: differentiating between large bird and small non-avian theropod tracks. PLOS ONE 2015;10(5): e0124039.

28. Imai T, Tsukiji Y, Azuma Y. Description of bird tracks from the Kitdani Formation (Aptian), Katsuyama, Fukui, Japan with three-dimensional imaging techniques. Memoir of the Fukui Prefectural Dinosaur Museum 2018: 17: 1–8.

29. Falkingham PL, Bates KT, Avanzini M, Bennett M, Bordy E, Breithaupt BH, Castanera D, et al. A standard protocol for documenting modern and fossil ichnological data. Palaeontology 2018;61: 469–480.

30. Wagstaff BE, Gallagher SJ, Hall M, Korasidis VA, Rich TH, Seegets-Villiers DE, Vickers-Rich P. Palynological-age determination of Early Cretaceous vertebrate-bearing beds along the south Victorian coast of Australia, with implications for the spore-pollen biostratigraphy of the region. Alcheringa 2020;44:3, 460-474.

31. Veevers JJ, Powell CM, Roots SB. Review of seafloor spreading around Australia, I. Synthesis of the patterns of spreading. Australian Journal of Earth Sciences 1991;38: 373–389.

32. Bryan SE, Constantine AE, Stephens CJ, Ewart A, Schöen RW, Parianos J. Early Cretaceous volcano-sedimentary successions along the eastern Australian continental margin: implications for the break-up of eastern Gondwana. Earth and Planetary Science Letters 1997; 153: 85–102.

33. Tosolini A-MP, McLoughlin S, Drinnan AN. Stratigraphy and fluvial sedimentary facies of the Neocomian lower Strzelecki Group, Gippsland Basin, Victoria. Australian Journal of Earth Sciences 1999;46: 951–970.

34. Aghaei H, Hall M, Wagstaff B, Tait A. Stratigraphic reconstruction of the Strzelecki Group outcrops in west Gippsland: new data on the present-day thickness and amount of erosion. Australian Journal of Earth Sciences 2017;64(2): 251-264.

35. Alley NF, Hore SB, Frakes LA. Glaciations at high-latitude Southern Australia during the Early Cretaceous. Australian Journal of Earth Science 2020;6: 1045–1095.

36. Hore SB, Hill SM, Alley NF. Early Cretaceous glacial environment and paleosurface evolution within the Mount Painter Inlier, northern Flinders Ranges, South Australia. Australian Journal of Earth Sciences 2020;67: 1117–1160.

37. Rich PV, Rich TH, Wagstaff BE, McEwen-Mason J, Douthitt CB, Gregory, RT. Evidence for low temperatures and biologic diversity in Cretaceous high latitudes of Australia. Science 1988;242: 1403–1406.

38. Gregory RT, Douthitt CB, Duddy IR, Vickers-Rich P, Rich, TH. Oxygen isotopic composition of carbonate concretions from the lower Cretaceous of Victoria, Australia: implications for the evolution of meteoric waters on the Australian continent in a paleopolar environment. Earth and Planetary Science Letters 1989;92, 27–42.

39. Constantine AE, Chinsamay A, Vickers-Rich P, Rich TH. Periglacial environments and polar dinosaurs. South African Journal of Science 1998;94: 137–141.

40. Veevers JJ. Updated Gondwana (Permian–Cretaceous) earth history of Australia. Gondwana Research 2006;9, 231–260.

41. Tosolini AMP, Korasidisa VA, Wagstaff BE, Cantrill DJ, Gallagher SJ, Norvick MS. Palaeoenvironments and palaeocommunities from Lower Cretaceous high-latitude sites, Otway Basin, southeastern Australia. Palaeogeography, Palaeoclimatology, Palaeoecology 2018;496: 62–84.

42. Rich, TH, Vickers-Rich, P. 1999. The Hypsilophodontidae from southeastern Australia. In: Tomada, Y, Rich, TH, and Vickers-Rich, P., editors. Proceedings of the Second Gondwana Dinosaur Symposium. National Science Museum Monographs 15; 1999. pp. 167–180

43. Wagstaff BE, Gallagher SJ, Norvick M, Cantrill DJ, Wallace MW. High latitude Albian climate variability: palynological evidence for long-term drying in a greenhouse world. Palaeogeography, Palaeoclimatology, Palaeoecology 2013;386: 501–511.

44. Tosolini A-MP, McLoughlin S, Wagstaff BE, Cantrill DJ, Gallagher SJ. Cheirolepidiacean foliage and pollen from Cretaceous high-latitudes of southeastern Australia. Gondwana Research 2015;26: 960–977.

45. Elbroch M, Marks E. 2001. Bird tracks and sign: A guide to North American species. Mechanicsburg, Pennsylvania, Stackpole Books; 2001.

46. Lockley, MG. New perspectives on morphological variation in tridactyl footprints: clues to widespread convergence in developmental dynamics. Geological Quarterly 2009;53: 415–432.

47. Wright JL. Bird-like features of dinosaur footprints. In: Currie PJ, Koppelhus EB, Shugar MA, Wright JL., editors. Feathered dragons: Studies on the transition from dinosaurs to birds. Bloomington, Indiana: Indiana University Press; 2006. pp. 176-184.

48. Lockley, MG. New perspectives on morphological variation in tridactyl footprints: clues to widespread convergence in developmental dynamics. Geological Quarterly 2009;53: 415–432.

49. Martin AJ. Life traces of the Georgia coast: Revealing the unseen lives of plants and animals. Bloomington, Indiana: Indiana University Press; 2013.

50. Gatesy SM, Falkingham, PL. Neither bones nor feet: track morphological variation and 'preservation quality.' Journal of Vertebrate Paleontology 2017;37:3. doi: 10.1080/02724634.2017.1314298

51. Falk AR, Hasiotis ST, Gong E, Kim J-D, Brewer ED. A new experimental setup for studying avian neoichnology and the effects of grain size and moisture content on tracks: trails using the domestic chicken (*Gallus gallus*). Palaios 2017;32: 689-707.

52. Lim J-D, Zhou Z, Martin LD, Baek K-S, Yang, S-Y. The oldest known tracks of web-footed birds from the Lower Cretaceous of South Korea. Naturwissenschaften 2000;87: 256–259.

53. Kim JY, Lockley MG, Seo SJ, Kim KS, Kim SH, Seok Baek, KS. A paradise of Mesozoic birds: the world's richest and most diverse Cretaceous bird track assemblage from the Early Cretaceous Haman Formation of the Gajin tracksite, Jinju, Korea. Ichnos 2012:19; 28-42.

54. Kim JY, Kim MK, Oh MS, Lee, CZ. A new semi-palmate bird track, *Gyeongsangornipes lockleyi* ichnogen. et ichnosp. nov., and *Koreanaornis* from the Early Cretaceous Jindong Formation of Goseong County, southern coast of Korea. Ichnos 2013;20: 72-80.

55. Lockley MG, Lim J-D, Kim JY, Kim KS, Huh M, Hwang K-G. Tracking Korea's early birds: a review of Cretaceous avian ichnology and its implications for evolution and behavior. Ichnos 2012;19: 17-27.

56. Xing L, Buckley LG, Lockley MG., Zhang J, Marty D, Wang Y. A new bird track, *Koreanaornis lii* ichnosp. nov., from the Lower Cretaceous Hekou Group in the Lanzhou-Minhe Basin, Gansu, northwest China, and implications for Early Cretaceous avian diversity. Cretaceous Research 2016;66: 141-154.

57. Xing L, Chou C, Lockley MG, Yangchen D, Chen X, Ma, X. First report of avian tracks from the Cretaceous of Tibet, China. Acta Geologica Sinica 2017;91(6): 2312–2313.

58. Xing L, Hu Y-C, Huang J-D, He Q, Lockley MG, Burns ME. A redescription of the ichnospecies *Koreanaornis anhuiensis* (Aves) from the Lower Cretaceous Qiuzhuang Formation at Mingguang city, Anhui Province, China. Journal of Palaeogeography 2018;7: 58e65.

59. Xing L, Lockley MG, Zhang J, Romilio A, Klein H, Wang Y. A diversified vertebrate ichnite fauna from the Dasheng Group (Lower Cretaceous) of southeast Shandong Province, China. Historical Biology 2019;31: 353–362.

60. Xing L, Lockley MG, Jia C, Klein H, Niu K, Zhang L. Lower Cretaceous avian-dominated, theropod, thyreophoran, pterosaur and turtle track assemblages from the Tugulu Group, Xinjiang, China: ichnotaxonomy and palaeoecology. PeerJ 2021;9: e11476.

61. Li R, Lockley MG, Liu, M. A new ichnotaxon of fossil bird track from the Early Cretaceous Tianjialou Formation (Barremian-Albian), Shandong Province, China. Chinese Science Bulletin 2005;50: 1149e1154.

62. Kim JY, Kim SH, Kim KS, Lockley M. The oldest record of webbed bird and pterosaur tracks from South Korea (Cretaceous Haman Formation, Changseon and Sinsu Islands): more evidence of high avian diversity in East Asia. Cretaceous Research 2006:27; 56–69.

63. Lockley MG, Li R, Harris, JD, Matsukawa M, Liu M. Earliest zygodactyl bird feet: evidence from Early Cretaceous roadrunner-like tracks. Naturwissenschaften 2007;94: 657-665.

64. Serrano-Brañas CI, Espinosa-Chávez B, Ventura JF, Barrera-Guevara D, Torres-Rodríguez, E, Vega FJ. New insights on the avian trace fossil record from NE Mexico: Evidences on the diversity of latest Maastrichtian web-footed bird tracks. Journal of South American Earth Sciences 2022;113: 103686.

65. Lockley, MG, Matsukawa, M, Ohira, H, Li, J, Wright, J, White, D, Chen, P, Bird tracks from Liaoning Province, China: new insights into avian evolution during the Jurassic-Cretaceous transition. Cretaceous Research 2006;27: 33–43.

66. Kim JY, Huh M. Birds from the Cretaceous of Korea. In: Kim JY, Huh M, editors. Dinosaurs, birds, and pterosaurs of Korea. Singapore: Springer; 2018. pp. 109-137.

67. Yang SY, Lockley MG, Greben R, Erickson BR, Lim SK. Flamingo and duck-like traces from the Late Cretaceous and early Tertiary: evidence and implications. Ichnos 1995; 4: 21–34.

68. Fiorillo AR, Hasiotis ST, Kobayashi Y, Breithaupt BH, McCarthy, PJ. Bird tracks from the Upper Cretaceous Cantwell Formation of Denali National Park, Alaska, USA: a new perspective on ancient northern polar vertebrate biodiversity. Journal of Systematic Palaeontology 2011;9(1): 33-49.

69. Sarjeant WAS, Langston, W. Vertebrate footprints and invertebrate traces from the Chadronian (Late Eocene) of Trans-Pecos Texas. Bulletin of Texas Memorial Museum 1994;36: 1-86.

70. Sarjeant WAS, Reynolds RE. Bird Footprints from the Miocene of California. In: Reynolds RE, editor. The Changing Face of the East Mojave Desert. 2001 Desert Symposium, Fullerton, California. California State University; 2001. pp. 21–40.

71. Lockley MG, Harris JD. On the trail of early birds: A review of the fossil footprint record of avian morphological and behavioral evolution. In: Ulrich PK, Willett JH, editors. Trends in Ornithology Research. Hauppauge, New York: Nova Science Publishers Inc.; 2010. pp. 1-63.

72. Currie PJ. Bird footprints from the Gething Formation (Aptian, Lower Cretaceous) of northeastern British Columbia, Canada. Journal of Vertebrate Paleontology 1984;1: 257-264.

73. Azuma Y, Arakawa Y, Tomida Y, Currie PJ. Early Cretaceous bird tracks from the Tetori Group, Fukui Prefecture, Japan. Memoir of the Fukui Prefectural Dinosaur Museum 2002; 1: 1-6.

74. Huh M, Lockley, MG, Kim, KS, Kim, JY. First report of *Aquatilavipes* from Korea: New finds from Cretaceous strata in the Yeosu Islands Archipelago. Ichnos 2012;19: 43–49.

75. Lockley MG, Buckley LG, Foster JR, Kirkland JI, DeBlieux, DD. First report of bird tracks (*Aquatilavipes*) from the Cedar Mountain Formation (Lower Cretaceous), eastern Utah. Palaeogeography, Palaeoclimatology, Palaeoecology 2015;420: 150–162.

76. McCrea, RT, Buckley, LG, Plint, AG, Currie, PJ, Haggart, JW, Helm, CW, Pemberton, SG, A review of vertebrate track-bearing formations from the Mesozoic and Earliest Cenozoic of western Canada with a description of a new theropod ichnospecies and reassignment of an avian ichnogenus. In: Lockley, MG, Lucas, SG, editors, Fossil footprints of western North America. New Mexico Museum of Natural History and Science Bulletin, 2014: 62;5–94.

77. Xing, LD, Wang, FP, Pan, SG, Chen, W. The discovery of dinosaur footprints from the Middle Cretaceous Jiaguan Formation of Qijiang County, Chongqing City. Acta Geologica Sinica 2007;81: 1591–1602. (In Chinese with English abstract.)

78. Panin, N, Avram, E. Noi orme de vertebrate in Miocenul Subcarpatilor Rominesti. Studii si Cercetari de Geologie, Geofizica, Geografie 1962;7: 455-484.

79. Falkingham PL, Margetts L, Smith IA, Manning PL. Reinterpretation of palmate and semi-palmate (webbed) fossil tracks; insights from finite element modeling. Palaeogeography, Palaeoclimatology, Palaeoecology 2009;271: 69–76.

80. Zhang F, Zhou Z. A primitive enantiornithine bird and the origin of feathers. Science 2000; 290: 1955–1959.

81. Chiappe LM, Lacasa-Ruiz A. *Noguerornis gonzalezi* (Aves: Ornithothoraces) from the Early Cretaceous of Spain. In: Chiappe LM, Witmer L.M., editors. Mesozoic birds: above the heads of dinosaurs. Berkeley, California, University of California Press; 2002. pp. 230-239.

82. Wang M, Zheng X, O'Connor J, Lloyd GT, Wang X, Wang Y, Zhang X., Zhou, Z. The oldest record of Ornithuromorpha from the Early Cretaceous of China. Nature Communications 2015;6: 6987. Doi: https://doi.org/10.1038/ncomms7987.

83. Fiorillo AR, Kobayashi Y, McCarthy PJ, Tanaka T, Tykoski RS, Lee Y-N. Dinosaur ichnology and sedimentology of the Chignik Formation (Upper Cretaceous), Aniakchak National Monument, southwestern Alaska; Further insights on habitat preferences of high-latitude hadrosaurs. PLOS ONE 2009;14(10): e0223471.

84. Falk AR, Lim J-D, Hasiotis ST. A behavioral analysis of fossil bird tracks from the Haman Formation, Republic of Korea shows a nearly modern avian ecosystem. Vertebrata Palasiatica 2014;52: 129-152.

85. Ellenberger P. Contribution 'a la classification des pistes de vertebres du Trias: les types du Stormberg d'Afrique du Sud (I). Palaeovertebrata, Memoire Extraordinaire, 1972. pp. 1-111.

86. Ellenberger P. Contribution ‘a la classification des pistes de vertebres du Trias: les types du Stormberg d’Afrique du Sud (IIe‘me partie: le Stormberg Superieur -I. Le biome de la zone B/1 ouniveau de Moyeni: ses biocenoses). Palaeovertebrata, Memoire Extraordinaire 1974; 1-143.

87. de Valais S, Melchor RN. Ichnotaxonomy of bird-like footprints: an example from the Late Triassic-Early Jurassic of northwest Argentina. Journal of Vertebrate Paleontology 2008;28: 145–159.

88. Genise JF, Melchor RN, Archangelsky M, Bala LO, Straneck R, de Valais S. Application of neoichnological studies to behavioural and taphonomic interpretation of fossil bird-like tracks from lacustrine settings: the Late Triassic-Early Jurassic? Santo Domingo Formation, Argentina. Palaeogeography, Palaeoclimatology, Palaeoecology 2009; 272: 143–161.

89. Melchor RM, Buchwaldt R, Bowring S. A Late Eocene date for Late Triassic bird tracks. Nature 2013;495: E1–E2.

90. Buckley LG, McCrea RT, Xing L. First report of Ignotornidae (Aves) from the Lower Cretaceous Gates Formation (Albian) of western Canada, with description of a new ichnospecies of *Ignotornis, Ignotornis canadensis* ichnosp. nov. Cretaceous Research 2018;84: 209-222.

91. Azuma Y, Lu J, Jin X, Noda, Y., Shibata, M., Chen, R. A bird footprint assemblage of early Late Cretaceous age, Dongyang City, Zhejiang Province, China. *Cretaceous Research* 2013;40: 3-9.

92. Xing L, Lockley MG, Zhang J, Romilio A, Klein H, Wang Y. A diversified vertebrate ichnite fauna from the Dasheng Group (Lower Cretaceous) of southeast Shandong Province, China. Historical Biology 2019;31: 353–362.

93. Xu X, You H, Du K, Han, F. An *Archaeopteryx*-like theropod from China and the origin of Aviale. Nature, 2011;475: 465–470.

94. Lloyd GT, Bapst DW, Friedman M, Davis KE. Probabilistic divergence time estimation without branch lengths: dating the origins of dinosaurs, avian flight and crown birds. Biology Letters 2016;12: 0160609.

Figure 1

Click here to access/download;Figure;Fig1.tif

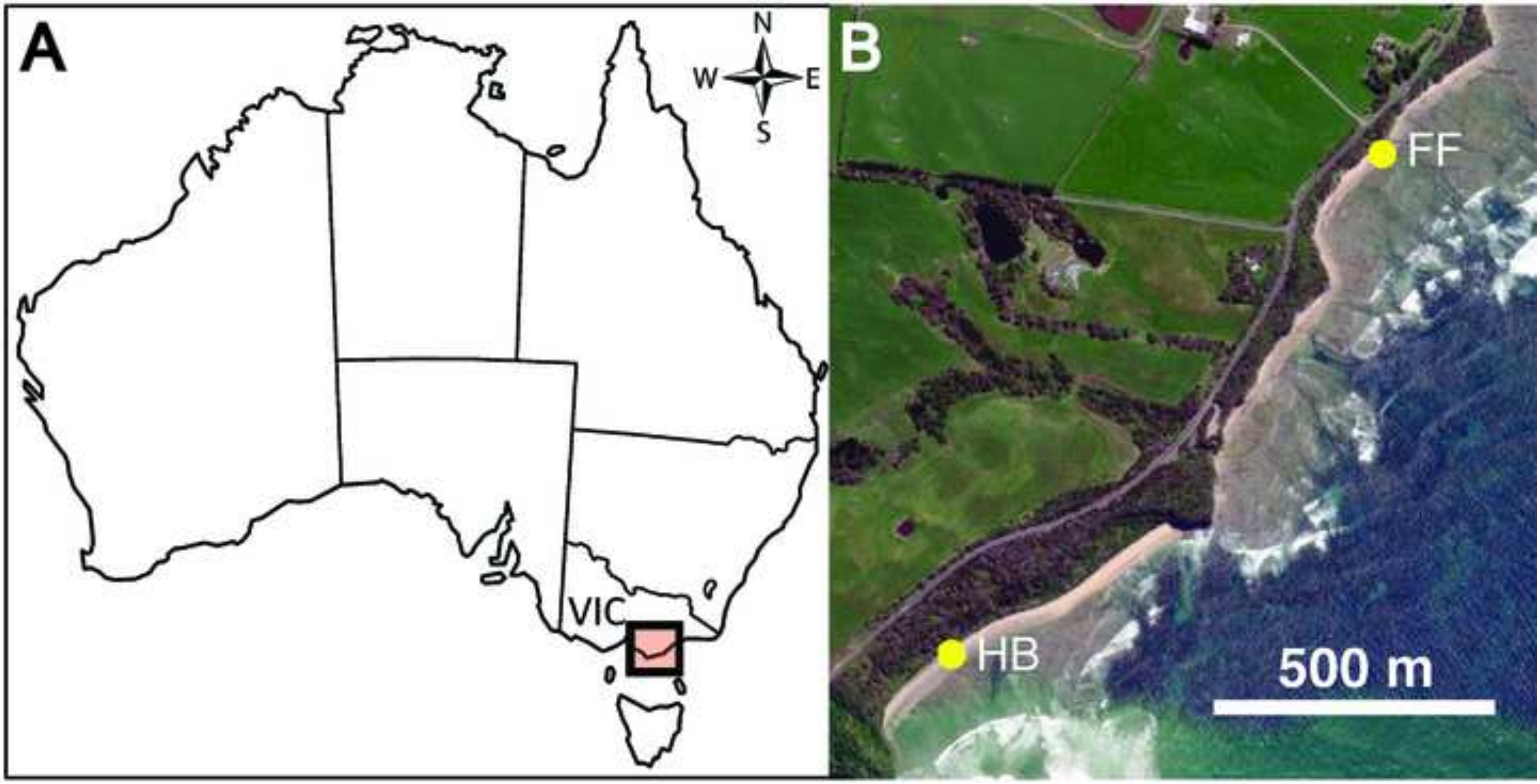

 Click here to access/download;Figure;Fig2.tif

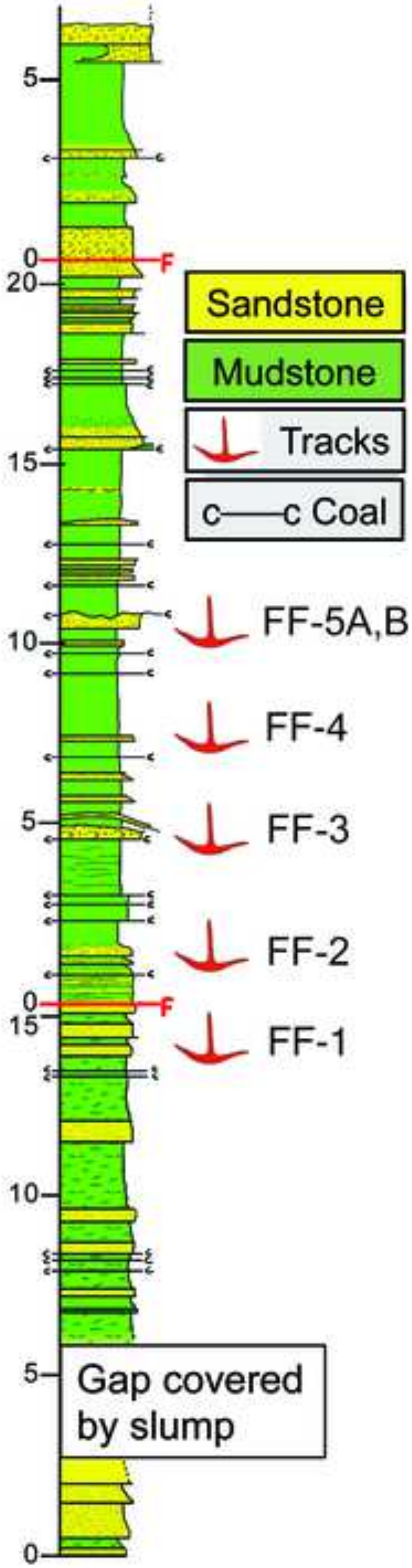

Figure 3

Click here to access/download;Figure;Fig3.tif

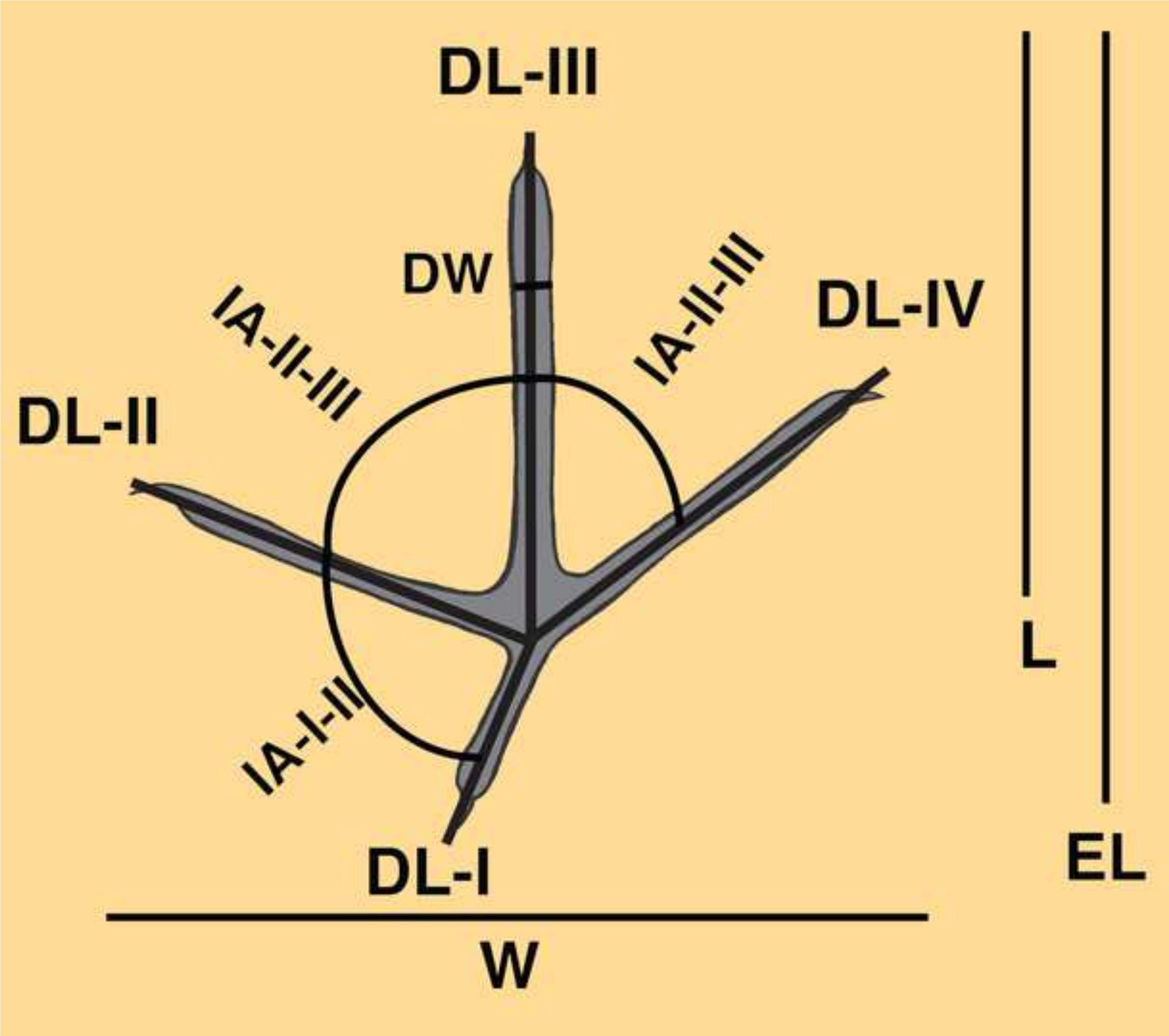

 Click here to access/download;Figure;Fig4.tif

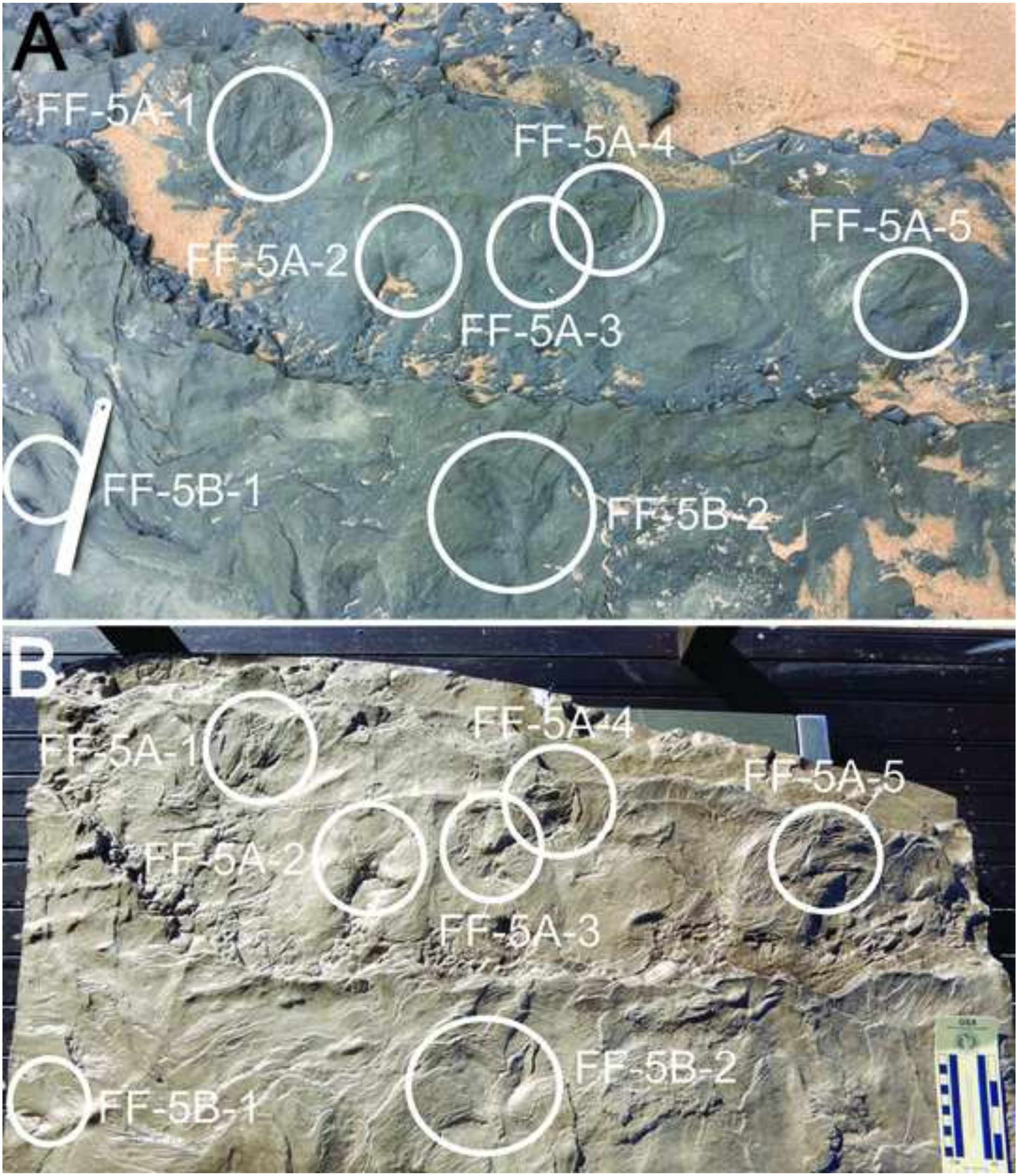

Figure 5

Click here to access/download;Figure;Fig5.tif

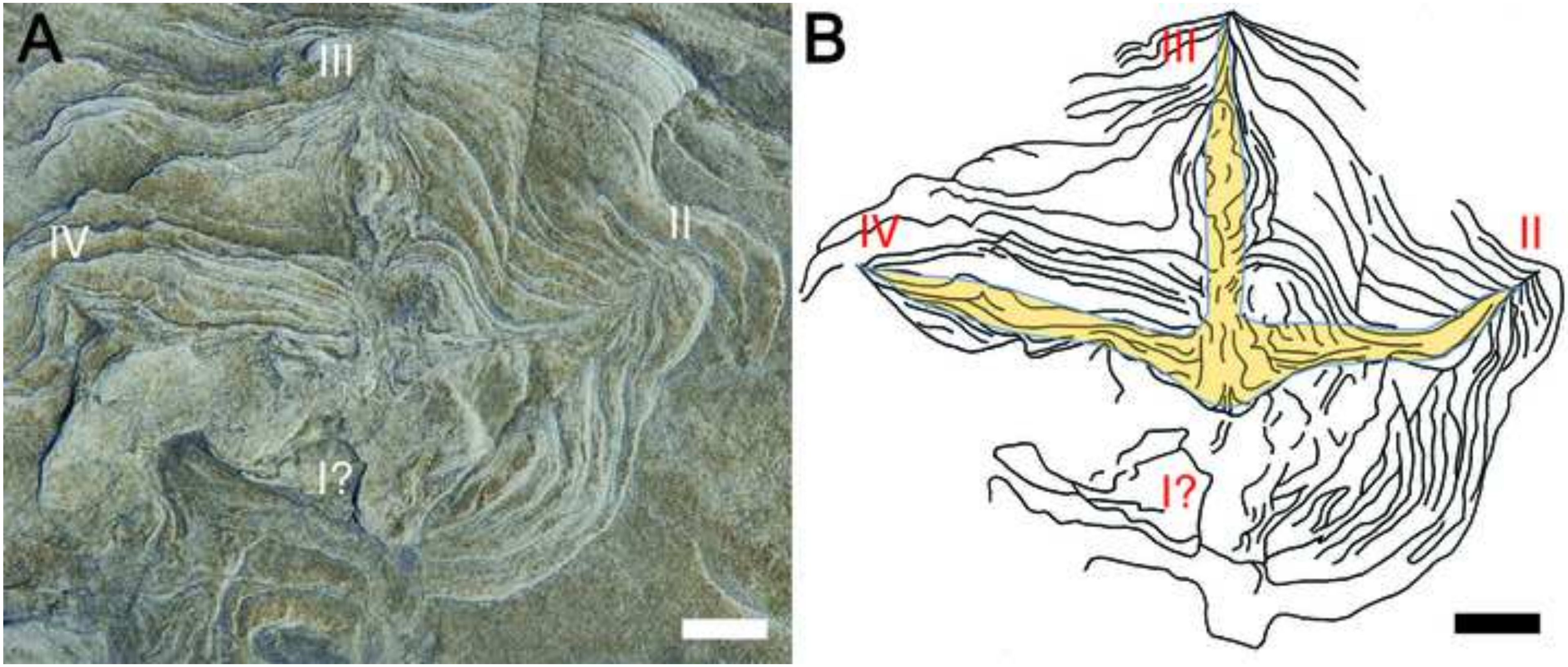

Figure 6

Click here to access/download;Figure;Fig6.tif

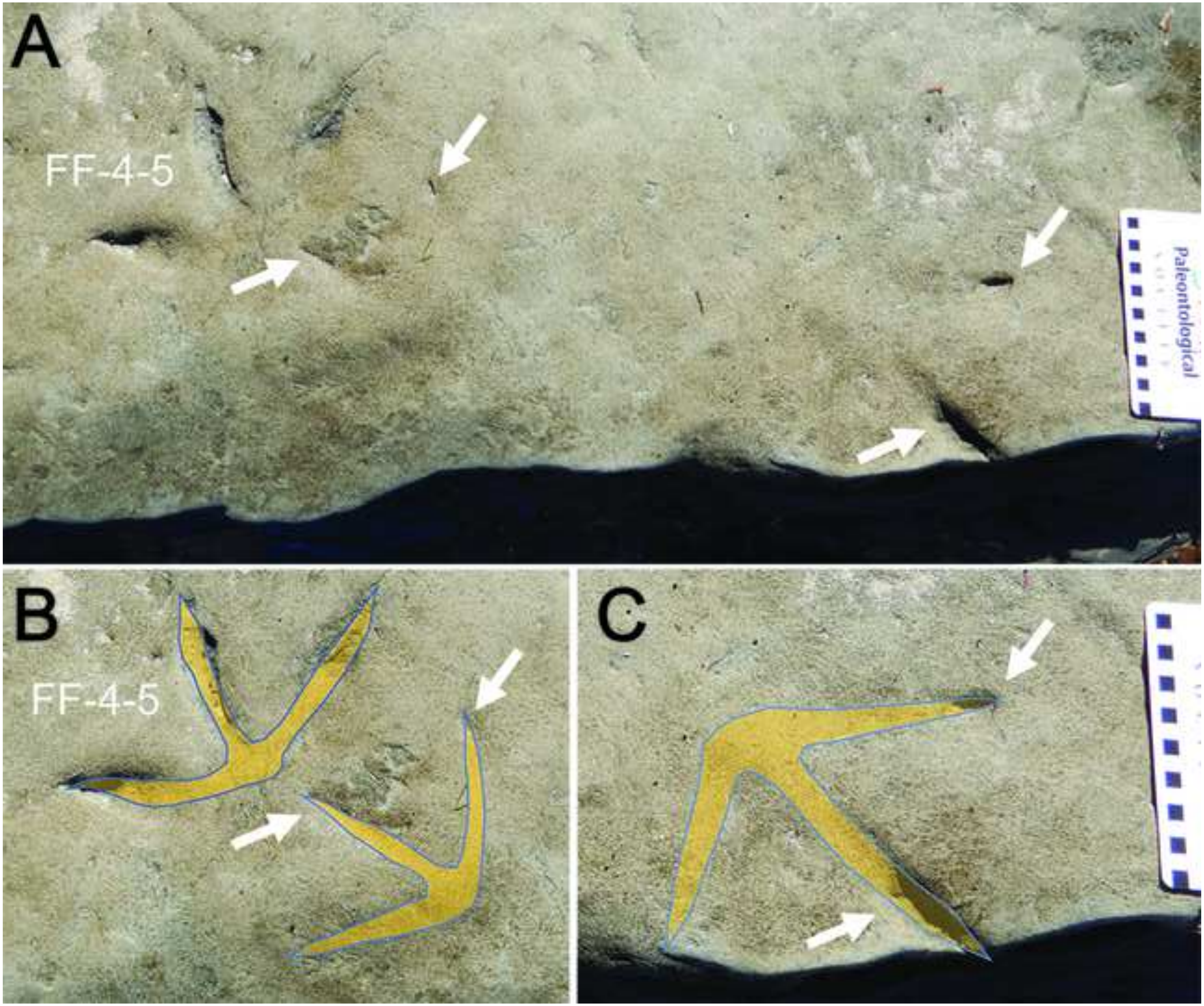

Figure 7 Click here to access/download;Figure;Fig7.tif

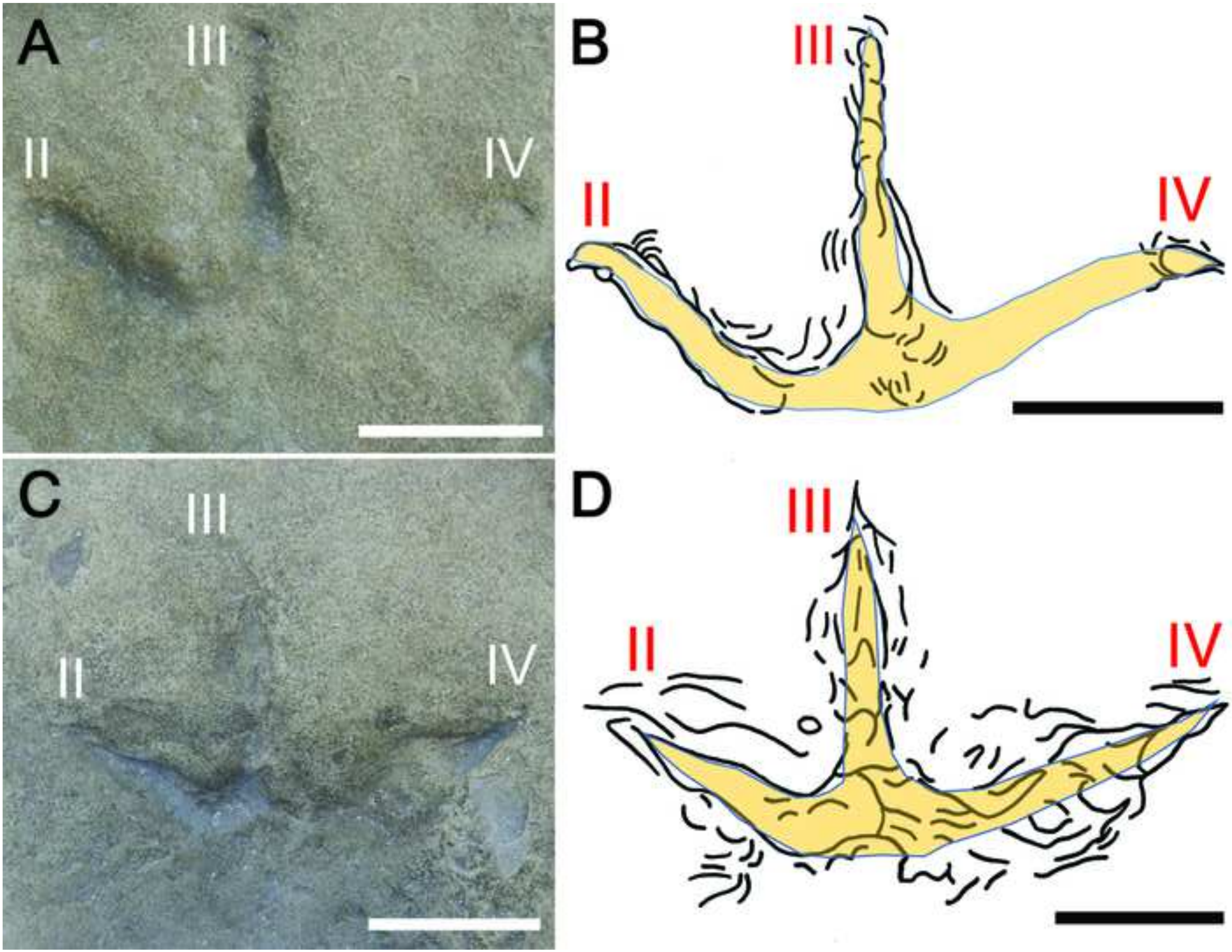

Figure 8

Click here to access/download;Figure;Fig8.tif

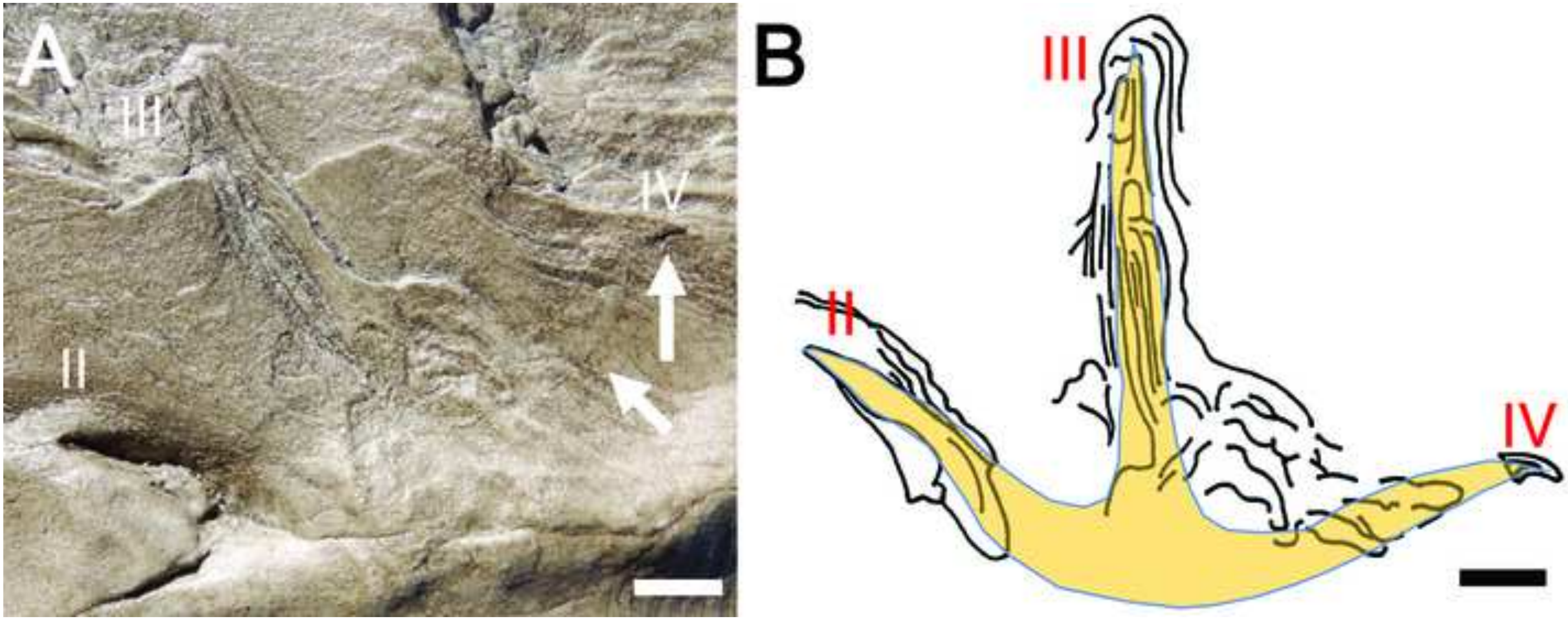

Figure 9 Click here to access/download;Figure;Fig9.tif

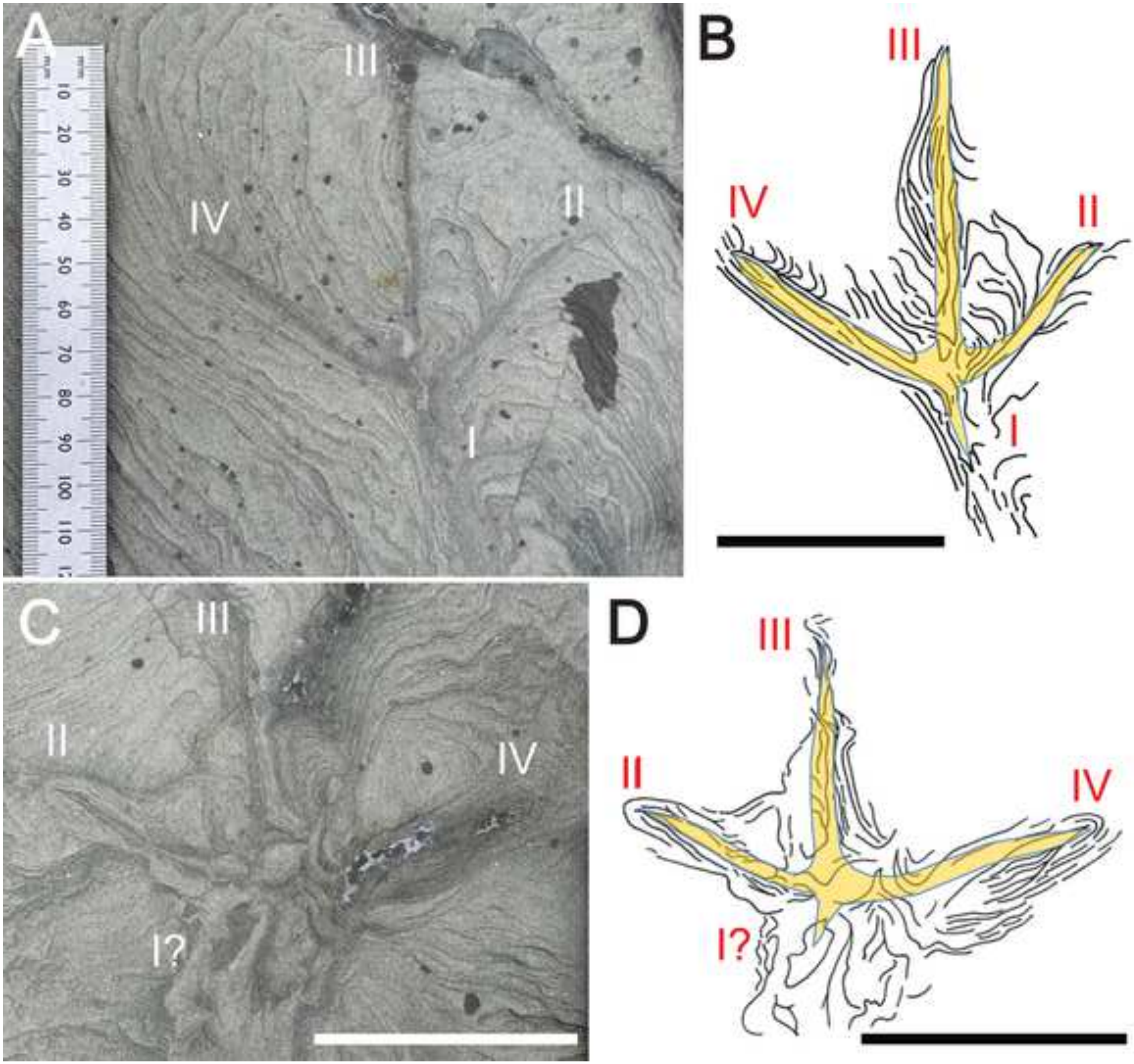

Figure 10

Click here to access/download;Figure;Fig10.tif

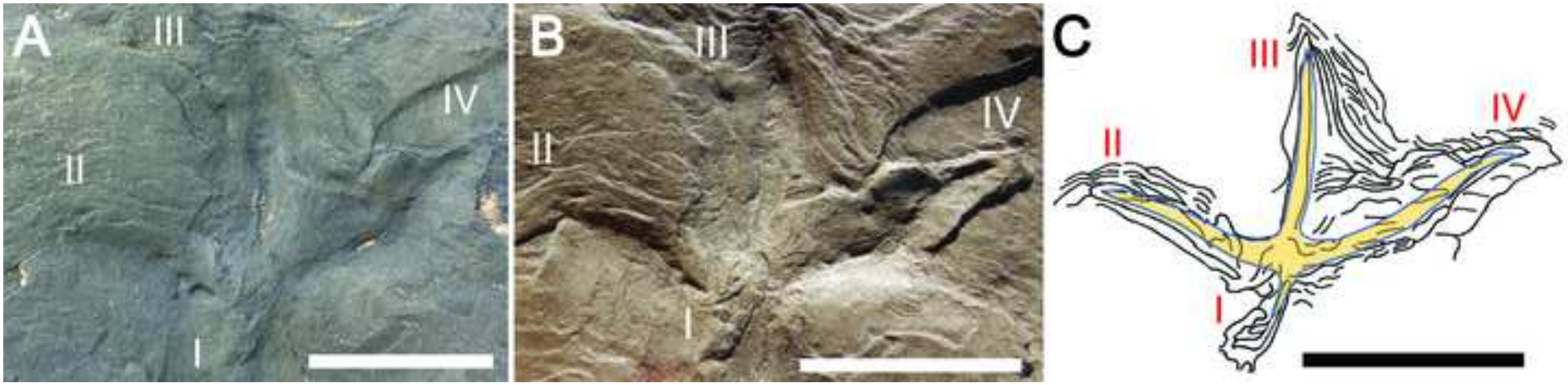

Figure 11

Click here to access/download;Figure;Fig11.tif

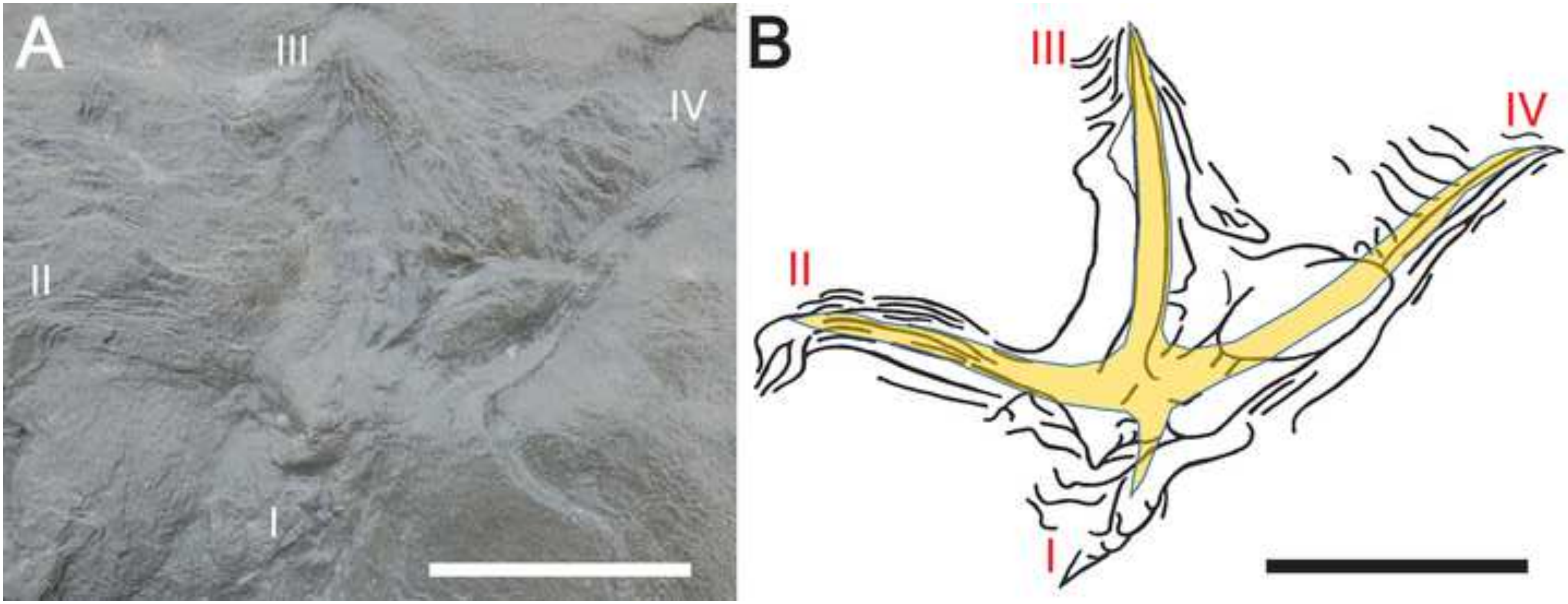

Figure 12

Click here to access/download;Figure;Fig12.tif

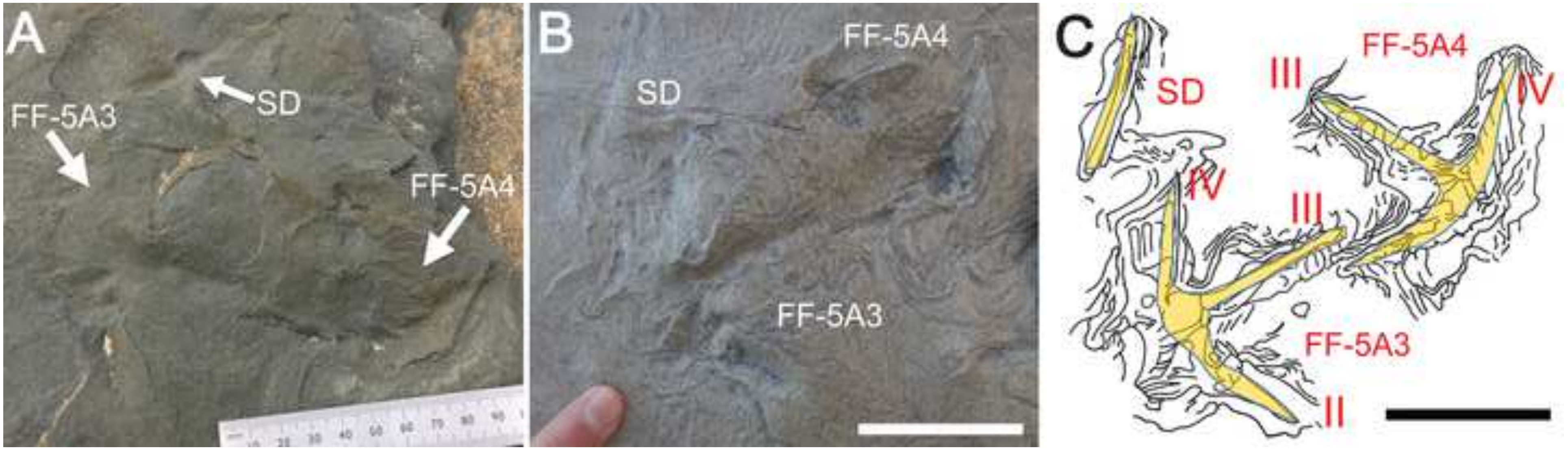

Figure 13

Click here to access/download;Figure;Fig13.tif

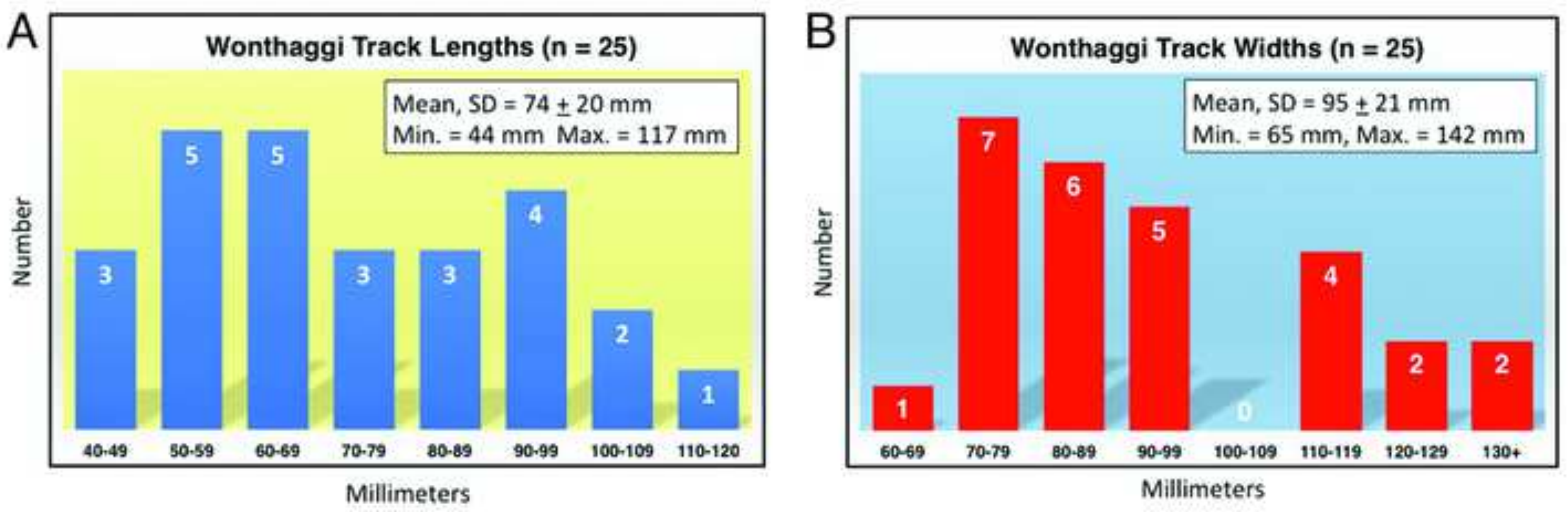

Figure 14

Click here to access/download;Figure;Fig14.tif

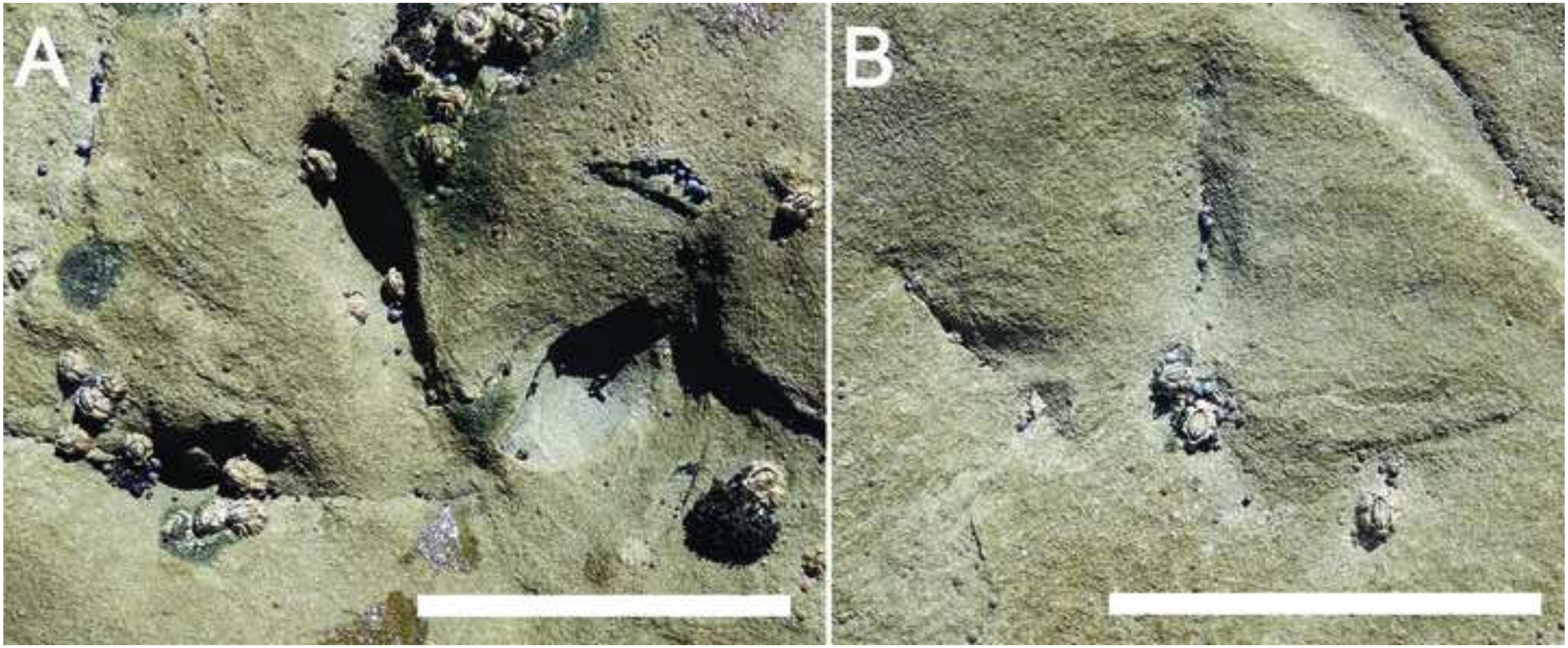

Figure 15 Click here to access/download;Figure;Fig15.tif

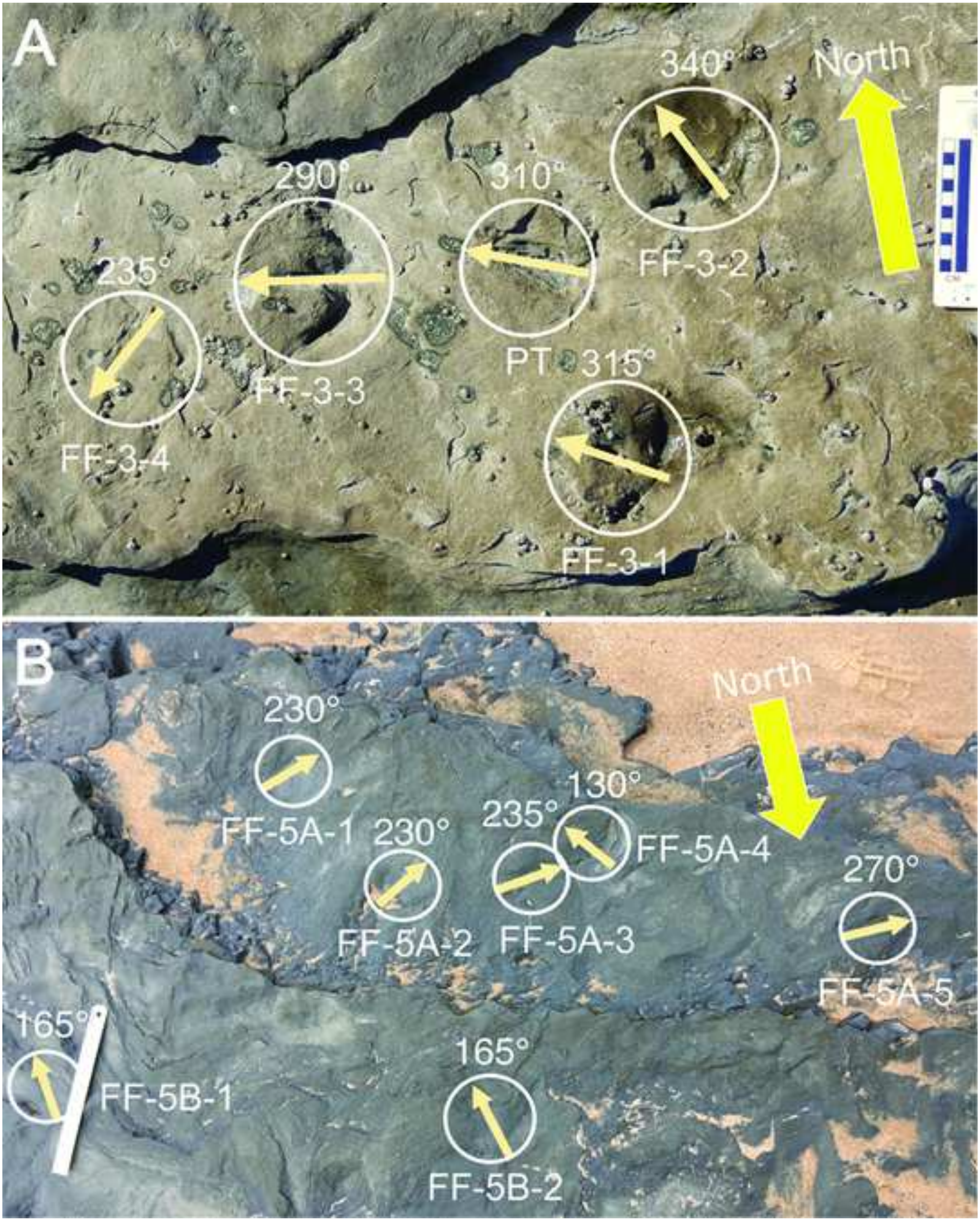

Figure 16

Click here to access/download;Figure;Fig16.tif

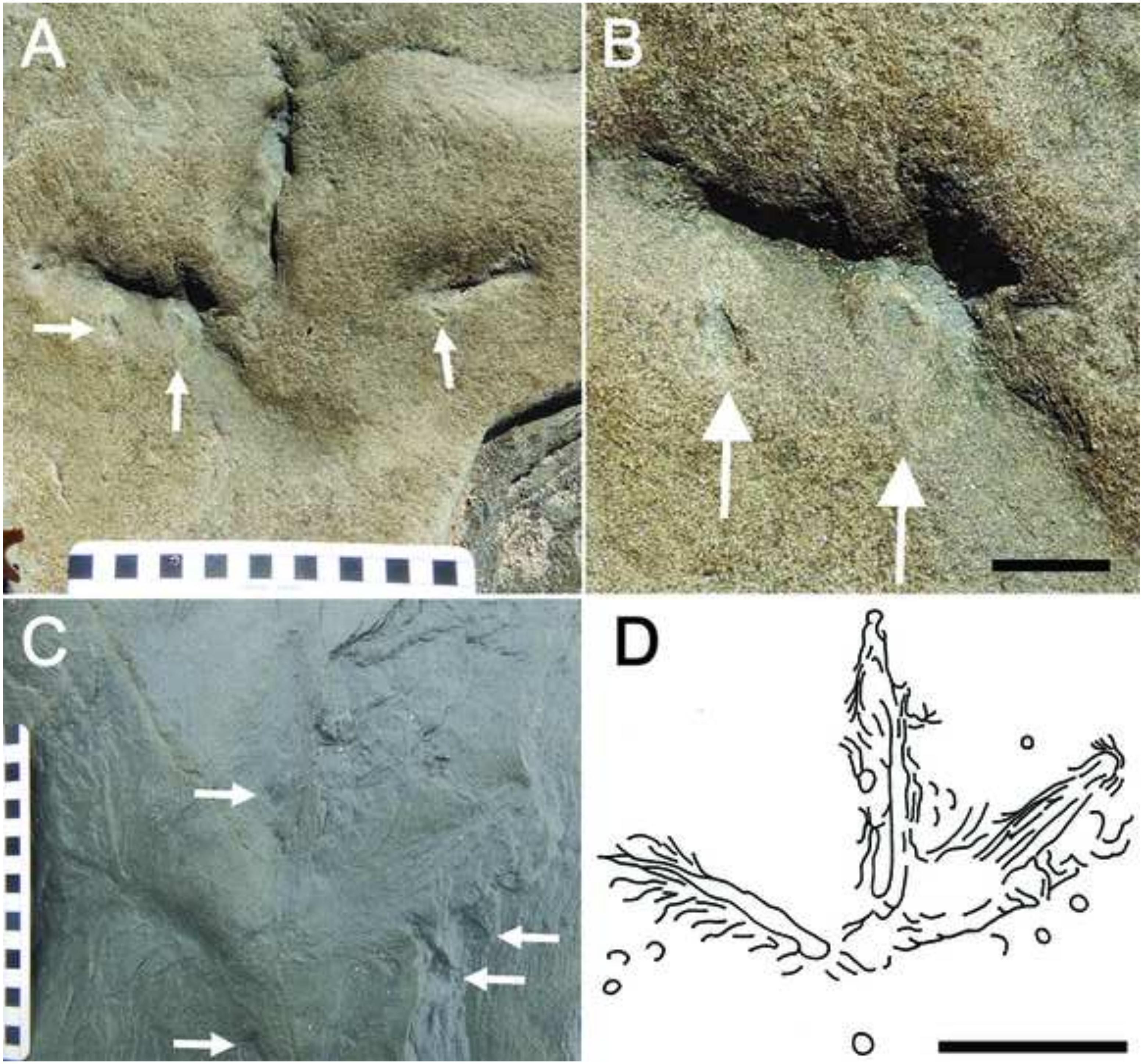

Click here to access/download
Supporting Information
Supporting-Information-1.docx

Supporting Information 1

Click here to access/download
Supporting Information
Supporting-Information-2.pdf
